# A Hydrodynamic Bioreactor for High‐Yield Production of Extracellular Vesicles from Stem Cell Spheroids with Defined Cargo Profiling

**DOI:** 10.1002/advs.202510607

**Published:** 2025-11-19

**Authors:** Solène Lenoir, Elliot Thouvenot, Giacomo Gropplero, Léonie Dec, Damarys Loew, Clotilde Théry, Jose E Perez, Claire Wilhelm

**Affiliations:** ^1^ Laboratoire Physique des Cellules et Cancer PCC, CNRS UMR168 Institut Curie Sorbonne Université PSL Research University Paris 75005 France; ^2^ Institut Curie PSL Research University CurieCoreTech Mass Spectrometry Proteomics Paris 75005 France; ^3^ Institut Curie PSL Research University INSERM U932 and CurieCoreTech EV Paris 75005 France

**Keywords:** bioproduction, extracellular vesicles, hydrodynamic stress, mitochondria, spheroids, stem cells

## Abstract

Extracellular vesicles (EVs) have become pivotal in clinical therapeutics, although no consensus exists on optimal production methods. Here, an innovative approach and associated device are introduced that employ hydrodynamic stress to form and stimulate stem cell spheroids. The method involves culturing cellular material in vessels equipped with internal obstacles, subjected to rotational motion at varying speeds and in alternating reversing modes. It achieves EV yields 10 to 20 times greater than those obtained with traditional static culture methods, offering a robust and high‐output method compatible with large‐scale EV manufacturing. This approach not only streamlines spheroid formation and subsequent EV release into a single, integrated process but also ensures the generation of EVs with enhanced biological activity and a distinctive protein cargo signature. Notably, hydrodynamic stimulation enriched EVs with ectosome‐ and mitochondria‐associated proteins, suggesting distinct biogenesis pathways. Additionally, these EVs demonstrated superior functionality in promoting wound healing, angiogenesis, and anti‐inflammatory responses, underscoring their therapeutic potential.

## Graphical abstract

This study harnesses hydrodynamic flows to generate, confine and stimulate stem cell spheroids, enabling the large‐scale production of extracellular vesicles (EVs). This innovative method not only streamlines spheroid formation and subsequent EV release in a single, integrated process, but also ensures the generation of EVs with enhanced biological activity and a distinct proteomic signature.

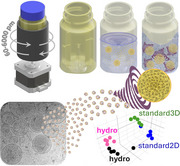

## 1 Introduction

Extracellular vesicles (EVs)^[^ ^1^, ^2^ ^]^ are a heterogeneous group of membrane‐bound particles released by cells, generally classified based on their biogenesis and size into three main subtypes: exosomes (originating from the endosomal system, 50–150 nm), microvesicles (shed from the plasma membrane, 100–1000 nm), and apoptotic bodies (released from dying cells, usually >1000 nm). In most studies—including this one—the term EVs refers primarily to exosomes and microvesicles, as they often overlap in size and cannot be reliably distinguished by standard isolation or characterization techniques. Initially considered cellular waste carriers, EVs are now recognized as key mediators of intercellular communication through the transfer of functional biomolecules from parent to recipient cells.^[^ ^3^ ^]^ Among them, mesenchymal stem cells (MSC)‐derived EVs have emerged as potential therapeutic agents in regenerative medicine.^[^ ^4^, ^5^ ^]^ Indeed, the beneficial effects of MSCs are now largely attributed to paracrine mechanisms, primarily mediated by EVs.^[^ ^6^ ^]^ EVs then serve as carriers of specific bioactive cargo —including proteins, lipids, mRNAs, microRNAs, cytokines— and sometimes DNA ‐that modulate regenerative processes such as immunomodulation, angiogenesis, anti‐apoptotic signaling and tissue repair. By transferring the therapeutic molecular signature of MSCs, EVs offer a cell‐free alternative to conventional cell therapy, reducing risks associated with live‐cell treatments, such as uncontrolled proliferation or differentiation.^[^ ^7^ ^]^ Another notable advantage of EVs is their immune‐privileged status, which facilitates allogeneic use as an off‐the‐shelf therapeutic option. Additionally, the use of EVs is further favored by their improved storage capabilities and shelf‐life compared to whole cells.^[^ ^8^ ^]^

MSC‐derived EVs are now widely recognized for their ability to modulate cellular functions and homeostasis involved in many steps of the cell regenerative process after tissue injury.^[^ ^9^, ^10^, ^11^, ^12^, ^13^, ^14^, ^15^, ^16^, ^17^ ^]^ These include facilitating cell migration and angiogenesis, influencing proliferation, managing cell senescence and differentiation, as well as reducing heart ischemia‐reperfusion injury.^[^ ^18^, ^19^, ^20^, ^21^, ^22^, ^23^, ^24^ ^]^ Given these significant therapeutic perspectives of EVs and their potential for widespread application, one might expect them to already be in clinical use as a replacement for cell therapies. However, the transition of EV‐based therapies from research to clinical practice still faces several challenges in engineering,^[^ ^25^, ^26^, ^27^, ^28^, ^29^ ^]^ production,^[^ ^30^, ^31^ ^]^ characterization and isolation,^[^ ^32^, ^33^, ^34^, ^35^, ^36^, ^37^, ^38^ ^]^ storage,^[^ ^39^ ^]^ and exploitation.^[^ ^40^ ^]^

Concerning production, the primary challenge is to increase the yield of EV release. Various approaches utilizing different modes of stimulation are employed for this purpose. Of these, one of the most widely used methods involves culturing cells under stress conditions, such as hypoxia (< 1% O_2_), nutrient deprivation or temperature changes, which have been shown to boost EV release.^[^ ^41^, ^42^, ^43^, ^44^, ^45^, ^46^ ^]^ Biochemical techniques that incorporate specific growth factors, cytokines or small molecules have also been effective in promoting the release of EVs.^[^ ^47^, ^48^ ^]^ Genetic modification of donor cells to upregulate the expression of EV‐related proteins is another relevant strategy. However, large‐scale cell culture platforms such as hyperflasks or 3D cultures on microcarriers are necessary to increase the yield in order to provide sufficient EVs for clinical use. While these methods perform well,^[^ ^49^, ^50^ ^]^ they remain both resource and time consuming. Recently, the use of physical stimuli, such as shear stress or mechanical agitation, has been introduced to effectively boost the secretion of EVs.^[^ ^51^, ^52^, ^53^, ^54^, ^55^, ^56^ ^]^ This approach is similar to the shear stresses cells incur in our body, such as in blood vessels^[^ ^57^, ^58^, ^59^ ^]^ or during cardiac pumping.^[^ ^60^ ^]^ Initial attempts to trigger EV release through shear stress involved using microfluidic devices with micro‐channels smaller than cells.^[^ ^59^, ^61^, ^62^ ^]^ While these methods enabled rapid EV release, they still faced challenges related to yield and scalability. To increase yield, culturing cells in hollow‐fiber bioreactors is an excellent option,^[^ ^63^, ^64^, ^65^ ^]^ although it requires prolonged production times, resulting in significant delays in generating sufficient EV quantities for clinical use.

The goal of this study is to address these limitations by proposing an alternative hydrodynamic‐based strategy to enhance EV yield and scalability. The rationale is to leverage hydrodynamic cell cultures operating in a high‐shear turbulent regime to stimulate EV release. It demonstrates a significant enhancement of EV release using a specially designed and manufactured device that incorporates an alternating rotation tube with internal obstacles. Beyond the innovative and efficient bioproduction technology proposition, the uniqueness of this strategy lies in the release of extracellular vesicles from human mesenchymal stem cells (hMSCs) organized as spheroids that mimic their native 3D tissue‐like environment, and which can further modulate and enhance the properties of EVs.^[^ ^66^, ^67^, ^68^, ^69^ ^]^ Additionally, the device was tuned to streamline both the formation of spheroids and their subsequent stimulation to trigger a massive release of EVs. To facilitate the adoption of these hydro‐spheroid‐produced EVs and fully realize their potential as a cost‐effective, cell‐free solution, it was essential to assess their cargo and potency. This evaluation included whole proteome analysis, along with functional tests for wound healing, angiogenesis and anti‐inflammatory properties. Hydro‐spheroid‐produced EVs exhibited the typical markers associated with EVs, but they also revealed a distinct hydrodynamic‐derived cargo signature when compared to those produced through starvation, and notably demonstrated enhanced functional properties.

## 2 Results

### 2.1 A Rapid and Efficient Hydrodynamic Method Enhances EV Bioproduction in hMSC Spheroids

**Figure 1:**
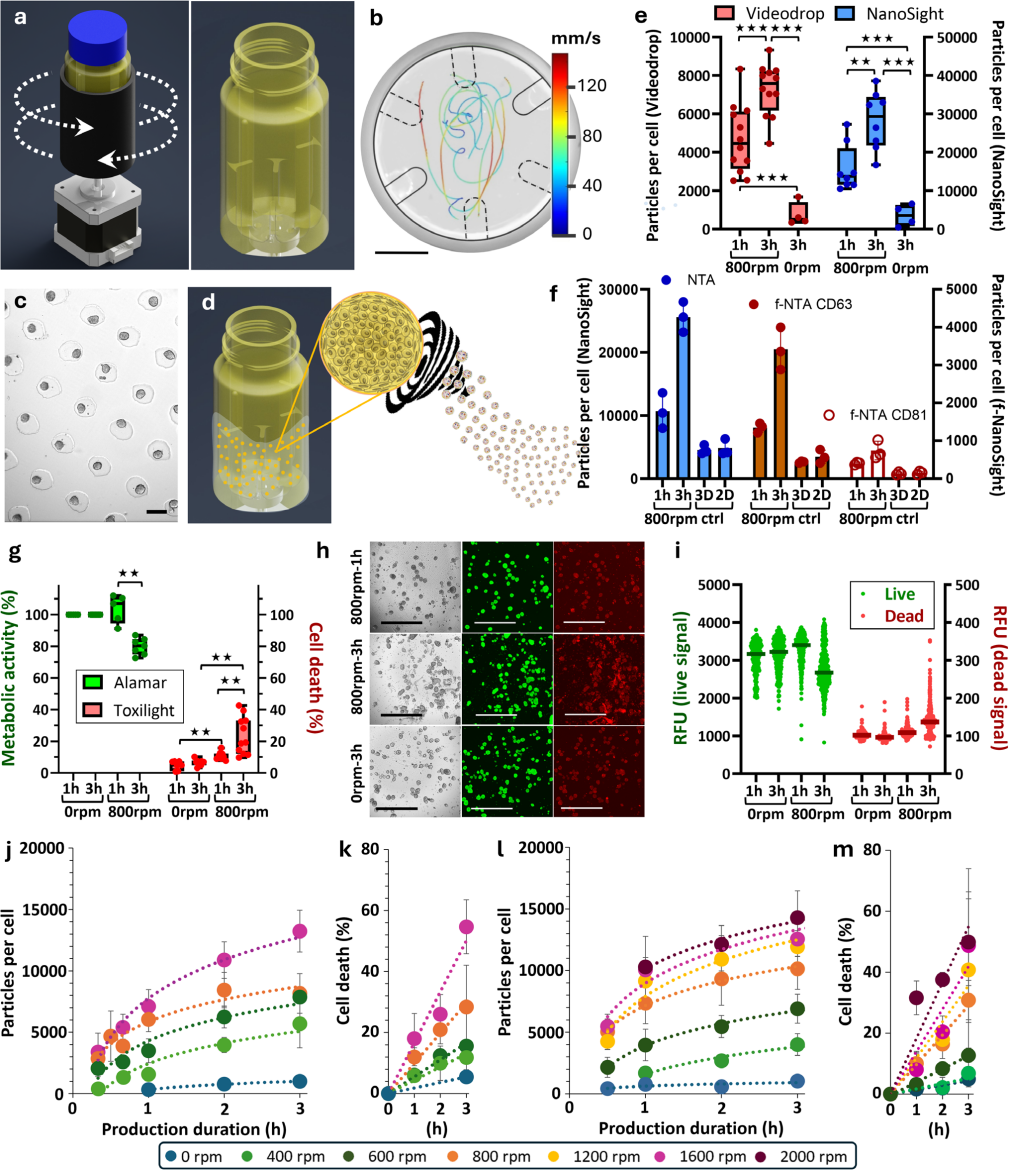
Bioproduction of EVs from hMSCs in rotating tubes. a) Rotating tubes in alternating reversing mode with internal baffles that enhance turbulence within. The tube rotates, stops and then reverses direction. Detailed schematics of the tube are shown in Figure S1a (Supporting Information), and the diagram illustrating the motor setup for simultaneous operation of six tubes is presented in Figure S1c (Supporting Information). b) Tracking the movement of phosphorescent beads in a plane of the rotating tube (800 rpm) allows for direct visualization of the turbulent flow. Scale bar = 1 cm. c) Bright field imaging of the agarose microwell array that allows for spheroid formation before placement into the tubes. Scale bar = 200 µm. Each spheroid forms in an individual microwell, starting from 250 individual cells, resulting in spheroids with an average diameter of 125 ± 20 µm. This average diameter was measured directly within the microwells, with 50 diameter measurements taken for each independent spheroid formation, across a total of 6 independent experiments. d) Schematic of spheroids trapped in the flow, with vortex formation generating intense flow stresses on each spheroid, leading to the production of many EVs. e) Measurements of the number of particles produced per cell for 1 h or 3 h of hydrodynamic stimulation (800 rpm) or 3 h under static conditions (0 rpm). Each point represents an independent biological replicate, and each condition was measured by NTA using both the Videodrop and NanoSight instruments. f) Quantification of particles by fluorescent nanoparticle tracking analysis (f‐NTA). Particles were quantified using both standard NTA (scatter mode, total particle count) with the NanoSight instrument and fluorescence NTA (f‐NTA) following labeling with anti‐CD63‐520 and anti‐CD81‐520 antibodies (Particle Metrix). Fluorescent NTA enables the specific detection of EV subpopulations expressing tetraspanins, key markers associated with MISEV guidelines. Prior to analysis, particles were concentrated ≈100‐fold from conditioned medium by filtration and incubated with antibodies as described in Methods. Particle concentrations are expressed as particles per producing cell, after correction for the concentration/dilution factors. For each detection mode (total, CD63⁺, and CD81⁺), three independent production experiments were analyzed (each dot represents one independent production). Quantifications were performed for the following conditions: Hydrodynamic stimulation at 800 rpm for 1 h and 3 h (spheroids), 3D control: spheroids in starvation without rotation, 2D control: adherent MSCs in starvation for 72 h (reference condition for standard EV production). g) After 1 h or 3 h of production under hydrodynamic (800 rpm) or static (0 rpm) conditions, each condition was measured using ToxiLight to assess cell mortality and AlamarBlue to evaluate metabolic activity. Each point represents an independent biological replicate. h) Live (green) and dead (red) imaging of spheroids after 1 h or 3 h at 800 rpm or 0 rpm, revealing minimal impact on live and dead signals following hydrodynamic stimulation. Scale bars = 1 mm. i) Quantification of the live and dead signal for the different conditions, with each point corresponding to measurements on an individual spheroid. j,k) Kinetic curves of particle production per cell (organized in spheroids) measured by Videodrop (j) and spheroid mortality measured by ToxiLight (k) for four conditions: 0, 200, 400, 800, and 1600 rpm. Each point represents the mean, and the error bars indicate standard deviation, with n > 3 for all conditions. l,m) Similar curves of particle production per cell (l) and mortality (m) as a function of production time, but for production from individual cells in suspension, not yet organized into spheroids, with n > 3 for all conditions, and for a broader range of conditions: 0, 200, 400, 800, 1200, 1600, and 2000 rpm.

The objective was to design a device to explore ways of controlling hydrodynamic shear flow‐mediated bioproduction of EVs from human stem cells organized in a spheroid 3D biomimetic environment. Such process involves creating a turbulent flow with vortices that trap spheroids, subjecting them to high hydrodynamic stresses that stimulate the production of EVs. The method (patent publication number WO2024/2005070) employs rotating tubes featuring turbulence‐enhancing inner obstacles and operating in an alternating reversing mode (**Figure** **1**a), which introduces an additional layer of shear flow stress. The design of the tube is fully modular, including the internal obstacles. Two configurations were tested and 3D printed, including inner walls and grids structures (Figure S1, Supporting Information). Both performed efficiently (Figure S2, Supporting Information), so the baffled design (with inner walls) was selected as one that could be manufactured from plastic by injection molding, offering the advantages of high‐throughput production, easy sterilization and optimal biocompatibility. It features three pairs of inner walls oriented at 120° from each other, with increasing heights of 1, 2 and 4 cm, in a tube with a diameter of 3.7 cm and a total height of 8.4 cm. A Reynolds number can be estimated for this rotating baffle geometry, as introduced in the Methods section, with values ranging from ≈2600 (at 200 rotations per minute, rpm) to 26 000 (at 2000 rpm), indicative of a turbulent flow regime. Moreover, the tube's geometry enables real‐time tracking of flow dynamics by particle image velocimetry (Figure 1b; Figure S3, Supporting Information). Average velocities are found in the range of 40, 80, and 160 mm s^−1^ for rotations at 400, 800, and 1600 rpm, respectively.

With this specific design, the tube holds between 20 and 30 mL, which corresponds to a two‐thirds fill level. It is filled with medium containing the cellular material, prepared strictly without serum for the production stages. Figure 1d illustrates the interior of the tube when it contains spheroids that were previously formed through micropatterning (Figure 1c). The spheroids were generated in 200 µm agarose‐made microwells within the wells of a 6‐well plate, each containing 2000 agarose microwells. A total of 500 000 hMSCs were used per well, and the cells matured over 2 days to form dense spheroids (Figure S4, Supporting Information). The spheroids were then resuspended in serum‐free medium at a concentration of 2000 spheroids per mL (comprising all spheroids from a single well in one mL), enabling one tube to hold the equivalent of the content from four entire 6‐well plates. As proof of concept, Figure 1e shows the number of particles released per cell (accounting for all cells within the spheroids) during stimulation for either 1 h or 3 h at 800 rotations per minute (rpm), with a direction reversal period of 5 s. This production is compared with the measurements obtained after 3 h under static conditions (the same spheroids in a non‐adherent flask at the same density), resulting in an almost 20‐fold increase due to hydrodynamic stimulation. This measurement of the number of particles produced was conducted using nanoparticle tracking analysis (NTA) techniques, utilizing either the Videodrop (Myriade) instrument or the NanoSight (Malvern) instrument. The NanoSight has a lower detection limit of 50 nm, compared to the Videodrop, which has a limit of 80 nm. This accounts for the greater number of particles measured by the NanoSight. Furthermore, Figure S5 (Supporting Information) demonstrates linear correlation across all measurements conducted in this study on the same samples using both instruments, with an average of 4 times more particles measured by the NanoSight. Distributions obtained with the two instruments, shown in Figure S6 (Supporting Information) and derived from independent batches, illustrate the low variability across replicates, as well as systematic differences in measured diameters —Videodrop yielding larger average sizes, in line with its lower particle detection sensitivity. To compare with other bioproduction techniques, the values provided by the NanoSight, which is the most used instrument, offer a useful reference, though care must be taken due to potential variability across laboratories in settings and instrumentation. As an initial benchmark, hydrodynamic stimulation yields ≈30 000 particles per cell, as measured by NanoSight, placing it at the high end of current bioproduction methods.^[^ ^70^ ^]^ Here are some examples, though far from exhaustive: In hyperflasks, bone marrow stem cells (BM‐MSCs) produce ≈6000 EVs per cell over 48 h.^[^ ^71^ ^]^ Using umbilical cord‐derived MSCs (UC‐MSCs), hollow fiber systems yield an estimated 100 EVs per cell per harvest per few‐days harvest.^[^ ^72^ ^]^ These cultures can be maintained for over 50 days, enabling multiple harvests and ultimately producing over 1000 EVs per cell in more than 1000 h. Scaling‐up of hollow fibers systems has been achieved with Quantum bioreactors, reaching ≈15 000 EVs per BM‐MSC over several days.^[^ ^64^, ^73^ ^]^ Hydrodynamic‐based approaches utilizing shear flow stresses such as vertical wheel bioreactors^[^ ^49^, ^66^ ^]^ and spinner flasks^[^ ^74^, ^75^, ^76^ ^]^ can push EV yields up to 100–3000 EVs cell^−1^ h^−1^, or up to 10000 EVs cell^−1^ h^−1^ when under higher shear flow stresses.

To ensure that the increase in particle numbers observed with hydrodynamic stimulation is not simply due to an accumulation of cellular debris or non‐EV particles, we performed fluorescent nanoparticle tracking analysis (f‐NTA) targeting specific EV markers. While previous comparisons were based on brightfield NTA, which detects all nanoparticles irrespective of their nature, f‐NTA allows the selective quantification of EVs expressing surface markers such as CD63 and CD81. Independent EV production experiments (n = 3 per condition) were carried out using spheroids subjected to hydrodynamic stimulation at 800 rpm for either 1 h or 3 h. These were compared to two non‐stimulated controls: spheroids maintained in starvation conditions (3D starvation) and standard 2D monolayer cultures also subjected to serum starvation for 72 h—the reference method for conventional EV production. EVs were first concentrated ≈100‐fold by filtration, then labeled with CD63‐520 and CD81‐520 antibodies according to the protocol detailed in Methods. Fluorescent NTA was performed on the NanoSight NS300 instrument equipped with a 532 nm laser, allowing excitation of the fluorophores. As shown in Figure 1f, hydrodynamic stimulation for 3 h led to more than a fivefold increase in total particle numbers per producing cell compared to the standard 2D control. Importantly, this fold‐change was preserved when quantifying CD63⁺ and CD81⁺ EVs, confirming that the stimulation protocol enhances the secretion of EVs rather than merely increasing unspecific particle content. These results further support the efficacy of the hydrodynamic approach in achieving rapid and high‐yield EV production.

Figures 1g,i then focus on the direct impact of hydrodynamic stimulation on the viability of spheroids, the initial mandatory step in evaluating an EV bioproduction technology. Metabolic activity was measured using resazurin metabolic conversion to resorufin (Alamar Blue) after 1 h or 3 h of production under static control conditions and under rotation condition (800 rpm), expressed as a percentage of the initial activity. The results showed a slight decrease in metabolic activity after 3 h, approximately by 20%. Concurrently, cell death was assessed by measuring the release of the enzyme adenylate kinase from damaged cells (ToxiLight assay). There was a good correlation with metabolic activity, showing ≈20% cell mortality. These findings were corroborated by a live/dead assay at the spheroid level (Figure 1h,i), which indicated a slight decrease in green calcein signal (intracellular esterase activity of live cells) and a corresponding increase in red ethidium signal (loss of plasma membrane integrity in dead cells) after 3 h of production. Lastly, we performed a caspase 3/7 activation assay to evaluate a possible apoptotic outcome on the producer cells due to the hydrodynamic stress (Figure S7, Supporting Information). The levels of caspase were found to be similar for all rotation conditions tested compared with spheroids under no rotation (0 rpm), with all conditions showing significantly lower caspase activation compared to spheroids subjected to hyperthermia as a positive control. All these results taken together, we limited production times to below this 3‐h threshold.

All subsequent measurements will be reported using Videodrop measurements to assess yield and the ToxiLight assay to assess cell death. As just demonstrated, both methods were validated relative to other techniques. They provide the only viable procedure for evaluating numerous stimulation parameters of the bioproduction process. Indeed, Videodrop allows for much quicker measurements compared to NanoSight (30 s vs 5 min), requiring only 8 µL of the conditioned medium (CM) per measure, while the ToxiLight assay can also be performed using only 5 µL of the CM per measure. Thus, Figure 1j illustrates the particle production per cell measured by Videodrop as a function of production time and intermittent rotation speed, while Figure 1k shows the same conditions but in terms of mortality assessed by ToxiLight. There is a kinetic effect, with production increasing linearly during the first hour before gradually saturating, whereas cell mortality rises linearly with production time and further escalates with increasing rpm.

The final step in the proof of concept for the production method was to initiate production directly with suspended individual cells, bypassing the spheroid formation stage in the microwells. This corresponds to Figure 1l (particles per cell) and Figure 1m (mortality), which demonstrate similar viability—albeit slightly lower—and, most importantly, comparable production rates, while in this condition with individual cells, all cells are accessible to the flow. Spheroids thus produced as many EVs per cell as single cells in suspension, suggesting that the spheroid core still plays a role in EV release.

We selected four different conditions for further investigation: Starv2D, Starv3D, HydroCell, and HydroSph. The Starv2D condition refers to the classical control of 72 h of starvation in adherent 2D cultures (0 rpm – 2D). The Starv3D condition involves spheroids placed on non‐adherent substrates, identical to those used in rotation stimulation, also subjected to 72 h of starvation (0 rpm – 3D). The HydroCell and HydroSph conditions involve 3 h of hydrodynamic stimulation at 600–800 rpm, with HydroCell starting from individual cells in suspension and HydroSph from spheroids. At the end of each stimulation, conditioned media, previously analyzed for particle yield were concentrated and purified using size‐exclusion chromatography (SEC) to effectively separate EVs from soluble factors. For all subsequent characterization and functional assays, EVs were systematically isolated using SEC.

**Figure 2:**
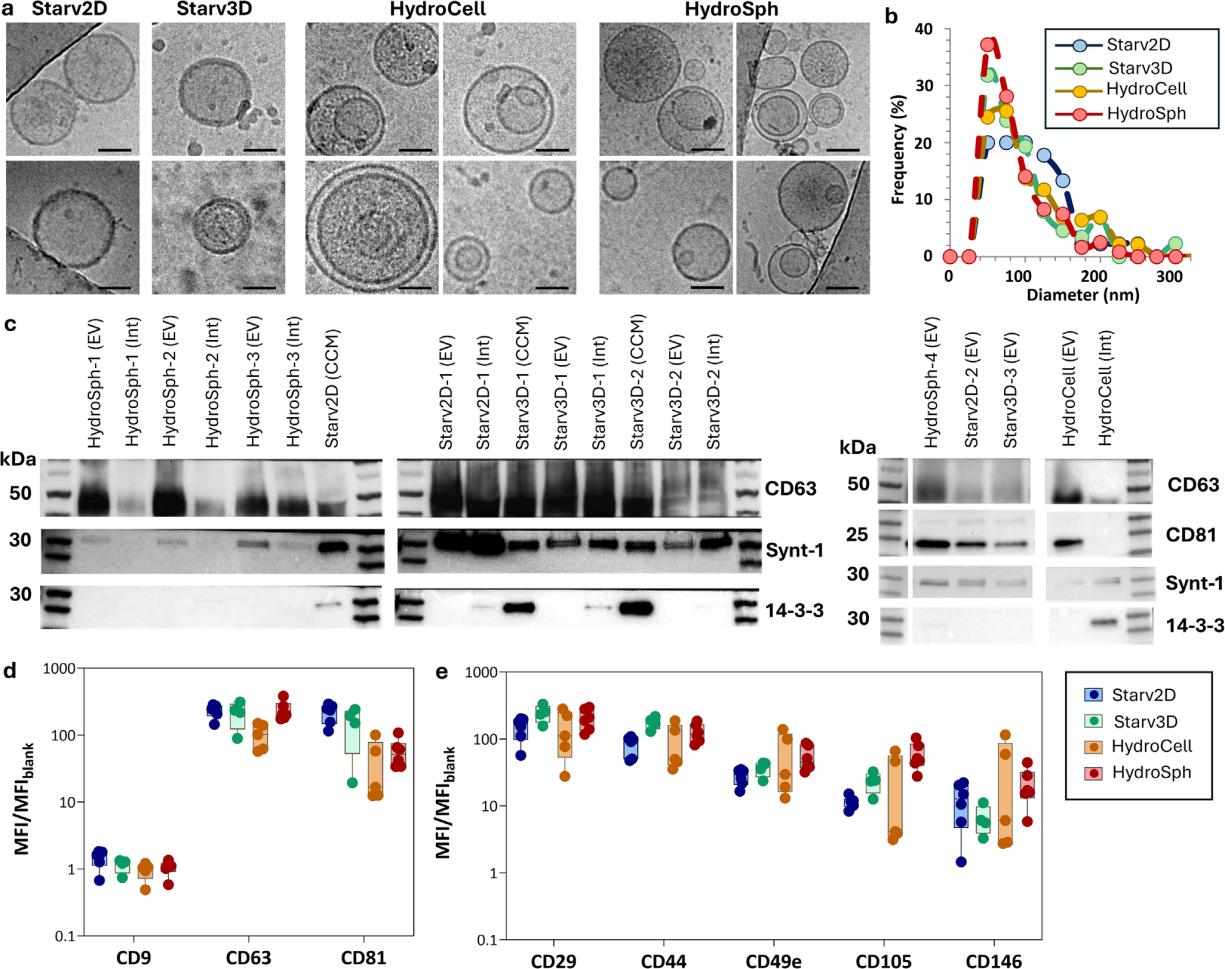
EV identity confirmed by structural and protein markers. a) Cryogenic transmission electron microscopy imaging of EVs produced from spheroids (600 rpm, HydroSph) or individual cells (600 rpm, HydroCell) in hydrodynamic conditions, and for EVs produced upon serum starvation from spheroids (0 rpm Starv3D) or adherent cells (0 rpm Starv2D). Scale bars = 50 nm. b) Diameter distributions extracted from cryoTEM imaging (n = 45 for Starv2D, n = 87 for Starv3D, n = 120 for HydroSph, n = 190 for HydroCell). c) Western blot analysis of specific EV membrane (CD63, CD81) and cytosolic (Syntenin‐1) markers, along with a co‐isolated non‐EV marker (14‐3‐3) for EVs produced from spheroids (HydroSph) or individual cells (HydroCell) in hydrodynamic conditions, and for EVs produced upon serum starvation from spheroids (Starv3D) or adherent cells (Starv2D). In the first set of Western blots (left), CD81 was not probed, whereas it was included in the second set (right). d,e) Bead‐based fluorescence flow cytometric analysis of EV‐specific (d) and mesenchymal‐specific (e) protein markers in extracellular vesicles produced from spheroids (HydroSph) or individual cells (HydroCell) in hydrodynamic conditions, and for EVs produced upon serum starvation from spheroids (Starv3D) or adherent cells (Starv2D). Results are presented as ratios of mean fluorescence intensity with respect to blank. Each point corresponds to an independent experiment (n = 5).

The EVs produced from each condition were subsequently observed by cryo‐electron microscopy for detailed visualization of vesicular morphology (**Figure** **2**a). Additional images for these conditions are provided in Figures S7–S12 (Supporting Information), with over 40 individual EVs examined per condition. All observed vesicles were intact, and their diameters (Figure 2b) revealed comparable size distributions, with an average diameter of 85 nm. Western blot analysis (Figure 2c) and MACSPlex profiling (Figure 2d,e) were jointly employed to confirm the EV identity and purity of the isolated particles across all production conditions (Starv2D, Starv3D, HydroCell, HydroSph), following the guidelines outlined in the Minimal Information for Studies of Extracellular Vesicles (MISEV2023).^[^ ^77^ ^]^

Western blotting of purified EV fractions demonstrated the presence of the EV‐specific transmembrane domain proteins CD63 and CD81 (MISEV category 1) and the cytosolic protein Syntenin‐1 (MISEV category 2), while non‐EV‐associated contaminants such as 14‐3‐3 proteins were absent, supporting the specificity and purity of the isolates. Complementary MACSplex analysis,^[^ ^78^ ^]^ a bead‐based multiplex assay allowing for simultaneous surface marker quantification, confirmed the co‐expression of CD63 and CD81 on the same EVs in all conditions. As expected, CD9 was not detected, reflecting its low or absent expression in human MSCs and thus serving as a negative control. Additionally, mesenchymal‐associated surface markers including CD29, CD44, CD49e, CD105, and CD146 were all detected, further supporting the MSC origin of the vesicles. Each condition was analyzed with five independent biological replicates, ensuring reliability and reproducibility of the data.

### 2.2 EVs Generated under Hydrodynamic Flow Exhibit Typical EV Proteomic Markers that only Partially Overlap with Those of Starvation‐Derived EVs

**Figure 3:**
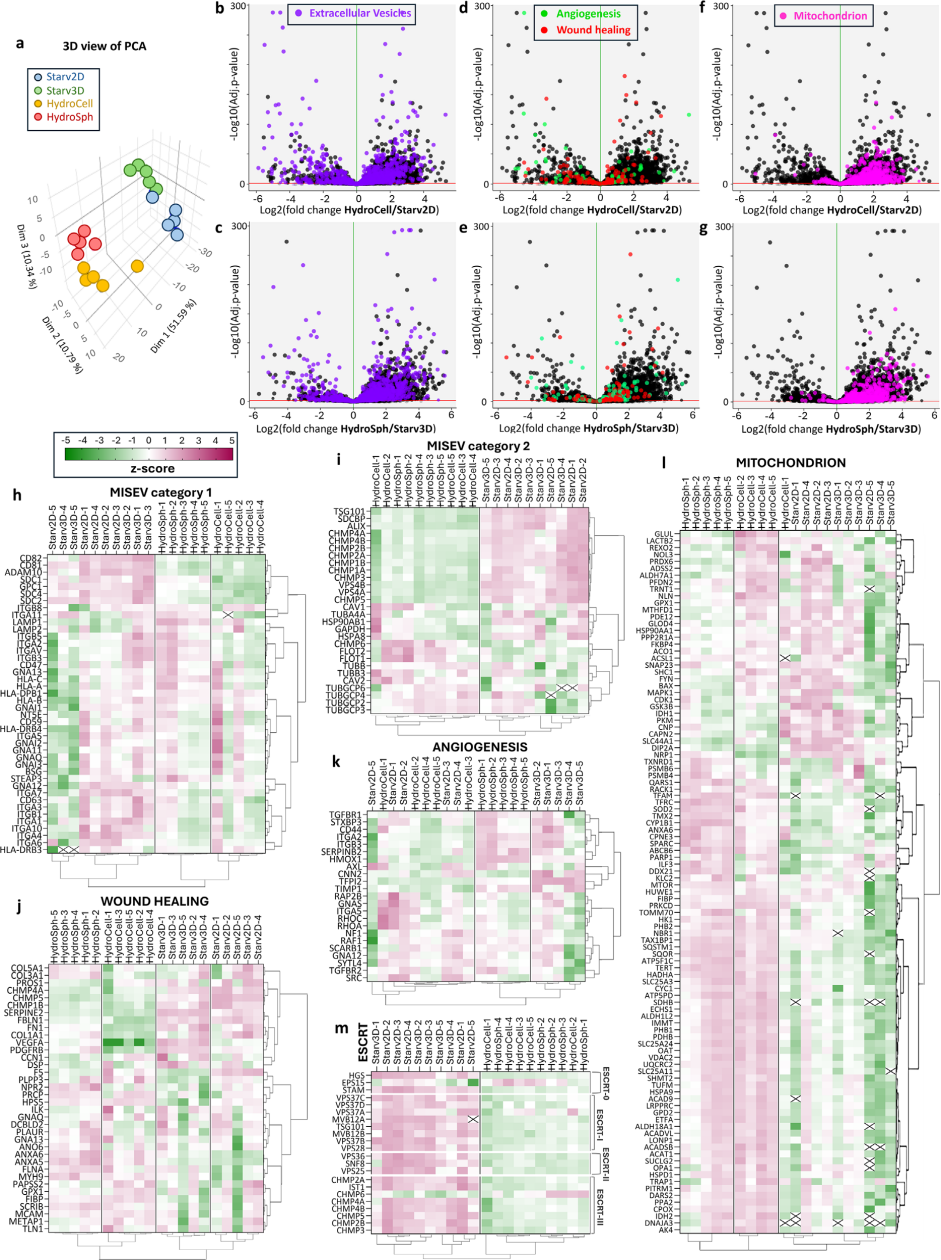
Proteome characterization by mass spectrometry‐based proteomics. a) 3D view of Principal Component Analysis (PCA) of the proteome for conditions Starv2D (blue), Starv3D (green), HydroCell (yellow), and HydroSph (black). b–g) Proteome comparison between conditions depicted in volcano plots. Proteins associated with the “extracellular vesicle” GO term (GO:1903561) are shown in purple, on top of all proteins quantified in both conditions (black). Comparisons are made between Starv2D and HydroCell (b‐), and Starv3D and HydroSph (c). Proteins associated with the “wound healing” GO term (GO:0042060) and “angiogenesis” GO term (GO:0001525) are represented in red and green, respectively, and are shown for comparisons between Starv2D and HydroCell (d), or Starv3D and HydroSph (e). Proteins associated with the “mitochondrion” GO term (GO:0005739) are shown in pink for comparisons between Starv2D and HydroCells (f), and Starv3D and HydroSph (g). h–l) Heatmaps with hierarchical clustering of EVs produced through serum starvation from hMSC in 2D configuration (Starv2D) and 3D configuration (Starv3D) and EVs produced from individual hMSCs (HydroCell) or hMSC‐derived spheroids (HydroSph) stimulated hydrodynamically in the reverse rotating tubes. Proteins are listed on the left side of each heatmap. The heatmaps display Z‐scores for all proteins found, including transmembrane EV proteins as described in the MISEV category 1 (h), cytosolic EV proteins as described in the MISEV category 2 (I), for proteins related to wound healing (j), angiogenesis (k), mitochondria (l), and ESCRT‐related proteins (m).

We investigated the protein content of EVs from the four Starv2D, Starv3D, HydroCell, and HydroSph conditions, each produced as five biologically independent replicates, using label‐free mass spectrometry‐based proteomics. Size distributions from the independent batches produced under each condition showed comparable dispersion and mean diameters, as presented in Figure S13 (Supporting Information). The protein content was measured for the five independent replicates, with values expressed as the number of particles per µg of protein. All values were found to be in the range of 10^8^, specifically (0.9 ± 0.2) × 10^8^ for Starv2D, (1.1 ± 0.6) × 10^8^ for Starv3D, (1 ± 0.3) × 10^8^ for HydroCell, and (1.1 ± 0.3) × 10^8^ for HydroSph. Proteins with at least 3 distinct peptides were identified, with 6046 detected in Starv2D, 5972 in Starv3D, 6507 in HydroCell, 6498 in HydroSph, while 5737 were common across all groups. Principal component analysis (PCA) was performed to assess differences in protein composition among the groups (**Figure** **3**a). This unbiased method clearly distinguished different EV populations, with hydrodynamic conditions clustering distinctly from the starvation conditions and further separation observed between 2D and 3D environments. Hierarchical clustering analysis based on normalized protein intensities is represented for all proteins and samples in Figure S14 (Supporting Information). Gene ontology enrichment analysis was performed for the protein lists of each cluster compared to the entire human proteome. The four Gene Ontology (GO) terms identified in this study were extracellular vesicles (1133 proteins), wound healing (156 proteins), angiogenesis (124 proteins), and mitochondria (730 proteins). These proteins are presented in the volcano plots of Figure 3b–g, alongside all proteins across all replicates. For these volcano plots, proteins were selected based on the presence of at least two distinct peptides in at least one replicate across all conditions, and an adjusted *p*‐value of less than 0.05. The conditions were compared pairwise: the Starv2D condition, with individual adherent monolayer cells under starvation, was compared with the HydroCell condition, with individual cells under hydrodynamic stimulation (Figure 3b,d,f); and the Starv3D condition, with spheroids under starvation, was compared with the HydroSph condition, with spheroids under hydrodynamic stimulation (Figure 3c,e,g). Proteins with a log2(fold change) between ‐2 and 2 were considered present in similar amounts (no significant enrichment). For the HydroCell and HydroSph conditions, enrichment was considered significant for a log2(fold change) > 2, while for Starv2D and Starv3D conditions, it was considered significant for a log2(fold change) < ‐2. For the GO term extracellular vesicles, a slight overexpression is observed under hydrodynamic conditions, with 13% (149 proteins) enriched in HydroCell versus 10% (117 proteins) enriched in Starv2D; and 19% (215 proteins) enriched in HydroSph versus 6% (68 proteins) in Starv3D. Wound healing and angiogenesis exhibited marginal enrichment under hydrodynamic conditions compared to starvation conditions. For wound healing, 6% (10 proteins) versus 15% (24 proteins) were enriched in HydroCell versus Starv2D; and 12% (18 proteins) versus 9% (13 proteins) were enriched in HydroSph versus Starv3D. For angiogenesis, 10% (12 proteins) versus 18% (23 proteins) were enriched in HydroCell versus Starv2D; and 12% (15 proteins) versus 9% (11 proteins) were enriched in HydroSph versus Starv3D. This demonstrates that for the cellular component GO term related to EV identification, as well as for the two molecular function GO terms (wound healing and angiogenesis), the EVs produced under hydrodynamic conditions exhibit a protein composition that is similar to those produced under starvation conditions. In contrast, a distinct mitochondrial signature was identified in hydrodynamic conditions, for both 2D and 3D comparisons: 32% (234 proteins) versus 0.6% (4 proteins) were enriched in HydroCell versus Starv2D; and 31% (221 proteins) versus 0.4% (3 proteins) were enriched in HydroSph versus Starv3D.

For the Z‐score heatmaps shown in Figure 3h–l, all four conditions were included, and proteins were selected with at least 2 distinct peptides across all 5 biological replicates of an experimental condition. For the GO terms wound healing (Figure 3j), angiogenesis (Figure 3k), and mitochondrion (Figure 3l), this narrowed the number of proteins to 36, 23, and 101, respectively. For wound healing and angiogenesis, as predicted by the volcano plots, the distribution of proteins was relatively uniform across all conditions. In contrast, for mitochondria‐associated proteins, the Z‐score was significantly higher for most proteins in the hydrodynamic stimulation conditions, again consistent with the overexpression observed in the volcano plots. For the GO term extracellular vesicles, the heatmaps were restricted to proteins whose joined presence defines EVs as outlined in the MISEV2023 guidelines.^[^ ^77^ ^]^ Of the 60 proposed transmembrane proteins (MISEV category 1, Figure 3h) and 47 cytosolic proteins (MISEV category 2, Figure 3i), 42 and 28 were respectively detected across the different conditions of the proteome analysis. The co‐presence of EV‐specific transmembrane and cytosolic markers indicates the presence of intact vesicles, as recommended by the MISEV guidelines. These proteomic analyses are consistent with Western blot results (Figure 2c), showing the presence of the EV‐specific transmembrane proteins CD63 and CD81, as well as the EV‐enriched cytosolic protein Syntenin‐1, across all conditions. They are also in agreement with the MACSPlex analysis (Figure 2d,e), which confirmed the co‐expression of CD63 and CD81 on the same EVs in all conditions.

The comprehensive proteomic analysis of EVs produced under hydrodynamic stimulation, from both individual cells and spheroids, confirmed the presence of 42 transmembrane and 28 cytosolic markers defined by the MISEV guidelines as core EV proteins (Figure 3h,i), validating the EV identity of the particles generated in this system. This was reinforced by GO analysis across all conditions showing no significant differences in protein content for apoptosis‐related terms (Figure S15, Supporting Information), indicating that apoptotic bodies are not part of the analyzed EV fractions despite the reported decrease in cell viability under hydrodynamic conditions (Figure 1). Furthermore, enrichment analysis of proteins not typically found in EVs showed similar or lower expression profiles in hydrodynamic stimulation conditions compared to starvation‐derived EVs (Figure S16, Supporting Information). However, the endoplasmic reticulum protein Calnexin and the Golgi apparatus protein Golgin subfamily A member 2 were found to be moderately enriched for these conditions.

To explore potential differences in EV biogenesis, we compared proteomic profiles of EVs produced under hydrodynamic stimulation and under standard serum starvation, both in monolayer and spheroid cultures. EV formation is typically attributed to two pathways: i) the endosomal route, producing exosomes via multivesicular bodies; and ii) direct plasma membrane budding, producing ectosomes. Both may occur through ESCRT‐dependent or ESCRT‐independent mechanisms. Our data revealed a consistent depletion of ESCRT components in EVs produced under hydrodynamic stimulation (Figure 3m), including all major ESCRT complexes and ALIX–syntenin‐syndecan pathway proteins (ALIX, SDCBP, SDC2/4), with the exception of EPS15 and CHMP6. This suggests that shear‐induced EVs are not primarily formed through canonical ESCRT‐dependent pathways. In contrast, hydrodynamic EVs were enriched in membrane‐associated proteins such as caveolins (CAV1, CAV2), flotillins (FLOT1, FLOT2), and several tubulin isoforms (Figure 3i). This enrichment points toward ESCRT‐independent mechanisms, possibly involving lipid microdomains and cytoskeletal remodeling, as the main drivers of EV biogenesis under hydrodynamic stimulation.

Overall, these proteomics data reveal that hydrodynamic stimulation induces a distinct proteome signature in the EVs produced, characterized by the enrichment of microvesicle/ectosome‐related proteins, and primarily formed through an ESCRT‐independent pathway. Therefore, the hydrodynamic stimulation not only enhances EV production but also induces a distinctive proteomic profile on the produced EVs, setting them apart from those produced under static conditions. The mitochondrial signature, highlighted by the enrichment of mitochondrial‐associated proteins, suggests a role in the energy metabolism. Such a tailored proteomic signature could be advantageous for specific therapeutic applications where enhanced metabolic activity or mitochondrial function is desired. Furthermore, this approach underscores the versatility and precision of hydrodynamic stimulation in modulating the content of EVs for the development of specialized EV‐based therapies.

### 2.3 Hydrodynamic Stimulation Increases EVs Potency

**Figure 4:**
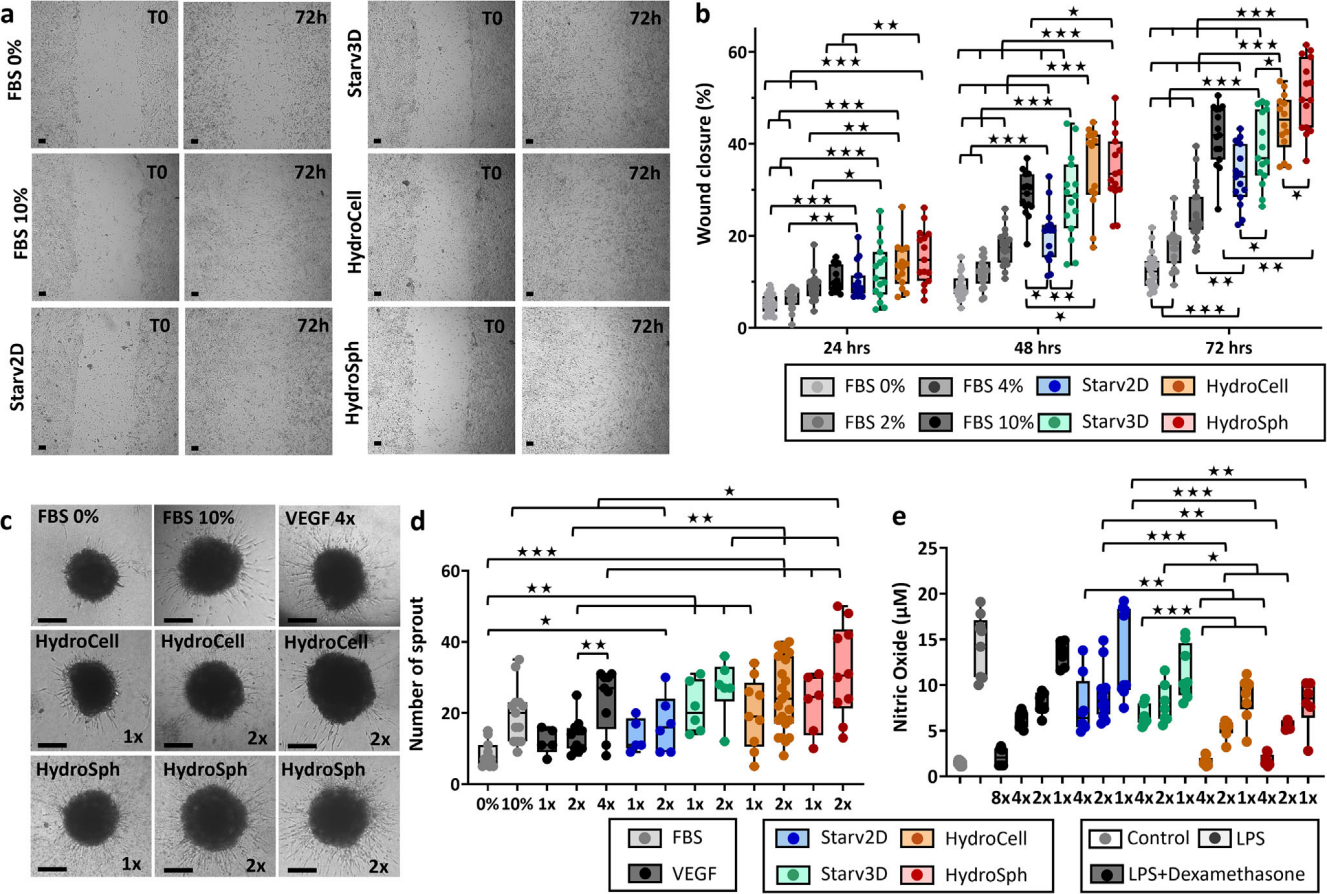
Functionality tests. EVs produced by static starvation in monolayers (Starv2D) or spheroids (Starv3D), as well as EVs produced by hydrodynamic stimulation in baffled tubes in alternate rotation from either individual cells (HydroCell) or spheroids (HydroSph), were tested for wound healing (a‐b), neo‐angiogenesis (c‐d), and anti‐inflammatory activity (e). a) Representative images depicting the initial wound gap (left) and wound healing at the 72‐h time point (right) are provided for all conditions. Scale bars = 100 µm. Positive controls for this test are serum conditions with different volume fractions (1%, 2% 4% and 10%), and the negative control corresponds to medium alone (no EVs, and 0% FBS). The administered dose (concentration measured with Videodrop) of EVs is 1 × 10^9^ EVs per mL (i.e., 10^8^ EV per wound). b) Time course of the effect of serum proteins or different EVs on wound healing over 3 days. c) Typical images of sprout formation from endothelial spheroids, illustrating the neo‐angiogenesis capacity. Scale bars = 100 µm. Various conditions were tested, including serum proteins (FBS) diluted to 10% (FBS 10%), no addition (FBS 0%), VEGF at three doses 12.5 (1x), 25 (2x), 50 (4x) ng mL^−1^, and EVs from the conditions Starv2D, Starv3D, HydroCell, and HydroSph (1x = 5 × 10^7^ EVs, equivalent to a concentration of 10^9^ EVs per mL). d) Number of sprouts measured for all conditions. e) Anti‐inflammatory activity of EVs produced from all conditions on human macrophages (RAW264.7) treated with lipopolysaccharide (LPS). LPS induced the formation of nitric oxide (NO), indicating an inflammatory reaction. Dexamethasone at different doses (1x = 1 µg mL^−1^) and EVs from all conditions (1x = 7.5 × 10^7^ EVs, equivalent to a concentration of 5 × 10^8^ EVs per mL), also at different doses, were administered. NO production was measured, and a reduction in NO levels reflected an anti‐inflammatory effect in a dose‐dependent manner. Significance was evaluated using an unpaired two‐tailed Student's *t*‐test, with p‐values categorized as follows: *p* > 0.05 (not significant), *p* < 0.05 (significant, ^*^), *p* < 0.01 (very significant, ^**^), and *p* < 0.001 (highly significant, ^***^). In panel (b), all conditions were compared pairwise at each time point, with the absence of a star indicating no significant differences between them. In panel (d), pairwise comparisons were also performed but limited to EV conditions with equivalent doses (1x and 2x), where the absence of a star signifies no significant differences. In panel (e), EV conditions were compared pairwise for identical doses (1x, 2x, or 4x), with comparisons to dexamethasone and LPS‐only controls provided in Table S1 (Supporting Information) (complete data could not be displayed on the graph). For instance, HydroSph EVs at a 4x dose exhibited greater efficacy than dexamethasone at doses 1x, 2x, and 4x, with high significance (^***^).

The next step was to test the EVs functional capacity for wound healing,^[^ ^79^ ^]^ angiogenesis^[^ ^20^ ^]^ and anti‐inflammatory activity. The ability of EVs to induce fibroblast migration, or wound healing capacity, was measured with a scratch test on human dermal fibroblasts (HDFs) seeded in 96‐well plates and upon which an artificial 1 mm gap wound was created at the center of the cell growth area. After the wound was created, EVs or serum alone were administered and wound closure was monitored over time by live imaging at 24, 48, and 72 h (**Figure** **4**a, other images in Figure S17, Supporting Information). For the Starv2D, Starv3D, HydroCell and HydroSph conditions the EV dose was set at 10^9^ particles per mL, equivalent to 10^8^ EVs per wound. These conditions were compared to medium alone and medium supplemented with fetal bovine serum (FBS) at concentrations of 2%, 4%, and 10%. The quantification of wound gap closure over 3 days for all the tested conditions is shown in Figure 4b. Naturally, increased FBS percentage gradually increased wound closure capacity. At all time points, the four EV conditions exhibited significantly higher reparative potential compared to FBS0% and FBS2%. The Starv2D condition yielded results similar to FBS4% but lower than FBS10% at 24 and 72 h. The other three EV conditions produced outcomes comparable to FBS10% at all time points, with a non‐significant trend toward improved wound healing, except for the HydroSph condition at 72 h, which showed a significant increase. Overall, EVs produced from 3D spheroids, either by starvation or by hydrodynamic stimulation, performed better in improving wound colonization compared to FBS at 4%, were similar to FBS at 10%, and were even more effective at 72 h for the hydrodynamic stimulation. Hydrodynamically stimulated EVs thus significantly enhanced the wound healing process, surpassing the effect of serum proteins up to 4%, exhibiting a comparable effect to serum proteins at 10% and demonstrating slightly greater efficacy than EVs produced using the classical method of starvation.

The neo‐angiogenic potential of EVs was next evaluated using human umbilical vein endothelial cells (HUVECs) cultured as 3D spheroids in agarose microwells and incorporated into a collagen matrix (Figure 3a). Similar conditions to those used for the wound healing assay were applied, testing only FBS0% (negative control) and FBS10% (positive control), with an additional positive control of VEGF administration at increasing doses (12.5, 25, and 50 ng mL^−1^). Treatment of these spheroids with VEGF or serum proteins (FBS) induced sprout formation (Figure 4c, additional images in Figure S18, Supporting Information). Quantification of the number of sprouts is shown in Figure 4d, and Figure S19 (Supporting Information) presents the sprout lengths. The administration of 5 × 10^7^ (10^9^ per mL) and 10^8^ EVs (2 × 10^9^ per mL) per endothelial spheroid gradually increased the number of sprouts per spheroid across all EV conditions, indicating their significant role in promoting angiogenesis.

Finally, the anti‐inflammatory potential of EVs was evaluated using RAW264.7 macrophages treated with lipopolysaccharide (LPS) to induce nitric oxide (NO) production, an indicator of inflammation (Figure 4e). All conditions showed an overall dose‐dependent reduction in NO formation. At the highest dose tested (4x = 3 × 10^8^ EVs), hydrodynamically produced EVs nearly completely suppressed NO production, achieving an effect comparable to that of dexamethasone 8 µg mL^−1^ (8x), thereby showcasing their potent anti‐inflammatory properties. We hypothesize that this notable anti‐inflammatory effect is associated with the overexpression of mitochondria‐related proteins in the hydrodynamically produced EVs. LPS‐induced inflammation in macrophages is known to result in mitochondrial damage and compromised mitochondrial activity and integrity.^[^ ^80^ ^]^ Therefore, restoring mitochondrial integrity and promoting biogenesis are crucial for mitigating and reversing inflammation, as well as preventing excessive responses to LPS stimulation.^[^ ^81^ ^]^ Emerging studies have shown that EV‐mediated transfer of mitochondrial components such as mitochondrial DNA (mtDNA) and proteins^[^ ^82^ ^]^ can enhance mitochondrial integrity and function in recipient cells, thereby reducing inflammation^[^ ^83^ ^]^ and promoting an anti‐inflammatory phenotype in LPS‐stimulated macrophages.^[^ ^81^ ^]^ Indeed, the mitochondrial transcription factor A (TFAM), which plays a role in mtDNA transcription and limits inflammation^[^ ^84^ ^]^ is significantly upregulated in EVs produced under hydrodynamic conditions (Figure 3l), which supports the observed anti‐inflammatory effects.

Thus, we propose that the observed anti‐inflammatory effect of hydrodynamically produced EVs is due to their ability to restore mitochondrial integrity by delivering mitochondria‐related proteins to LPS‐stimulated macrophages. This mechanism likely underpins the significant reduction in NO production and the overall anti‐inflammatory activity observed in our study.

Collectively, these results illustrate that applying hydrodynamic stimulation to producer cells within our system not only enhances EV production and increases yield but also results in EVs with functional properties in wound healing, neo‐angiogenesis and anti‐inflammatory activity.

### 2.4 Toward an All‐In‐One Approach: In Situ Production of Spheroids in Rotating Tubes, Followed by EV Production

**Figure 5:**
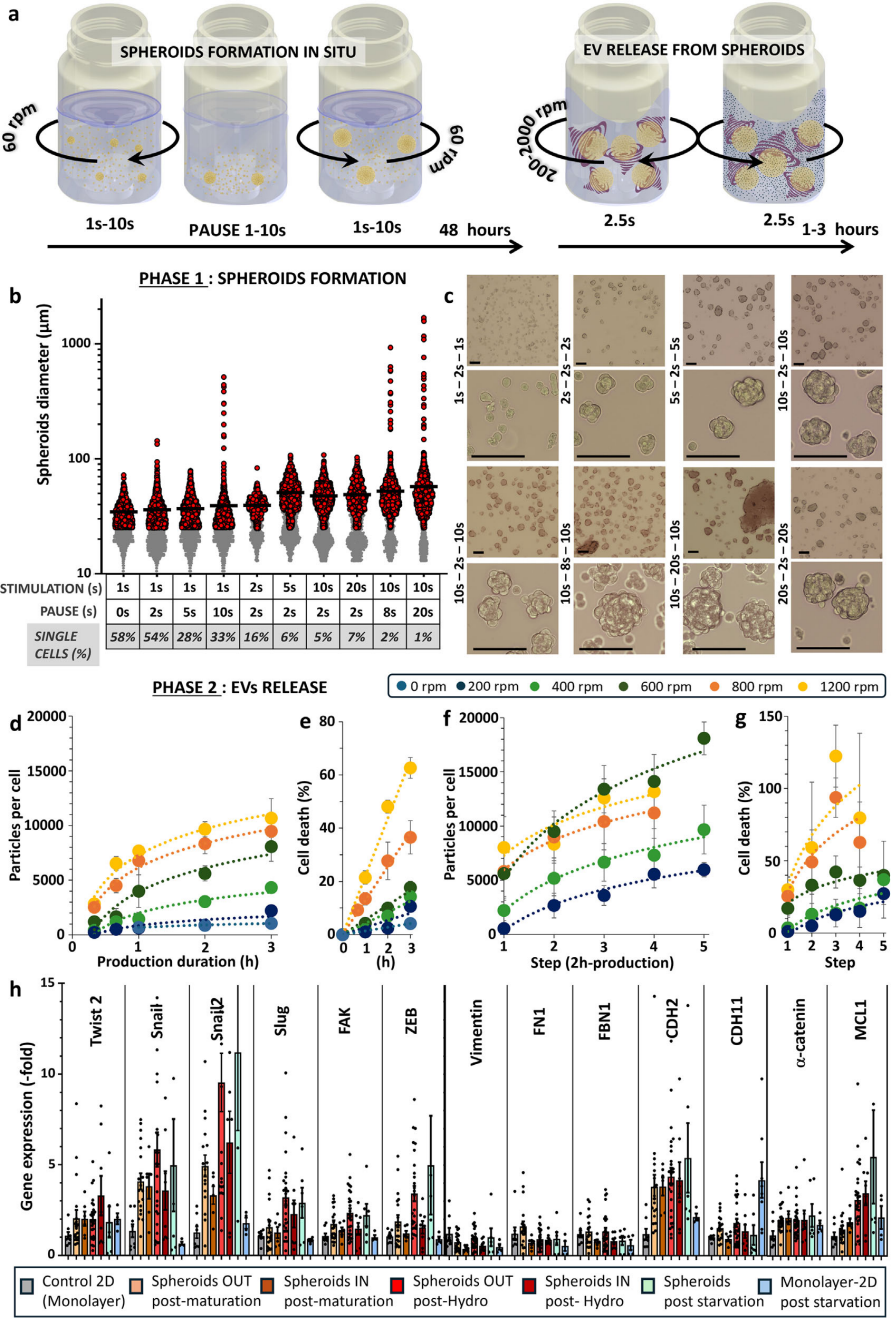
Streamlined bioproduction: Spheroid formation and EV release via tuning of stimulation parameters. a) Schematic diagram illustrating the all‐in‐one production approach. The process begins with single cells, progresses through their aggregation into spheroids, and culminates in the release of EVs. b) Diameter distribution of the produced spheroids (at least 200 spheroids measured for each condition), achieved using specific configurations of period of stimulation and pause between each direction change, as displayed in the accompanying chart. The speed of rotation was set at 60 rpm, with an acceleration of 120 rpm s^−1^. c) Representative images of spheroids produced under each condition, corresponding to the configurations shown in b. Scale bars = 100 µm. d) Quantification of EV production, as measured by Videodrop and presented as the number of particles produced per cell under different stimulation conditions (varied rpm) and durations (up to 3 h). e) Induced cell mortality under the same conditions as (d), as measured by ToxiLight assay. f) EV production with a stepwise protocol of 2‐h production periods interspersed with either 6 or 12 h slow rotation (non‐production phases), showing the number of particles produced per cell for different stimulations. g) Cell mortality associated with the conditions described in (f). h) Expression of a panel of mesenchymal and apoptotic genes measured by RT‐qPCR for control cells cultured in monolayers (Control 2D), after the formation of spheroids either in microwells (Spheroids Out post‐maturation) or in baffled tubes (Spheroids In post‐maturation), and after stimulations: hydrodynamic for spheroids Out and In (Spheroids Out post‐hydro & Spheroids In post‐hydro), and starvation in monolayers (Starv2D).

One of the main advantages of the technology is its complete tunability. The rotation speeds can reach values as high as 2000 rpm, with accelerations between 60 and 6000 rpm per second. The frequency of direction change is also fully customizable, with pauses between each rotation direction change also being possible. For EV production, we found that the optimal rotation speed was 600‐800 rpm, with a quick direction change recommended (5‐s period, or 2.5 s rotation in each direction). We then hypothesized that at slower rotation speeds coupled with pauses, the tube could be used upstream for spheroid formation, leading to an all‐in‐one approach of in situ spheroid formation and direct EV production from them. This approach is illustrated by the schematic in **Figure** **5**a. For the spheroid formation phase, the rotation speed was set at 60 rpm (1 rotation per second) with an acceleration of 120 rpm s^−1^ and direction change periods ranging from 2 to 40 s, and a pause between each direction change ranging from 0 to 20 s. Starting with individual cells suspended in the tubes, the resulting spheroid diameters were analyzed using image processing. Figure 5b displays the distribution of the obtained diameters, where all diameters below 15 µm correspond to individual cells (indicating no spheroid formation), with the corresponding percentage also indicated in the figure. Images of the obtained spheroids are shown in Figure 5c, with additional images for the same rotation parameters presented in Figure S20 (Supporting Information). The best conditions identified involved short 2‐s pauses and rotation periods of 10–20 s; in these conditions, almost no cells remained individual (less than 6%), and we avoided overly large structures exceeding 100 µm in diameter. For the subsequent phase, the maturation protocol selected was 10 s of rotation in one direction, a 2‐s pause, and then 10 s of rotation in the reverse direction.

Next, for the EV production phase, the quantification of the number of EVs per producing cell is presented in the graphs of Figure 5d,e. These graphs correspond to the same conditions as Figure 1i,j, where EVs were produced from spheroids prepared outside the tubes (HydroSph) in microwells, and hydrodynamically stimulated once for times between 1–3 h. The production rates (Figure 5d) and spheroid viability (Figure 5e) are very similar for spheroids prepared in situ in the tubes (HydroSphIn), with optimal production in terms of yield versus viability ≈600 rpm. We then sought to exploit the advantage of having the spheroids in situ in the tube, which opens up the possibility to alternate between production phases and rest/maturation phases for the spheroids, i.e., multiple hydrodynamic stimulations on the same spheroids. The results of this approach are shown in Figure 5f (production) and Figure 5g (viability), where 2‐h production periods alternate with rest periods (spheroid maturation protocol) of either 6 or 12 h, allowing for the collection of a small volume fraction for particle quantification in the morning, late afternoon and the following day. At 600 rpm, the highest production rates are achieved, with 15 000 EVs per producing cell (as measured by Videodrop, equivalent to more than 60 000 EVs per cell by the standard NanoSight measurement), a rate rarely, if ever, matched by other production methods. However, mortality increases after the second stimulation but remains below 40%, while it can reach up to 80% for 1600 rpm stimulations, which are also lower in terms of production. Although producing fewer EVs, slower rotating conditions of 200 and 400 rpm are especially interesting for more fragile cells, with significant production rates exceeding 5000 EVs per cell and with a lower mortality rate.

Clearly, the production of EVs from spheroids formed in situ presents a significant advantage in the hydrodynamic bioproduction technology. However, it remains necessary to evaluate the impact of the spheroid formation on their biology. To this end, we analyzed the gene expression profiles of hMSC spheroids formed either in microwells or directly in situ (Figure 5h). These analyses were conducted both after spheroid formation (before stimulation) and following the stimulations, either hydrodynamic or static starvation. Various mesenchymal, epithelial and anti‐apoptotic markers were assessed using RT‐qPCR. Overall, these markers were present in all spheroid conditions and were consistently over‐expressed compared to the 2D monolayer control, indicating a retained mesenchymal signature regardless of formation mode or stimulation applied. Comparing spheroids formed in microwells to those formed directly in situ revealed no significant differences, suggesting that the formation process does not impact spheroid maturation; in situ‐formed spheroids are comparable to those formed externally.

Hydrodynamic stimulation slightly increased the expression of most mesenchymal markers, as well as the anti‐apoptotic marker MCL1. This increase was also observed post‐starvation, albeit to a higher degree, especially for the MCL1 marker. These findings suggest that stimulations do not drastically alter the genomic signature of the spheroids but may have a slight impact on their biology. Notably, the impact of hydrodynamic stimulation appears less significant compared to starvation, indicating that hydrodynamic stimulation better preserves the biological integrity of the spheroids than traditional starvation methods used for EV production.

### 2.5 EVs Produced from Spheroids Formed In Situ Maintain Excellent Functionality

**Figure 6:**
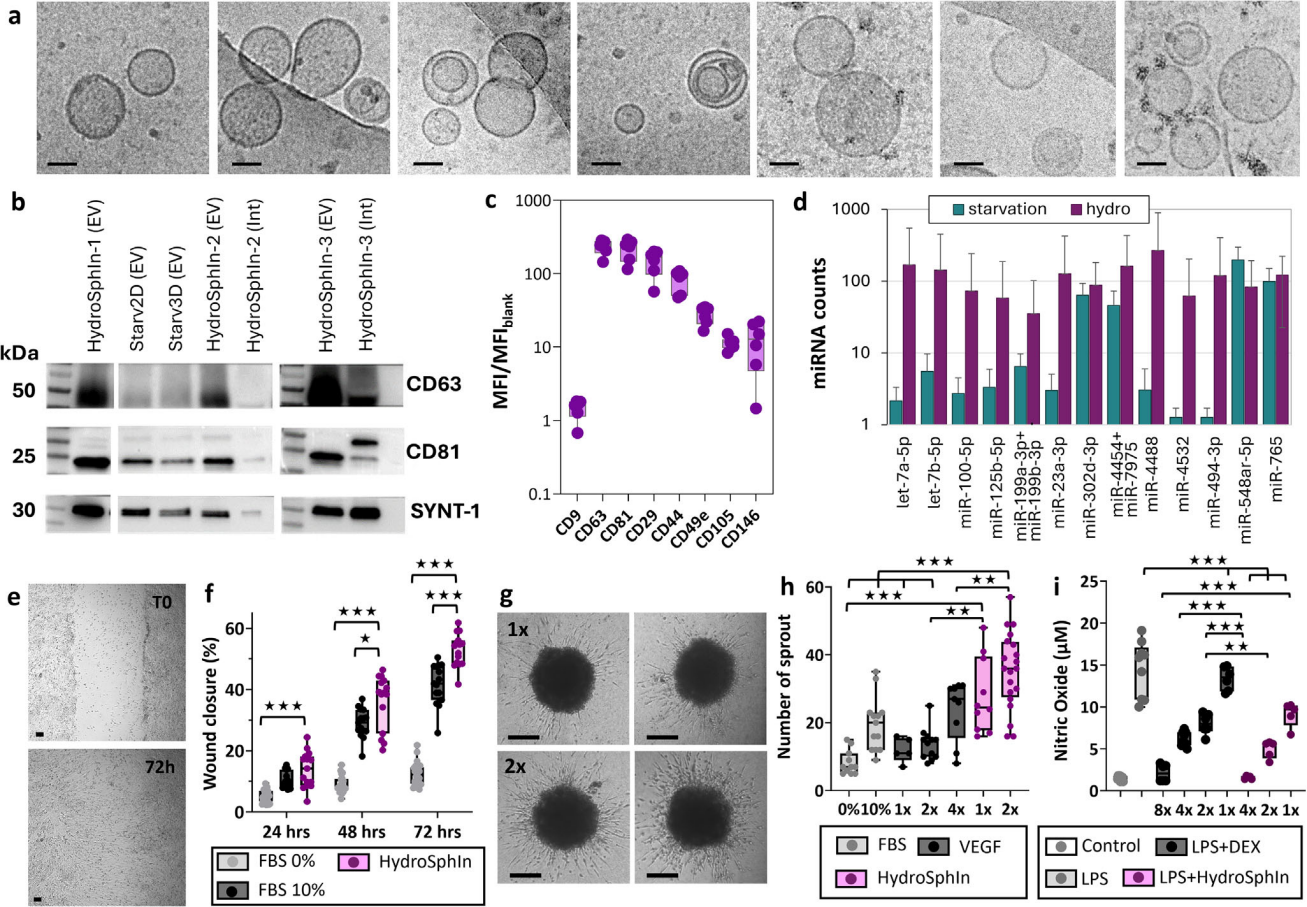
Characterization of EVs produced hydrodynamically from in‐tube formed spheroids (HydroSphIn). a) Morphology of EVs observed by CryoTEM. Scale bars = 50 nm. b) Western blots for the proteins CD63, CD81, and Synt‐1, for EV fractions from control conditions Starv2D and Starv3D, and three independent HydroSphIn productions (HydroSphIn‐1, ‐2, and ‐3). Intermediate fractions (Int) post SEC (see Methods) for conditions ‐2 and ‐3 are also shown. c) MacsPlex quantification, similar to Figures 2d,e, for 5 independent HydroSphIn production conditions. d) Average expression levels of EV‐associated microRNAs for EVs produced under starvation and hydrodynamic conditions (see Methods for production details). Bar graphs show the mean NanoString counts (± SEM) for each miRNA across six independent biological replicates per condition. Only microRNAs with an average normalized count above 30 in at least one of the two conditions are displayed. EVs were profiled using the nCounter® Human v3 miRNA Expression Assay. e) Representative wound healing images for a HydroSphIn condition at the initial wound and after 72 h. Scale bars = 100 µm. f) Quantification of wound healing effect, similar to the graph presented in Figure 3b. Controls (FBS 0% and 10%) are the same, with purple points indicating independent measurements for HydroSphIn. Dose was set at 10^9^ EVs per mL, or 10^8^ EVs per wound. g) Images of neo‐angiogenesis for two doses of HydroSphIn EVs. Scale bars = 100 µm. h) Quantification of the number of sprouts, with the same FBS and VEGF controls as in Figure 3c,d. EV dose = 5 × 10^7^ (10^9^ per mL, 1x) and 10^8^ EVs (2 × 10^9^ per mL, 2x). Purple points represent independent measurements for HydroSphIn. i) Anti‐inflammatory effect of HydroSphIn EVs (independent purple points, 1x = 7.5 × 10^7^ EVs, equivalent to a concentration of 5 × 10^8^ EVs per mL), with the same FBS, LPS, and LPS+DEX controls as in Figure 3e. Significance was assessed using an unpaired two‐tailed Student's t‐test, with p‐values interpreted as follows: *p* > 0.05 (not significant), *p* < 0.05 (significant, ^*^), *p* < 0.01 (very significant, ^**^), and *p* < 0.001 (highly significant, ^***^).

The standout feature of this system is its capacity to streamline both spheroid production and their large‐scale release of EVs. Consequently, it was crucial to evaluate the biological properties and therapeutic potential of these EVs produced by spheroids formed in situ (SphIn). **Figure** **6**a shows the cryo‐TEM morphological analysis for the condition HydroSphIn, revealing structurally intact EVs with similar characteristics as those observed previously (additional images are provided in Figure S12, Supporting Information).

The expression of CD63, CD81, and Syntenin‐1 (SYNT‐1) was confirmed by Western blot (Figure 6b), and the co‐expression of CD63 and CD81 on the same vesicles was further validated using MACSplex EV analysis (Figure 6c). Mesenchymal markers‐CD29, CD44, CD49e, CD105, and CD146‐were also detected. The expression profile of these markers in the HydroSphIn condition was comparable to that observed under conventional starvation‐based production (Starv2D and Starv3D), as well as in the HydroSph and HydroCell conditions. All expression levels across conditions are presented in the same graph in Figure S21 (Supporting Information). A preliminary exploratory analysis was conducted to assess the microRNA (miRNA) cargo of EVs produced under hydrodynamic stimulation compared to those obtained under classical starvation conditions. This approach follows recent recommendations to characterize not only the protein but also the RNA content of EVs, particularly miRNAs, which are increasingly recognized as important effectors of EV‐mediated biological activity. In the Nanostring EV miRNA panel analyzed, microRNAs with average counts above 30 in at least one condition are shown in Figure 6d. Many of these miRNAs have recognized roles in regenerative medicine: for example, the let‐7 family (let‐7a‐5p, let‐7b‐5p) promotes neural and bone regeneration^[^ ^85^ ^]^ and inhibits fibrosis; miR‐199a‐3p/199b‐3p support cardiac repair, kidney protection and tissue remodeling; miR‐100‐5p aids bone formation, neuroprotection and muscle differentiation; miR‐125b‐5p/1285‐5p regulate stem cell differentiation, fibrosis and apoptosis; miR‐23a‐3p enhances proliferation, inhibits apoptosis and modulates inflammation^[^ ^86^ ^]^; miR‐302d‐3p drives stem cell pluripotency and reprogramming and represses inflammation^[^ ^87^ ^]^; and miR‐494‐3p promotes angiogenesis^[^ ^88^ ^]^ and tissue repair. Less characterized miR‐4454/7975 modulate immune responses and inflammation, while emerging miRNAs (miR‐4488, 4532, 548ar‐5p, 765) likely influence proliferation, apoptosis, and extracellular matrix remodeling. These miRNAs are transported within the EVs with their abundance influenced by the production conditions. Notably, higher levels of most analyzed miRNAs were observed in EVs generated under hydrodynamic conditions, potentially contributing to enhanced therapeutic efficacy in regenerative medicine applications.

For the functional assays, Figure 6e displays a representative image of wound closure induced by SphIn‐derived EVs, showing significant efficacy (with additional images in Figure S17, Supporting Information), and Figure 6f quantifies the healing kinetics, revealing comparable effectiveness to 10% FBS at 24 h, and statistically superior healing at 48 and 72 h. Figure 6g demonstrates sprouting from endothelial cell spheroids in response to SphIn‐derived EVs, with the effect quantified in Figure 6h, showing superior efficacy to VEGF and FBS. Lastly, Figure 6i highlights the robust anti‐inflammatory activity of EVs produced from the spheroids formed in situ under hydrodynamic conditions.

### 2.6 Hydrodynamic Stimulation of Stem Cell Spheroids: Game Changer in EV Manufacturing?

The demand for reliable and scalable EV production, isolation and characterization methods continues to grow, driven by ever‐increasing advances in EV research across scientific and biotechnological fields.^[^ ^89^ ^]^ The lack of consensus on optimal manufacturing technologies for therapeutic vectors^[^ ^30^ ^]^ remains a significant barrier to the development of EVs. In response, this study introduces a novel approach that leverages hydrodynamic stress to subject spheroids to turbulent flow and stimulate them to release EVs at a remarkably high rate. By finely tuning key production parameters, this methodology addresses the need for efficient and robust EV manufacturing processes. In the present study, only one size of the rotating tube bioreactor was tested. However, the system is inherently scalable—both through scale‐out (increasing the number of units) and scale‐up (increasing the size of the bioreactor), the latter being a technically feasible option. Notably, this process integrates stem cell suspension culture, spontaneous spheroid formation, and hydrodynamic stimulation for EV release into a single streamlined workflow. This not only simplifies operations but also enables EV production within a biomimetic environment, preserving key mesenchymal and anti‐apoptotic markers essential for spheroid integrity.

Beyond increasing EV yield, this hydrodynamic approach reveals a proteomic profile that aligns with the standards defined by the MISEV guidelines,^[^ ^77^ ^]^ featuring a combination of transmembrane and cytosolic EV proteins. It also highlights the presence of proteins involved in wound healing or neo‐angiogenesis, correlating with the demonstrated wound healing and angiogenic properties of the produced EVs. Most importantly, they possessed a unique enriched mitochondria‐associated protein signature. In fields such as cardiovascular therapeutics, the ability of the technology to produce mitochondria proteins‐rich EVs is particularly noteworthy. For instance, mitochondria‐enriched EVs have demonstrated the capacity to improve cardiac function following ischemia in preclinical studies.^[^ ^90^ ^]^

From a pharmaceutical perspective, the hydrodynamic flow parameters can be identified as critical process parameters (CPP), as they significantly influence the production process. Monitoring and controlling these parameters align with regulatory frameworks such as ICH Q8 (R2) to ensure the quality of the final product. This relationship between process parameters and the critical quality attributes (CQAs) of EVs enhances the development of precise design spaces in pharmaceutical manufacturing. Additionally, the lack of robust, reproducible, and scalable production methods remains a barrier to the clinical and commercial translation of EV‐based therapies. The technology proposed here could offer a solution to bridge the gap between academic research and industrial applications. Thanks to its process tunability and potential alignment with regulatory requirements, it positions itself as a strong candidate for clinical and commercial adoption. The recent patenting of this methodology for EV bioproduction (publication number WO2024/200570) underscores its novelty and potential impact. Furthermore, it extends to other healthcare fields, as evidenced by additional patents filed for the bioproduction of bacterial probiotics (EP24306529.9) and lentiviruses (EP24306529.9).

## 3 Conclusion

EV production through hydrodynamic stimulation using the alternating rotation baffled tube technology falls within the high‐yield range, comparable to the most efficient existing methods. However, the protocol enables rapid EV production only within short timeframes (on the order of hours), as prolonged exposure to intense hydrodynamic stress compromises cell viability. To address this limitation, EV production can be extended over longer durations by incorporating recovery phases between stimulation steps, which provides the rationale for our choice of a stepwise rather than continuous production approach. Hydrodynamically produced EVs retain their essential characteristics, including morphology, size and protein cargo consistent with MISEV 2018 classes 1 and 2. Proteomic analysis confirmed the presence of EV‐associated proteins and identified clusters of proteins linked to wound healing and angiogenesis, correlating with functional benefits such as enhanced sprout formation in endothelial cells and accelerated wound closure in dermal fibroblasts. Mitochondria‐associated proteins were notably upregulated under hydrodynamic conditions, likely driving the superior anti‐inflammatory activity observed in hydrodynamically produced EVs.

Remarkably, and thanks to its tunable simulation parameters, the rotating tube technology not only supports EV production but also facilitates in situ spheroid formation. These spheroids retain mesenchymal genetic profiles comparable to those formed outside the tubes, with minimal alterations following hydrodynamic stimulation. Similarly, the EVs produced by spheroids formed in‐tubes exhibit characteristics and functionalities akin to those from pre‐formed spheroids. This dual functionality —combining spheroid formation and EV release within a single platform— offers a competitive solution for developing tailored EV production processes that meet specific therapeutic requirements.

With its capacity for both scale‐out and scale‐up to enable production in the liter range, this technology is well‐positioned to transform high‐yield EV bioproduction and facilitate its translation into clinical and industrial settings. The system design is compatible with GMP‐compliant workflows and can be readily adapted to incorporate single‐use tubing and chambers, medical‐grade and certified components, as well as closed and sterile operation throughout scale‐up. Such features make this platform particularly suited for integration into standardized and regulatory‐compliant EV manufacturing pipelines.

Lastly, although the widely‐used SEC‐based method yields EVs of high analytical quality, the possible presence of soluble or co‐eluting proteins remains a limitation that may restrict its applicability in both research and translational settings. Emerging lipid‐based purification technologies offer promising complementary approaches for achieving higher‐grade and ultra‐pure EV preparations.^[^ ^94^, ^95^ ^]^

## 4 Experimental Section

### Cell Lines and Cell Culture

The study utilized hTERT‐immortalized human Mesenchymal Stem Cells (hMSC; abm, T0523, RRID: CVCL_VG68), hTERT‐immortalized Human Skin Fibroblasts (HSF; abm, T0326, RRID: CVCL_4Y34), Human Umbilical Vein Endothelial Cells (HUVEC; ATCC, CRL1730, RRID: CVCL_2959), and RAW 264.7 macrophage (Sigma, 91062702‐1VL, RRID: CVCL_0493). All cell lines were cultured in a humidified atmosphere of 5% CO_2_ at 37 °C (incubator MCO‐201IC, Panasonic), and all culture media were supplemented with 1% penicillin‐streptomycin (Gibco, 15140‐122). Specifically:

hMSCs: Cultured in T150 flasks (TPP, 90150) with culture medium Prigrow II (abm, TM002) supplemented with 10% Fetal Bovine Serum (FBS) (Gibco, 12662029), and 1 µM hydrocortisone (Sigma, H0135). It should be noted, however, that to advance beyond proof of concept toward biopharmaceutical production, serum‐free and xeno‐free culture media alternatives will need to be implemented.

HSFs: Cultured in PriCoat T25 flasks (abm, G299) in culture medium Prigrow III (abm, TM003) supplemented with 10% FBS (Gibco, A5209402).

RAW 264.7 and HUVECs: Cultured in T75 flasks (TPP, 90 075) in DMEM (Gibco, 21 885) supplemented with 10% heat‐inactivated FBS (Dutscher, S1900‐500C).

All cell lines were routinely screened for mycoplasma contamination on a monthly basis. All cells used in this study were confirmed to be mycoplasma‐free.

### Spheroids Formation in Microwells

Spheroids were formed in microwells using custom 3D‐printed circular molds. Each mold featured 2269 pillars, each 200 µm in height and diameter, and was designed to fit into the wells of a 6‐well plate. These molds were created with a DigitalWax 028J Plus 3D printer and DigitalWax DS3000 resin. To prepare the microwells, a solution of 2% agarose (Sigma, A0576) in PBS was heated and poured into the wells of a 6‐well plate. The 3D‐printed molds were carefully placed in contact with the agarose solution, and the assembly was cooled at 4 °C for 20 min to allow the agarose to solidify. After gelation, the molds were removed, and the plates were sterilized under UV light for 1 h and stored at 4 °C in PBS until use. For spheroid formation, hMSCs (passages 10‐20) were detached from their culture flasks, centrifuged at 300 g for 5 min, and resuspended at a concentration of 700 000 cells mL^−1^ in complete medium. 1 mL of this cell suspension was added to each well containing the agarose microwells. The plates were incubated at 37 °C for 30 min to allow cells to sediment into the microwells, followed by the addition of 2 mL of complete medium per well. The cells were then incubated at 37 °C for 24 h to form cohesive spheroids.

Prior to use, spheroids were recovered from the microwells by gentle pipetting, followed by three washing steps. Each wash involved centrifugation at 200 g for 3 min and resuspension in 220‐nm filtered DMEM without serum or phenol red (Gibco, 31053‐028). Particle concentrations in this filtered medium were systematically measured, consistently found to be at very low levels (often below detection limits on the Videodrop NTA, or in the range of 10^6^–10^7^ particles per mL; and ≈ 10^7^ particles per mL on the NanoSight NTA).

Regarding spheroid density in the production experiments, the same density within each independent production series (biological replicate, using the same cell stock) were consistently used. However, the initial number of MSCs per volume varied, especially in early experiments exploring different stimulation conditions to optimize yield, leading to variations in cell concentration per mL. Nevertheless, the number of cells per spheroid (≈ 300 cells) was always kept constant.

### EVs Production under Starvation Conditions from Monolayers and Spheroids

*From monolayers*: To produce EVs from monolayers (starvation 2D), cells were grown in T150 flasks (TPP, 90150) until they reached confluency (≈ 10 million cells per flask, equivalent to 500 000 cells per mL). The culture medium was then replaced with 20 mL of fresh serum‐free medium, and the flasks were incubated at 37 °C with 5% CO_2_ for 72 h. After this incubation period, the conditioned medium containing EVs was collected and centrifuged at 2000 g for 10 min at 4 °C for further processing.

*From spheroids*: For the production of EVs from spheroids (starvation 3D), T150 flasks were pre‐coated with 20 g L^−1^ agarose diluted in PBS to prevent cell adhesion. A suspension of spheroids was prepared as previously described, with a density ranging between 500 and 2000 spheroids per mL (≈ 150 000 to 600 000 cells per mL). Each flask was filled with 20 mL of this spheroid suspension and incubated at 37 °C with 5% CO_2_ for 72 h. Following the incubation, the medium containing spheroids and EVs was collected and centrifuged at 300 g for 5 min to remove the spheroids. The resulting conditioned medium was then further centrifuged at 2000 g for 10 min at 4 °C for subsequent use.

### Reverse Rotating Baffled Tube Device

A prototype of the device was developed to optimize an all‐in‐one approach for the technology. This initial prototype incorporates 3D‐printed tubes, motors, electronics, an incubator for thermal and CO_2_ regulation, and a parameter control program. A photograph of the prototype is available in Figure S22 (Supporting Information).

*3D printing of the tubes*: The tubes were printed using a DigitalWax 028J Plus 3D printer with LOCTITE 3D MED412 resin, which meets ISO 10993‐5 and ‐10 standards for biocompatibility.

*Motors*: Stepper motors (RS PRO, 2.8 V, 5 mm shaft diameter) were used for this prototype. These motors are easily controllable, robust, and cost‐effective. They were mounted on an aluminum breadboard (250 mm × 250 mm, Thorlabs), with six motors per board.

*Electronics and Programming*: The motor microcontrollers were attached to PCBs to optimize space and wiring. A Raspberry Pi microcomputer was used to generate the control interface, and the interface software was coded in Rust.

*Incubator*: One disadvantage of the stepper motors used in this prototype is the heat they generate when powered. To ensure proper regulation of various parameters (temperature and gas), the aluminum breadboard with the motors was placed inside a small Peltier‐cooled CO_2_ incubator (WCI‐40P model, INSTRUMAT), with chamber dimensions of 320 × 350 × 375 mm.

*Reynolds number calculation*: To characterize the hydrodynamic environment within the reverse rotating baffled tube, it can calculate the baffle‐associated Reynolds number (Re). It is a relevant global descriptor of the flow, based on the assumption that the shear stresses relevant to EV secretion are primarily generated by fluid perturbations induced by the baffles. In this case, it must consider as the characteristic velocity the mean fluid velocity in the case of rigid body rotation, which corresponds to v = (2/3)^*^v_wall_, where v_wall_ = ωR is the tangential velocity at the tube wall. This estimate reflects the average momentum transport across the cross‐section of the fluid and avoids overestimating inertial effects based solely on the wall velocity. Re is therefore defined as:

(1) $$Re_{\mathrm{baffle}}=\frac{\rho \overset{⇀}{\upsilon }\cdot L_{\mathrm{baffle}}}{\mu }$$

where ρ is the fluid density, µ is the dynamic viscosity of water, v is the estimated mean velocity, and L_baffle_ = 0.01 m is the radial size of the baffle.

*Particle tracking experiment*: To visualize particle motion under hydrodynamic stress, the baffled tube was filled with water to eliminate air–liquid interfaces and bubble formation. The tube was sealed with a custom glass cap to allow top‐view imaging. Spherical glass beads of 200 µm diameter, selected to mimic the size and density of biological spheroids, were suspended in the fluid. Their motion was recorded using a high‐speed camera (Phantom VEO E310L) operating at 1000 frames per second.

Raw videos were first registered using a custom MATLAB script to correct for background motion or drift. The resulting image stacks were then post‐processed in ImageJ to enhance contrast. Particle tracking was performed using the TrackMate plugin in ImageJ, applying the Laplacian of Gaussian (LoG) detector followed by the simple Linear Assignment Problem (LAP) tracker. Manual corrections of tracking errors and link breaks were performed using the TrackScheme graphical tool within TrackMate to ensure continuity and accuracy of trajectories. Particle instantaneous velocities were then derived and smoothed (to improve visualization and reduce measurement artifacts) using a custom MATLAB script. The same script was used to plot the particle trajectories, color‐coded by smoothed velocity.

### Spheroids Formation and Hydrodynamic EV Production in Reverse Rotating Baffled Tubes

This novel device for EV production boasts complete tunability: it allows for adjustable rotation speeds (1–6000 rpm), inversion frequencies (0.01–10 Hz), and optional pauses (1–60 s). These parameters govern the establishment of internal hydrodynamic flows, enabling an all‐in‐one approach for different regimes, from cell suspension cultures and in situ spheroid formation to EV production from cells and spheroids.

*Spheroid formation in tubes*: Cells are suspended in their complete culture medium at a density ranging from 200 000 to 500 000 cells per mL. The stimulation parameters to be tested vary between 40 and 80 rpm for rotation speed, 0.05 to 1 Hz for inversion frequency, and 0 to 20 s for pauses between rotations. Under certain conditions, cells are cultured in suspension without forming spheroids.

*EV production in tubes*: For EV production, the producer cells, either as individual cells or spheroids (formed in microwells or directly in the tubes), are harvested and subjected to three washes with serum‐free DMEM to remove any serum residues that could interfere with particle measurement. Cells were then resuspended in 20–25 mL per tube at densities ranging from 200 000 to 500 000 cells per mL (≈ 500 to 2000 spheroids per mL) for the initial series of experiments. However, for all final productions with SEC purification, the density was standardized at 500 000 cells per mL (≈ 1500 spheroids per mL). The reverse rotation protocol is initiated (200‐1600 rpm, 0.2 Hz, no pause), typically for a maximum of 3 h, with optional pauses (up to 20 s each) to collect 100 µL samples for particle measurement. Sequential stimulation is also possible, alternating rapid phases (200–1200 rpm, 0.2 Hz) for 2 h with maturation phases (60 rpm, 0.05 Hz, 2‐s pauses) for 6‐12 h. At the end of the production process, the tube volume is collected, spheroids or cells are removed by centrifugation at 300 g for 5 min, and the conditioned medium is prepared by centrifugation at 2000 g for 10 min at 4 °C for further processing.

### Viability Assays

*Live/Dead imaging of spheroids*: 100 µL of Live/Dead reagent (Invitrogen 488/570) were added to 50 µL of spheroids suspension (containing between 100 and 1000 spheroids) in 96‐well plate (PerkinElmer, Optiplate‐96) and left to incubate for 30 min at room temperature. Spheroids were imaged with a plate reader (Ensight Multimode Plate Reader, PerkinElmer) in bright field and in fluorescence mode (excitation 465 nm and 535 for Live (green) and Dead (orange) respectively. Fluorescence intensity was quantified using Fiji on regions of interest (ROIs) defined by the spheroid contours. Briefly, the ROI was standardized to a circular area with a 50 µm diameter (≈ 2000 µm^2^). Fluorescence images were thresholded, and ROIs were centered on all spheroids identified using the *Analyze Particles* function, considering only areas above 2000 µm^2^. Fluorescence intensity was then measured, generating distributions of Live and Dead signals across several thousand spheroids.

*Toxilight cell death assay*: 20 µL of Toxilight reagent (Lonza) were combined with 5 µL of the conditioned media in 384‐well plate (PerkinElmer, Optiplate‐384) and left for 15 min at room temperature. Luminescence was next measured by plate reader (0.1 s measurement time). The positive control was generated by lysing spheroids or cells at the same concentration using the lysis buffer provided in the Toxilight kit, following the manufacturer's instructions.

*AlamarBlue metabolic activity assay*: Cells or spheroids were placed into a 96‐well plate, with a density of ≈ 200‐800 spheroids per well or 5000–20 000 cells per well. Control samples, corresponding to non‐stimulated cells or spheroids, were seeded at the same density in each experiment. Each well was then incubated with 200 µL of phenol red‐free DMEM supplemented with 10% Alamar Blue (Invitrogen) for 2 h. After incubation, 100 µL of the medium from each well was transferred to a new 96‐well plate. Fluorescence was measured using the Ensight Multimode Plate Reader, with an excitation wavelength of 570 nm and an emission detection wavelength of 585 nm.

### Nanoparticles Tracking Analysis: Videodrop Measurements

Interferometric Light Microscopy (ILM) using the Videodrop instrument (Myriade, Paris) was employed to analyze nanoparticle concentration and hydrodynamic diameter by recording the diffraction patterns created by nanoparticles moving in the light path. Prior to each measurement, the sample chip was thoroughly cleaned with ethanol and distilled water.

For the measurements, an 8 µL of sample were placed at the center of the sample chip. The chip was then positioned in the optical path of the Videodrop instrument. Samples were analyzed directly at their concentration in conditioned medium, with an effective working range of 10^8^–10^10^ particles per mL. At least duplicate measurements per samples were performed. The ILM signal enabled nanoparticle detection and tracking, with the data processed using the qvir software, with the doublet detector for all measurements. Measurements were also conducted at time zero, before production, to serve as a baseline.

### Nanoparticles Tracking Analysis (NTA): NanoSight Measurements

NTA was performed on conditioned medium samples using the NanoSight NS300 (Malvern) with a 532 nm laser module. Prior to analysis, if needed, samples were diluted to achieve a maximal concentration of 2 × 10^8^ particles mL^−1^. For each measurement, 1 mL of the diluted sample was injected into the apparatus chamber at a flow rate of 20–30 µL min^−1^ using a sterile 1 mL syringe. Baseline measurements, including buffer and time zero before production, were systematically performed. Each sample underwent five 1‐min acquisitions, captured with the camera level set to 16. The recorded videos were subsequently analyzed using NanoSight NTA software, maintaining a detection threshold of 4.

### Fluorescent Nanoparticle Tracking Analysis (f‐NTA)

For f‐NTA quantification, EVs were first enriched from conditioned medium (CM) by ultrafiltration using 100 kDa cut‐off centrifugal filters (Centricon Plus‐70, 100 kDa, Sigma, UFC710008), concentrating the samples ≈ 100‐fold. The concentrated EV suspensions were then incubated with fluorophore‐conjugated antibodies against the tetraspanins CD63 and CD81 (anti‐CD63‐520 and anti‐CD81‐520, Particle Metrix GmbH, Germany) for 1 h at room temperature in the dark, following the manufacturer's recommended volumes.

Fluorescent NTA was performed on the NanoSight NS300 instrument (532 nm laser). Measurements were acquired in both scatter (standard NTA) and fluorescence (f‐NTA) modes. The total particle count, as well as the concentrations of CD63⁺ and CD81⁺ particles, were determined from the respective modes. Particle concentrations were then back‐calculated to the initial cell number, accounting for the dilution and concentration factors introduced during filtration and antibody incubation, to express the results as the number of particles per producing cell.

### EV Isolation by Size Exclusion Chromatography (SEC)

For all productions purified by SEC, the production method has been standardized to using 1500 spheroids per mL, or equivalently, 500 000 cells per mL. To concentrate the conditioned media, 100 kDa cut‐off centrifugal filters (Sigma, Centricon Plus‐70 100 kDa, UFC710008) were utilized, yielding 200‐300 µL of concentrated conditioned media (CCM), in accordance with the manufacturer's instructions. EVs were subsequently isolated using SEC. For this process, SEC columns (Izon Sciences, qEVoriginal/70 nm Gen2 SEC columns, ICO‐70) were pre‐washed with 25.5 mL of PBS. Each sample's 500 µL of CCM was then carefully applied to the top of the columns. After the entire sample entered the column, PBS was added. The initial 2 mL fraction was discarded, and the following 2.4 mL fraction, containing the EVs, was collected. In certain cases, a subsequent 2.4 mL fraction (Intermediate fraction) was also collected. Both EV and Intermediate fractions were then re‐concentrated using 10 kDa cut‐off centrifugal filters (Milipore, Amicon Ultra‐4, UFC8010), resulting in 100 µL of concentrated EV suspension (EV pool). The EV pools were either placed at 4 °C for immediate analysis or stored at ‐80 °C.

### Cryogenic Transmission Electron Microscopy (cryoTEM) Imaging

A 5 µL sample aliquot was applied to a quantifoil carbon membrane grid (Quantifoil Micro Tools GmbH). Excess liquid was blotted from the grid for a few seconds, followed by rapid plunging into liquid ethane to form a thin vitreous ice film. The grid was then mounted onto a Gatan 626 cryo‐holder, cooled with liquid nitrogen, and inserted into a JEOL JEM2100 (LaB6) electron microscope operating at 200 kV. The temperature was maintained at ‐180 °C throughout the imaging process. Images were captured using an Ultrascan 1000 CCD camera (Gatan) at a resolution of 2k × 2k pixels.

### Western Blot Analysis

EVs samples were prepared for Western blot analysis by resuspending 30 µL of samples post SEC in 4x Laemmli Sample Buffer (Bio‐Rad). The samples were then boiled for 10 min at 95 °C and loaded onto 4–15% Mini‐Protean TGX Stain‐Free gels (Bio‐Rad) for electrophoretic separation under non‐reducing conditions. Following electrophoresis, proteins were transferred onto Immuno‐Blot PVDF membranes (Bio‐Rad) using a semi‐dry transfer method. After blocking, the membranes were incubated with primary anti‐human antibodies: CD81 (clone TS81, Medix Biochema 1/1000), CD63 (clone H5C6, BD Bioscience 557305 1/1000), SDCBP (syntenin‐1, clone EPR8102, Abcam 1/1000) and 14‐3‐3 (Clone EPR6380, Abcam, 1/1000). After primary antibody incubation, the membranes were post‐incubated with horseradish peroxidase (HRP)‐conjugated secondary antibodies goat anti‐rabbit IgG (H + L) (Jackson 111‐035‐144) and goat anti‐mouse IgG (H+L) (Jackson 111‐035‐146). After washing, immunoreactive bands were detected using Clarity western ECL substrate (Bio‐Rad) with an exposure time of 16.250 s. The results were visualized using ChemiDoc Touch imager (Bio‐Rad).

### Bead‐Based Multiplex Flow Cytometry Assay (MACSPlex)

EVs were analyzed using a bead‐based multiplex assay for flow cytometry (MACSPlex EV Kit IO, human, Miltenyi) following the manufacturer's protocol. Briefly, EV concentration was quantified by nanoparticle tracking analysis (NTA), and the particle count was used to estimate the input EV quantity. A total of 5×10^8^ EVs were diluted in MACSPlex buffer to achieve a final volume of 120 µL. Then, 15 µL of MACSPlex Exosome Capture Beads were added to the samples, which were incubated overnight at room temperature on an orbital shaker, shielded from light. After incubation, the samples were washed and then incubated with a mixture of APC‐conjugated anti‐CD9, anti‐CD81, and anti‐CD63 detection antibodies for 1 h at room temperature. Following this, flow cytometry analysis was performed using an Aurora analyzer (Cytek). Data were processed and analyzed using FlowJo software (v10, FlowJo LLC). For data analysis, the 39 individual bead populations corresponding to different EV markers were gated, allowing the determination of the APC signal intensity for each respective bead type. Median fluorescence intensity (MFI) for each capture bead population was measured, and background signals were corrected by dividing the MFI values by the signal from non‐EV controls, which underwent identical processing to the EV‐containing samples.

### Protein Quantification

The protein content of EVs was quantified utilizing the Micro BCA Protein Assay Kit (Thermo Fisher Scientific). To establish a standard curve, Bovine Serum Albumin was diluted to final concentrations of 0.5, 1, 2.5, 5, 10, 40, and 200 µg mL^−1^. In parallel, EVs were mixed with RIPA buffer (Thermo Fisher Scientific) at a 1:7 dilution and incubated on ice for 30 min for EV lysis. EVs lysates were then diluted in phosphate buffer saline (PBS) to reach a final 1:10 RIPA concentration. 45 µL of each EVs sample and each standard were then transferred in triplicates into a 384‐well microplate (PerkinElmer, Optiplate‐384) and 45 µL of the BCA working reagent was added to each well. The plate was then agitated on a plate shaker for 30 s, covered, and incubated at 37 °C for 2 h. Absorbance readings at 562 nm were taken using a Ensight plate reader, and protein concentrations were calculated based on the standard curve.

### Whole Proteome Analysis

Five biological replicates of each analyzed condition were assessed by Liquid Chromatography coupled with Tandem Mass Spectrometry (LC‐MS/MS) using 5 µg of proteins of each replicate.

*Sample Preparation for LC‐MS/MS*: Protein pellets of 5 µg each were dried under vacuum using a Savant Centrifuge SpeedVac concentrator (Thermo Fisher Scientific). The dried pellets were then solubilized and reduced in 10 µL of 8 M urea, 100 mM ammonium bicarbonate, and 5 mM dithiothreitol (DTT) at pH 8.0, and incubated at 57 °C for 1 h. Following this, the samples were allowed to cool to room temperature, after which iodoacetamide was added to a final concentration of 10 mM. The alkylation reaction was conducted in the dark for 30 min at room temperature. The samples were subsequently diluted to a final urea concentration of 1 M using 100 mM ammonium bicarbonate at pH 8.0. Protein digestion was performed using trypsin/LysC (1 µg, Promega) in a total volume of 100 µL, with the mixture incubated overnight at 37 °C under constant vortexing. To desalt the digested peptides, the samples were passed through homemade C18 StageTips. Elution of peptides was achieved using a solution of 40% acetonitrile (CH3CN) and 60% water containing 0.1% formic acid. The eluate was then concentrated to dryness under vacuum.

*LC‐MS/MS Analysis*: The peptide samples were analyzed using a Vanquish Neo LC system (Thermo Scientific) connected to an Orbitrap Astral mass spectrometer, utilizing a Nanospray Flex ion source (Thermo Scientific). Separation was achieved on a C18 column (75 µm inner diameter × 50 cm, 2 µm particle size, 100 Å pore size, Thermo Scientific) maintained at 50 °C. Peptides were eluted using a linear gradient of 100% buffer A (0.1% formic acid in water) to 28% buffer B (0.1% formic acid in acetonitrile) over 104 min at a flow rate of 300 nL min^−1^. Ionization was performed with a spray voltage of 2200 V, funnel RF level set to 40, and a heated capillary at 285 °C. Full MS scans were acquired in the range of 380–980 m/z with a resolution of 240 000 at m/z 200, using a normalized AGC target of 500% and a maximum injection time of 5 ms. Fragmentation was carried out in data‐independent acquisition (DIA) mode, covering a precursor mass range of 380–980 m/z with 2 Da isolation windows. Fragment ions were generated in the HCD cell with a normalized collision energy of 25%, a normalized AGC target of 500%, and a maximum injection time of 3 ms.

*Data Processing LC‐MS/MS*: Data analysis was conducted using the Pulsar search engine integrated with Spectronaut v19 (Biognosys) to perform directDIA+ analysis on the collected LC‐MS/MS data. The raw data were searched against the Homo sapiens (UP000005640) Uniprot database. The search parameters included trypsin enzyme specificity, allowing up to two missed cleavage sites. Carbamidomethylation of cysteine was specified as a fixed modification, while N‐terminal acetylation and methionine oxidation were considered variable modifications. The processed data were further analyzed using myProMS v3.10 (available at https://github.com/bioinfo‐pf‐curie/myproms).^[^ ^91^ ^]^ Protein quantification was performed by extracting ion chromatograms (XICs) of proteotypic peptides common to the conditions being compared (TopN matching). The analysis permitted missed cleavages and included carbamidomethylation. Peptide‐level median and scale normalization was applied to adjust the total signal across the five biological replicates. To assess the significance of protein abundance changes, a linear model adjusted for peptides and biological replicates was employed. A two‐sided T‐test was conducted on the fold change estimates derived from the model, and p‐values were corrected using the Benjamini‐Hochberg FDR method. Label‐Free Quantification (LFQ) was also performed according to a specified algorithm,^[^ ^92^ ^]^ requiring a minimum of two peptide ratios and utilizing the large ratios stabilization feature. Proteins were considered significant if they were identified with at least two distinct peptides across five biological replicates and exhibited a log2(fold change) ≥ 2 or ≤ ‐2 with an adjusted p‐value < 0.05. Proteins with a log2(fold change) between ‐2 and 2 were deemed common between the conditions. The raw mass spectrometry proteomics data can be found in the ProteomeXchange Consortium via the PRIDE partner repository^[^ ^93^ ^]^ with the dataset identifier PXD059610.

### Transcriptomic Analysis with RT‐qPCR

hMSCs cells or spheroids were collected either post‐maturation (after spheroids formation and before stimulation), post‐rotation (after stimulation) or post‐starvation (no rotation stimulation). Total RNA of those samples was extracted using NucleoSpin RNA mini kit (Machery‐Nagel, Thermo Fisher Scientific, #740955.50) following the manufacturer's instructions. To avoid contaminations with genomic DNA, RNA samples were incubated with desoxyribonuclease (DNAse) allowing this unwanted genomic DNA degradation. The extracted RNA concentrations and purities were then assessed using the Nanodrop device (Ozyme).

Reverse transcription was performed from 1 µg of the previously extracted RNA using the High‐Capacity cDNA Reverse Transcription kit (Thermo Fisher Scientific, #4368814) with random primers, according to the manufacturer's protocol. The obtained cDNA was then quantified using the same Nanodrop device.

Real‐time quantitative PCR was carried out, starting with 30 ng of cDNA using Power SYBRGreen qPCR Master Mix (Thermo Fisher Scientific, #A25742), on the QuantStudio 3 instrument (Applied Biosystems). Expression of the 60S acidic ribosomal protein P0 (RPLP0) was used as reference gene to normalize the obtained Ct values of the target genes. Next, the normalized Ct values were compared to the control 2D monolayer and gene expression folds were calculated based on the comparative Ct method: $2^{-(\Delta Ct_{condition}-\;\Delta Ct_{control}\;)}$. Mesenchymal markers genes (Twist2, Snail1&2, Slug, FAK, ZEB, Vimentin, FN1, FBN1, CDH2, CDH11), epithelial marker gene (α‐catenin) and anti‐apoptotic‐associated gene (MCL1) were assessed by RT‐qPCR. The primer sequences are listed in **Table** **1**.

**Table 1:** Primer sequences used for real‐time quantitative PCR.

Gene name	Forward primer (5′ to 3′)	Reverse Primer (5′ to 3′)
**Twist2**	AGC GAC GAG ATG GAC AAT AAG ATG ACC	CGG TCC GGA GGT GGG TGG CG
**Snail1**	TCC TTC GTC CTT CTC CTC TAC TT	TGT TGC AGT ATT TGC AGT TGA AG
**Snail2**	TGC CCT CAA GAT GCA CAT CCG A	GGG ACA GGA GAA GGG CTT CTC
**Slug**	AGA TGC ATA TTC GGA CCC ACA	CCT CAT GTT TGT GCA GGA GAG
**FAK**	ATC CCA CAC ATC TTG CTG ACT T	GCA TTC CTT TTC TGT CCT TGT C
**ZEB**	TTC ACA GTG GAG AGA AGC CA	GCC TGG TGA TGC TGA AAG AG
**Vimentin**	TAC AGG AAG CTG CTG GAA GG	ACC AGA GGG AGT GAA TCC AG
**FN1**	AGC GGA CCT ACC TAG GCA AT	ACA GCT TAT TCT CCC TCG CC
**FBN1**	GCT CCC AAA CCC TGC AAT TT	GGC AGT TGT GTT GCT TGG TTG
**CDH2**	AGC CAA CCT TAA CTG AGG AGT	GGC AAG TTG ATT GGA GGG ATG
**CDH11**	TGG CAG CAA GTA TCC AAT GG	TTT GGT TAC GTG GTA GGC AC
**α‐catenin**	GCG AAG GAG AGC CAG TTT CT	ATG TTG CCT CGC TTC ACA GA
**MCL1**	CAG CGA CGG CGT AAC AAA C	ACA AAC CCA TCC CAG CCT CTT T

### Wound Healing Assay

Human skin fibroblasts (HSFs) were seeded into 96‐well plates (TPP, 92096) at a density of 100 000 cells per well and cultured at 37 °C with 5% CO2 until they reached confluence. A physical wound was created within the monolayer using a 3D‐printed template and a sterile 200 µL pipette tip. The culture medium was then replaced with 100 µL of serum‐free medium, or serum‐free medium supplemented with 1%, 2%, 4%, or 10% FBS as well as serum‐free medium containing EVs produced under various conditions. These EVs originate from independent batches (biological replicates, n>3), as used for the proteomic analyses. To ensure measurement reliability, and when sufficient material was available, three replicates (three independent wounds) were tested. The initial EV concentration was set at 10^9^ EVs mL^−1^. HSFs were incubated for 96 h at 37 °C, 5% CO_2_, with images taken periodically using a PerkinElmer Ensight Multimode Plate Reader. At each time point, images of the wound area were acquired using a plate reader (Ensight Multimode Plate Reader, PerkinElmer) under identical exposure settings to ensure consistency across measurements.

Wound closure progression was quantified using Fiji (ImageJ, NIH), following standardized thresholding and segmentation procedures adapted from commonly used in vitro scratch assay protocols. Brightfield images were acquired at consistent exposure settings using a plate reader (Ensight Multimode Plate Reader, PerkinElmer), ensuring uniform illumination and minimizing external light variability. To quantify cell migration into the wound area, a binary mask was created by applying a global threshold, which was first calibrated on intact monolayer regions far from the scratch to accurately detect cell‐covered areas. The same threshold was then applied to the wound region. Manual segmentation was used to delineate the borders of the wound gap. Figure S23 (Supporting Information), provides representative illustrations of the thresholding procedure. To ensure consistency across samples, a central region of interest (ROI) measuring 0.6 mm in width, oriented perpendicularly to the wound axis, was defined within the wound area for all analyses. Only cell coverage within this defined ROI was quantified. The wound closure was expressed as the percentage of the ROI area occupied by cells. This approach allowed for the systematic and reproducible analysis of cell migration dynamics across multiple wells and time points.

### Angiogenesis Sprouting Assay on Collagen Matrix

HUVEC spheroids were generated using a protocol similar to that for hMSC spheroids, with the use of agarose microwells. However, in this instance, the spheroids were produced individually in a 96‐well plate, with each well containing a single micropillar mold with a diameter of 500 µm. Thus, a total of 96 microwells were created in each well of a 96‐well plate. Following the formation of microwells using 20 mg mL^−1^ agarose (200 µL of agarose poured per well) and sterilization, 10 000 HUVECs were added in 20 µL on top of each microwell. The plate was incubated at 37 °C for 30 min, then centrifuged at 200 g for 3 min, and 100 µL of medium was added. The cells were incubated for 3 days to allow for spheroid maturation. A 2 mg mL^−1^ collagen type I solution (Corning) was prepared on ice, and 30 µL was added to each well of a 384‐well plate. After allowing the collagen to polymerize for 30 min at 37 °C, HUVEC spheroids were carefully transferred onto the collagen matrix, with one spheroid per well. The plates were kept at room temperature for 20 min to allow the spheroids to settle. Subsequently, the culture medium was replaced with 50 µL of one of the following treatments: VEGF‐α (Sigma) at concentrations of 12.5 (1x), 25 (2x), or 50 (4x) ng/mL; EV pools produced under various conditions and diluted in DMEM, with two doses of 10^9^ (1x) EVs and 2 × 10^9^ (2x) EVs per mL; These EVs correspond to biological replicates (n>3), produced under fully independent culture and production conditions, most of which match the samples used for proteomic analysis. However, to ensure measurement reliability—and when sufficient EV quantities were available—the analysis was performed in triplicates, using three independent spheroids. DMEM supplemented with 10% serum or DMEM alone as a control. The plates were incubated for 24 h at 37 °C and subsequently imaged using the PerkinElmer Ensight Multimode Plate Reader in bright‐field mode. Angiogenic sprouting was quantified using Fiji software by measuring both the number and length of sprouts emerging from each HUVEC spheroid. Images were first thresholded and binarized to detect all cellular structures. The spheroid core was then manually excluded from the binary mask to isolate only the sprouting extensions. Sprout length was calculated using the *Analyze Particles* function, which provides a curvilinear measurement of each individual sprout. The same analysis also yielded the total number of sprouts per spheroid.

### Anti‐Inflammatory In Vitro Assays

To assess the anti‐inflammatory activity of EVs, mouse macrophage cells (RAW 264.7) were cultured in DMEM supplemented with 10% FBS (Gibco 10270‐106), 1% penicillin‐streptomycin (Gibco 15140‐122) at 37 °C, 5% CO_2_ and kept at low passage (< P10) and low confluency. Stimulation of macrophages with LPS induces the formation of nitric oxide (NO), an indicator of inflammatory reaction. For the assay, cells were seeded in a 96‐well plate at a density of 25 000 cells per well in DMEM supplemented with 5% FBS and 1% penicillin‐streptomycin. After 48 h, culture medium was removed and cells were stimulated with 150 µL of DMEM supplemented with 5% FBS and antibiotics (negative control), completed medium supplemented with LPS 0.5 µg mL^−1^ only (positive control), with LPS 0.5 µg mL^−1^ and EVs (1x = 7.5 × 10^7^ EVs, 2x = 1.5 × 10^8^ EVs, 4x = 3 × 10^8^ EVs) or with LPS 0.5 µg mL^−1^ and dexamethasone (Sigma, D4902)(1x = 1 µg mL^−1^) (inhibition control) for 24 h. At the end of the incubation time, an equal volume of a 2% sulphanilamide in 10% phosphoric acid solution and a 0.2% naphtylethylenediamine in water solution were mixed to obtain the Griess’ Reagent. 50 µL of culture medium supernatant was then mixed with 50 µL the Griess’ reagent and incubated 10 min in the dark at room temperature. To measure the NO production, absorbances at 550 nm (measured wavelength) and 620 nm (reference wavelength) were immediately measured using a plate reader EnSight (Perkin Elmer). Sodium Nitrite (NaNO_2_) was used in different concentrations to build a standard curve.

### miRNA Profiling using NanoString

EVs were produced under two conditions: nutrient starvation (72 h) or hydrodynamic stimulation (600 rpm for 2 h), using six independent biological replicates per condition, each starting from ≈ 50 million cells. In both cases, cells were incubated in serum‐free medium. After production, conditioned media were collected and centrifuged at 2000 × g for 10 min at 4 °C to remove cells, spheroids, and debris. Supernatants were concentrated using Centricon Plus‐70 100 kDa filters (2000 × g, ≥30 min), yielding 200–300 µL of concentrated conditioned medium. This volume was adjusted to 500 µL and loaded onto a size‐exclusion chromatography (SEC) qEVoriginal column. Fractions between 2 and 4.4 mL, corresponding to EV‐rich eluates, were pooled and reconcentrated using 10 kDa Amicon Ultra‐4 centrifugal filters to obtain the final EV preparations.

Total RNA was extracted from the EV samples using the miRNeasy Micro Kit (Qiagen) and analyzed with the nCounter Human v3 miRNA Expression Assay (NanoString Technologies). Mature miRNAs were first ligated to unique sequence‐specific tags, followed by hybridization with complementary reporter and capture probes. These complexes were then digitally quantified without amplification. Data normalization was performed using internal controls and condition‐specific housekeeping RNAs (corresponding to either the “Hydro” or “Starvation” group). Due to observed differences in housekeeping RNA counts between the two conditions, separate normalizations were applied to each dataset. Only miRNAs with average normalized counts exceeding 30 in at least one condition were retained for downstream analysis.

### Statistical Analysis

If applicable, statistical differences between groups were assessed using an unpaired, two‐tailed Student's t‐test. P‐values were denoted as follows: *p* > 0.05 (not significant NS), *p* < 0.05 (significant ^*^), *p* < 0.01 (significant ^**^), and *p* < 0.001 (significant ^**^).

## Conflict of Interest

The authors declare no conflict of interest.

## Supporting information



Supporting Information

## Data Availability

The data that support the findings of this study are available in the supplementary material of this article.
