# Supplementary material for: A Hydrodynamic Bioreactor for High‐Yield Production of Extracellular Vesicles from Stem Cell Spheroids with Defined Cargo Profiling
Source: Adv Sci (Weinh). 2025 Nov 19;13(10):e10607. doi: 10.1002/advs.202510607 (PMC12915174; doi:10.1002/advs.202510607)
Supplement: Supplementary file 1 — Supporting Information [file ADVS-13-e10607-s001.pdf]

Supporting Information

A hydrodynamic bioreactor for high-yield production of extracellular vesicles from stem cell spheroids with defined cargo profiling

*Solène Lenoir, Elliot Thouvenot, Giacomo Gropplero, Léonie Dec, Damarys Loew, Clotilde Théry, Jose E Perez, and Claire Wilhelm**

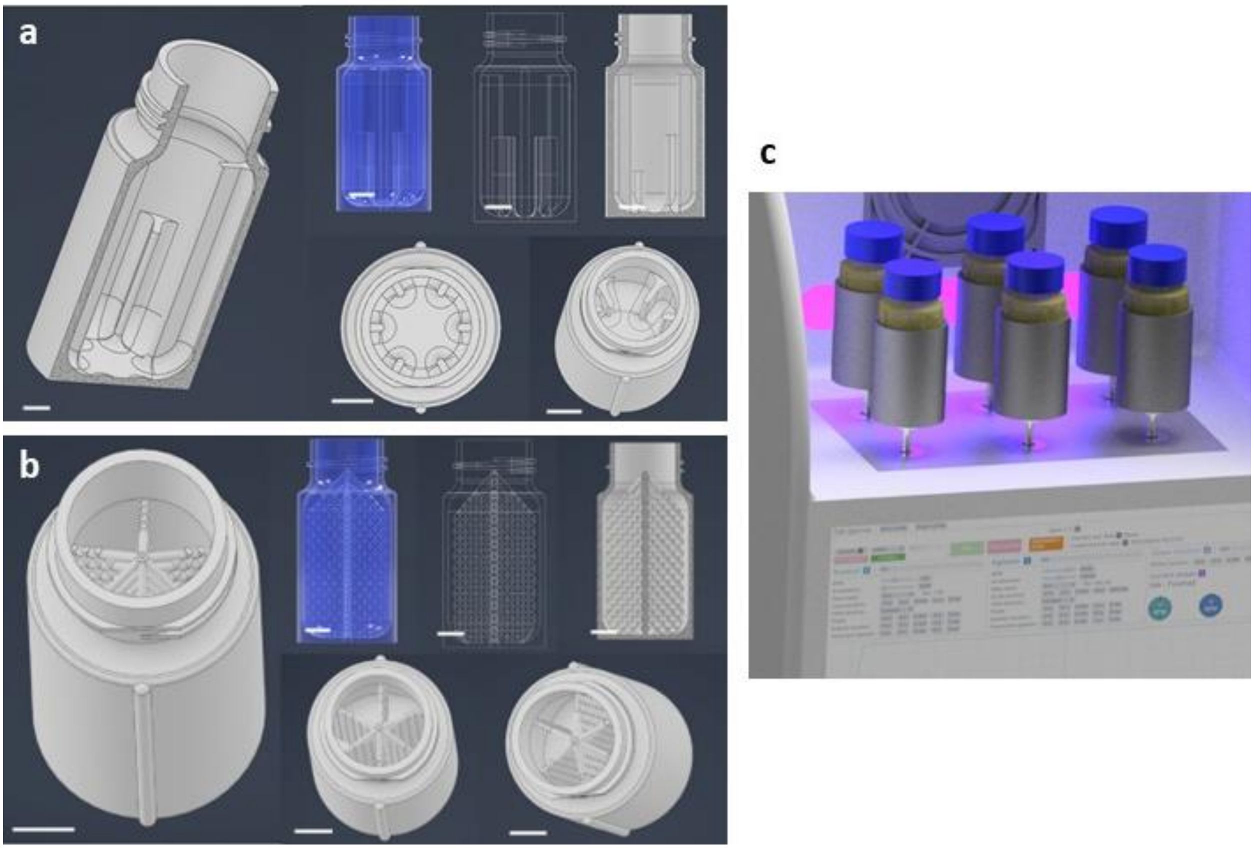

Figure S1 : (**a-b**) Conceptual sketches of the baffled designs. Scale bars = 1 cm. (**a**) Configuration used in the present study, featuring internal cut walls; (**b**) An alternative design initially tested, incorporating internal grid-like structures. While this second configuration yielded functional results (see output in Figure S2), it was discarded due to its incompatibility with injection molding, which is the only scalable manufacturing method suitable for integration into a bioproduction pipeline.
(**c**) Diagram illustrating the strategy used to rotate the tubes. Here, six motors enable control over rotation, including direction reversal, with rotation speed, acceleration, and inversion frequency all managed via software written in Rust and interfaced with the prototype through a Raspberry Pi. A photograph of the prototype is shown in Figure S20, associated to its description in the Methods section.

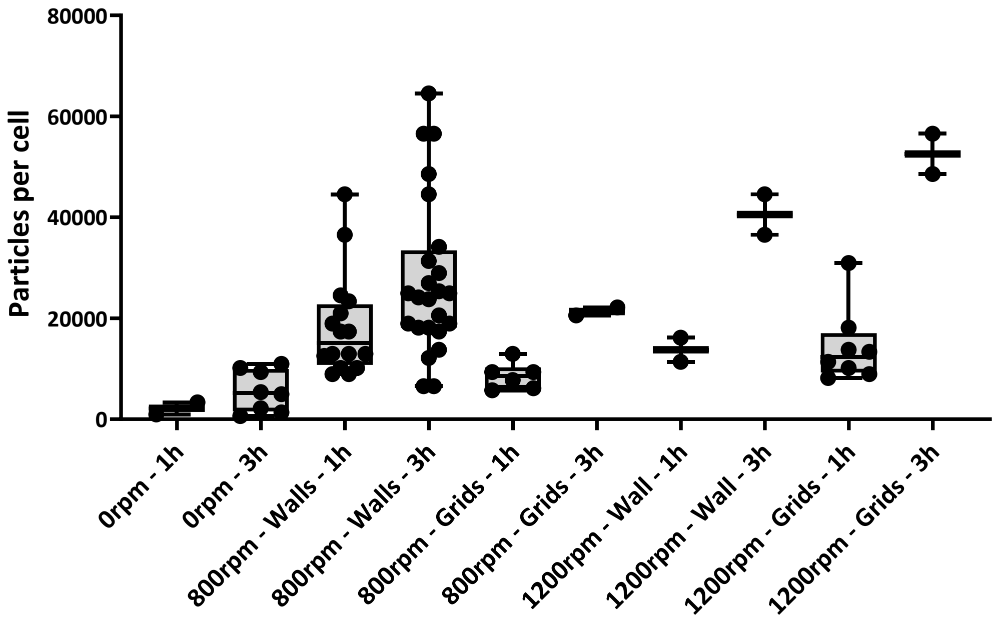

Figure S2: Comparison of EV production in baffled tubes with internal walls and with grid-like geometries, from spheroids produced in agarose and stimulated at 800 and 1200 rpm. Both tube geometries have a similar efficiency yield, in the range of 30,000 and 50,000 particles per producer cell (forming the spheroids), for 3 hours stimulation at 800 and 1200 rpm, respectively, as measured with the NanoSight Nanoparticle Tracking Analysis method. Each data point corresponds to an independent production run, derived from a distinct batch of cells. These cells were first aggregated into spheroids, then placed into the tubes corresponding to different geometries, subjected to rotation at a given speed, and maintained in production conditions for either one hour or three hours.

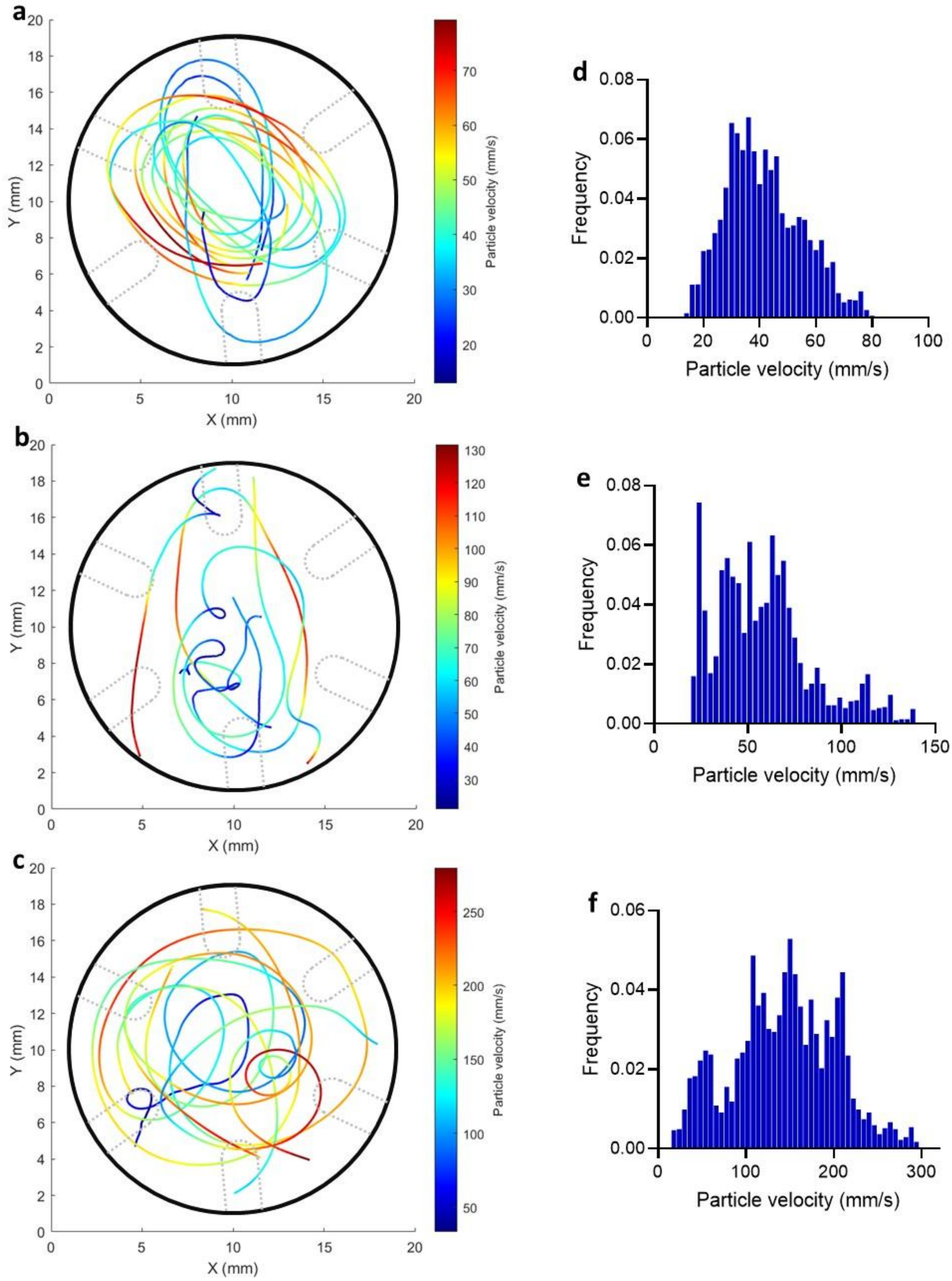

Figure S3: (a–c) Trajectories of selected particles in the system at 400 RPM **(a)**, 800 RPM **(b)**, and 1600 RPM **(c)**. The color scale indicates the smoothed instantaneous velocity along the trajectory. (d–f) Corresponding distributions of smoothed instantaneous velocities for all tracked particles at 400 RPM **(d)**, 800 RPM **(e)**, and 1600 RPM **(f)**.

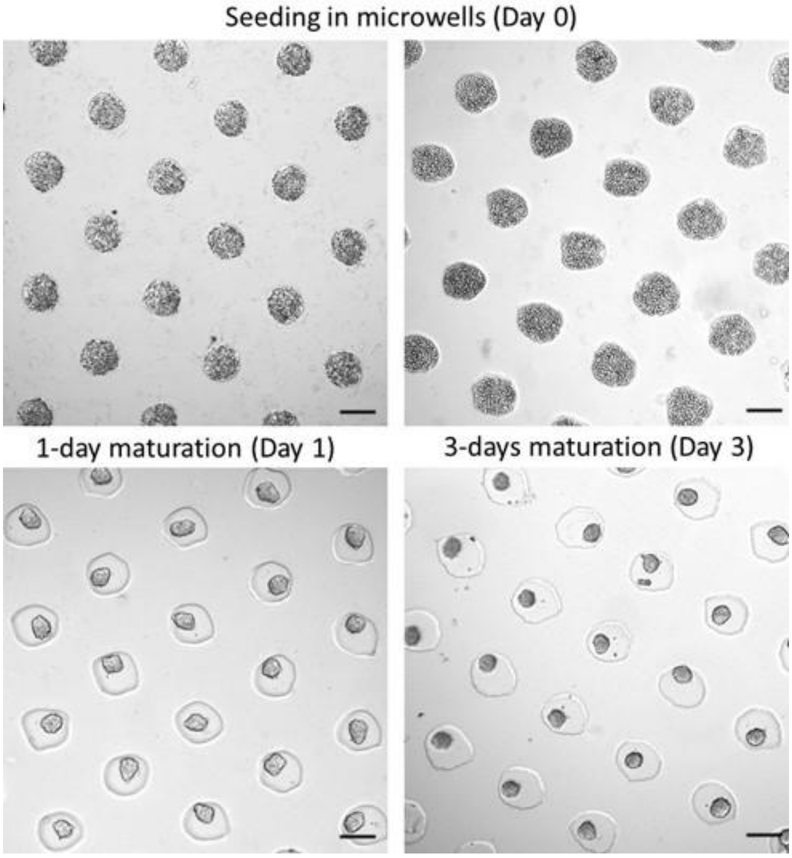

Figure S4: Production of spheroids in agarose microwells from human mesenchymal stem cells. Optical microscopy images show cell aggregation and growth from initial cell seeding (day 0) up to 3 days of maturation into a spheroid configuration. Scale bars = 200 µm.

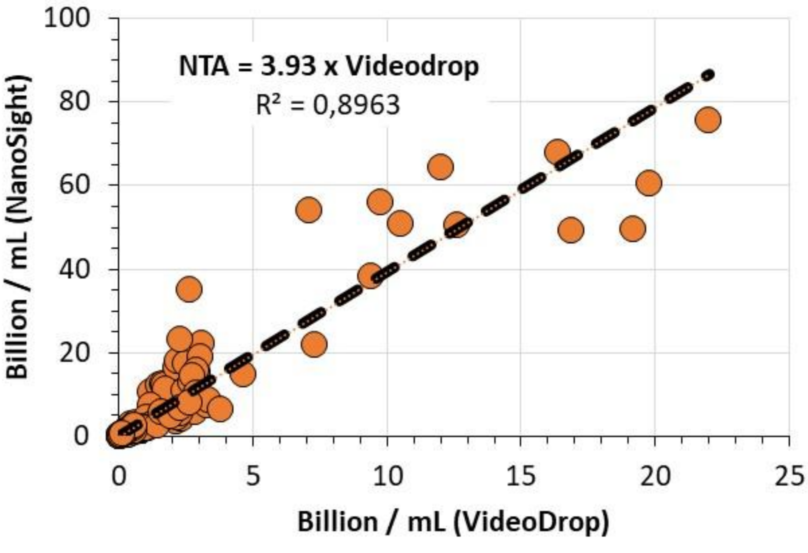

Figure S5: Correlation between Nanoparticle Tracking Analysis measurements performed with the NanoSight instrument and the VideoDrop instrument.

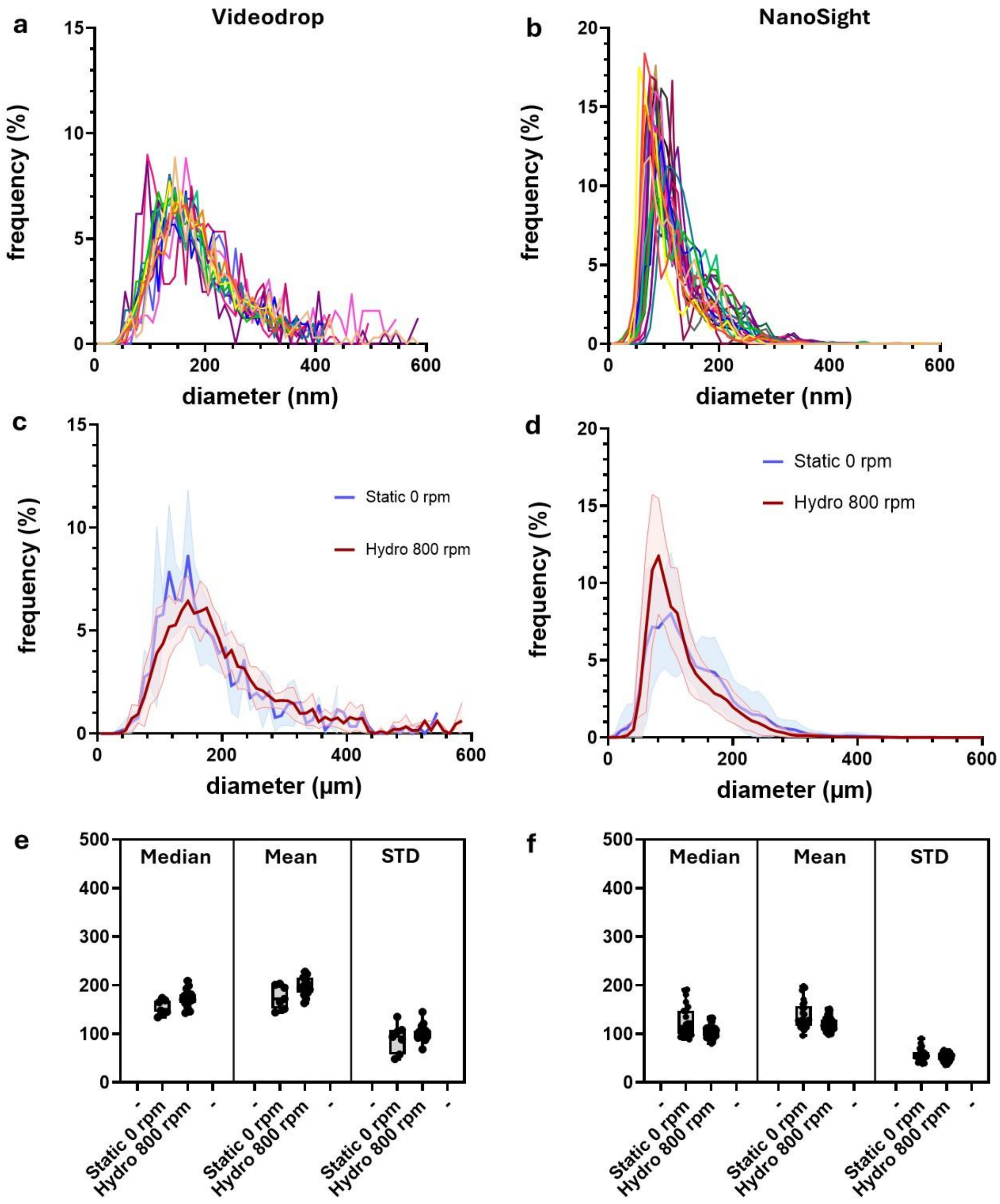

Figure S6. Size distribution data corresponding to the experiments shown in Figure 1, comparing the static control condition and the hydrodynamic condition (800 rpm), obtained from independent production batches and measured using both the Videodrop (a, c, e) and NanoSight (b, d, f) nanoparticle tracking analysis (NTA) instruments. **(a, b)** Individual size distributions from independent EV production batches under hydrodynamic stimulation (800 rpm), overlaid in the same graph for direct comparison. **(c, d)** Mean size distributions derived from the same datasets, with the standard deviation represented by a light connecting curve and a shaded area surrounding the mean distribution curve. **(e, f)** Box-and-whisker plots displaying all individual values from the independent measurements.

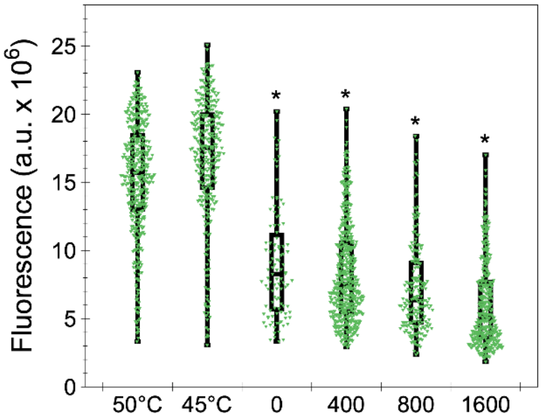

Figure S7: Caspase 3/7 activation fluorescence assay.

Spheroids were generated using the agarose microwell array described previously and subjected to rotation speeds of 0, 400, 800, or 1600 rpm for 3 h in rotating tubes to assess potential apoptotic effects of hydrodynamic stress on producer cells. Two positive controls were included in parallel: spheroids exposed to hyperthermia at 50 °C for 30 min followed by 3 h incubation at 37°C, or 45°C for 1 h followed by 6 h incubation at 37 °C. Apoptosis was analyzed using the CellEvent™ Caspase 3/7 Detection Reagent (C10723, Thermo Fisher Scientific). Spheroids were incubated with the fluorophore at 1 µM for 30 min, and fluorescence was recorded using a plate reader (Ensight Multimode Plate Reader, PerkinElmer) in fluorescence mode (excitation 465 nm, emission 535 nm). Fluorescence quantification was performed with Fiji (ImageJ, NIH) using Corrected Total Cell Fluorescence (CTCF), which accounts for the region of interest while subtracting background signal. Quantifications were performed on: $n > 400$ spheroids (50°C control), $n > 300$ (45°C control), $n > 100$ (0 rpm control), $n > 500$ (400 rpm), $n > 200$ (800 rpm), and $n > 400$ (1600 rpm). Statistical comparisons between groups were carried out using a two-sample Student's *t*-test (MATLAB, MathWorks Inc.). $p < 0.001$ vs. 45 °C control.

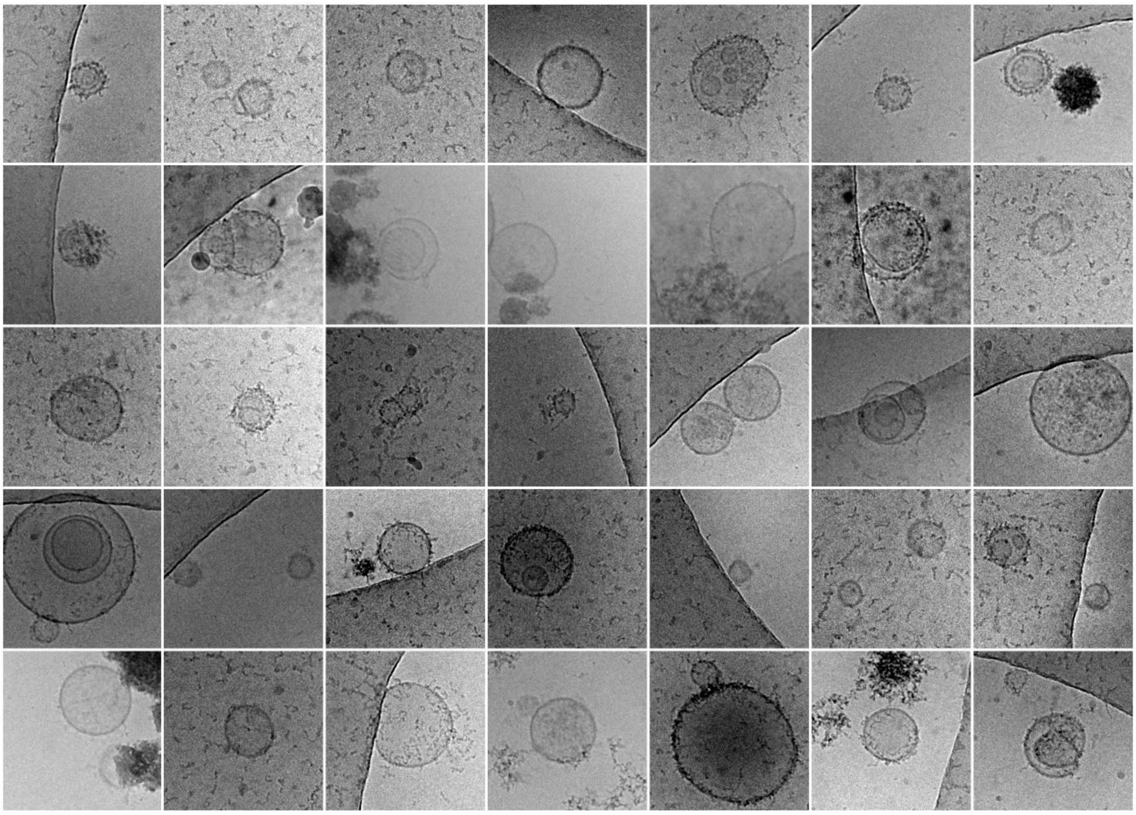

Figure S8: CryoTEM observation of EVs produced under the Starv2D condition. Each square represents an area of 300 nm x 300 nm.

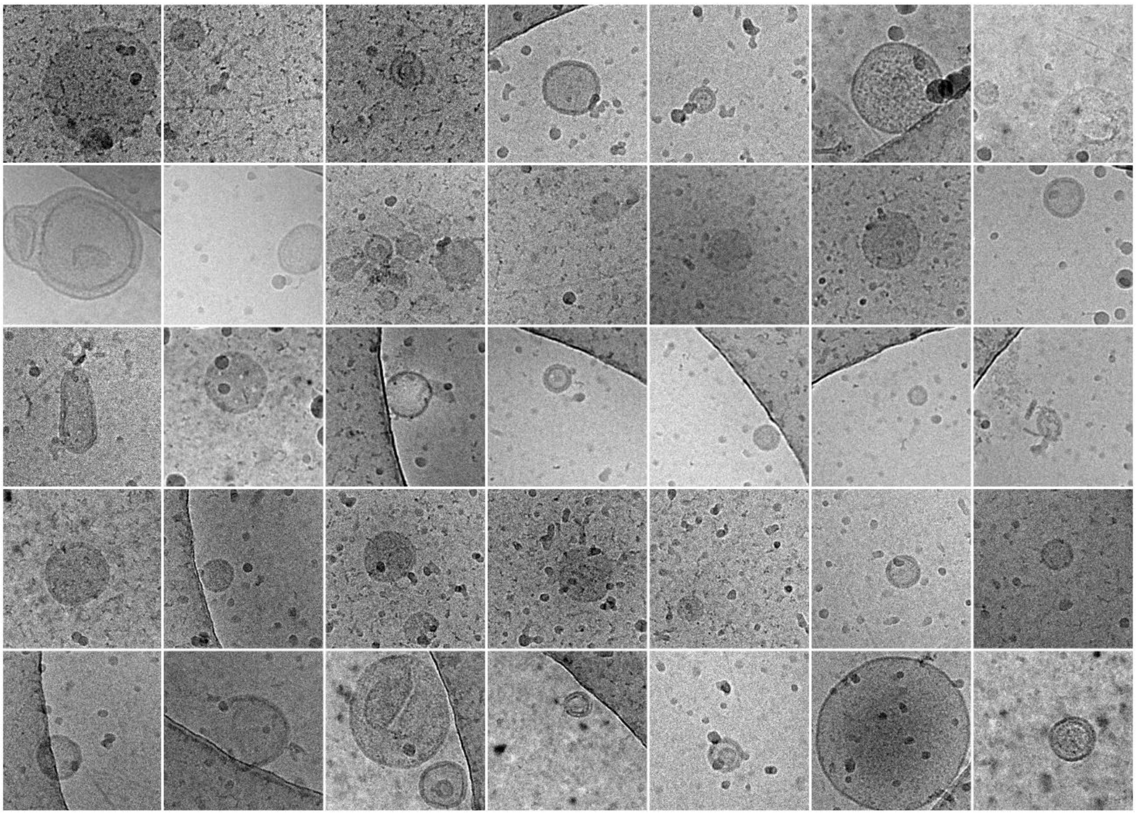

Figure S9: CryoTEM observation of EVs produced under the Starv3D condition. Each square represents an area of 300 nm x 300 nm.

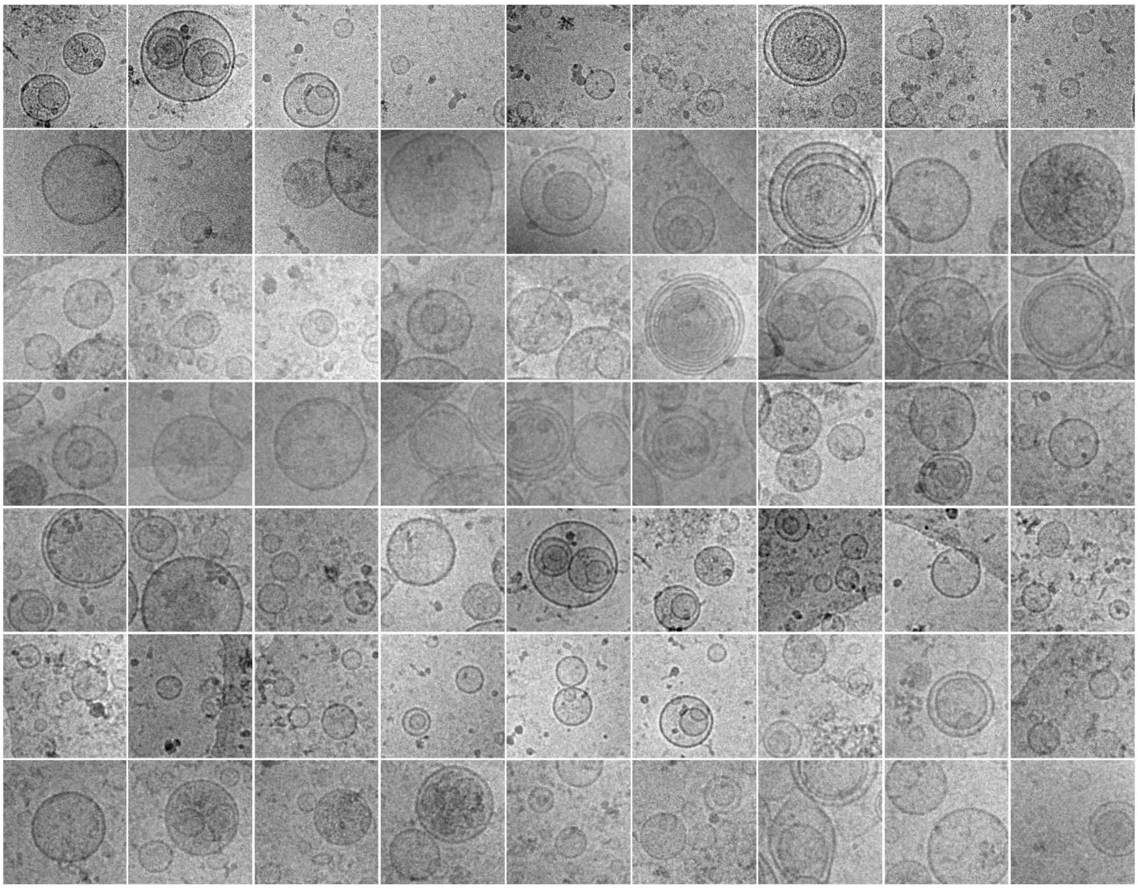

Figure S10: CryoTEM observation of EVs produced under the HydroCell condition. Each square represents an area of 300 nm x 300 nm.

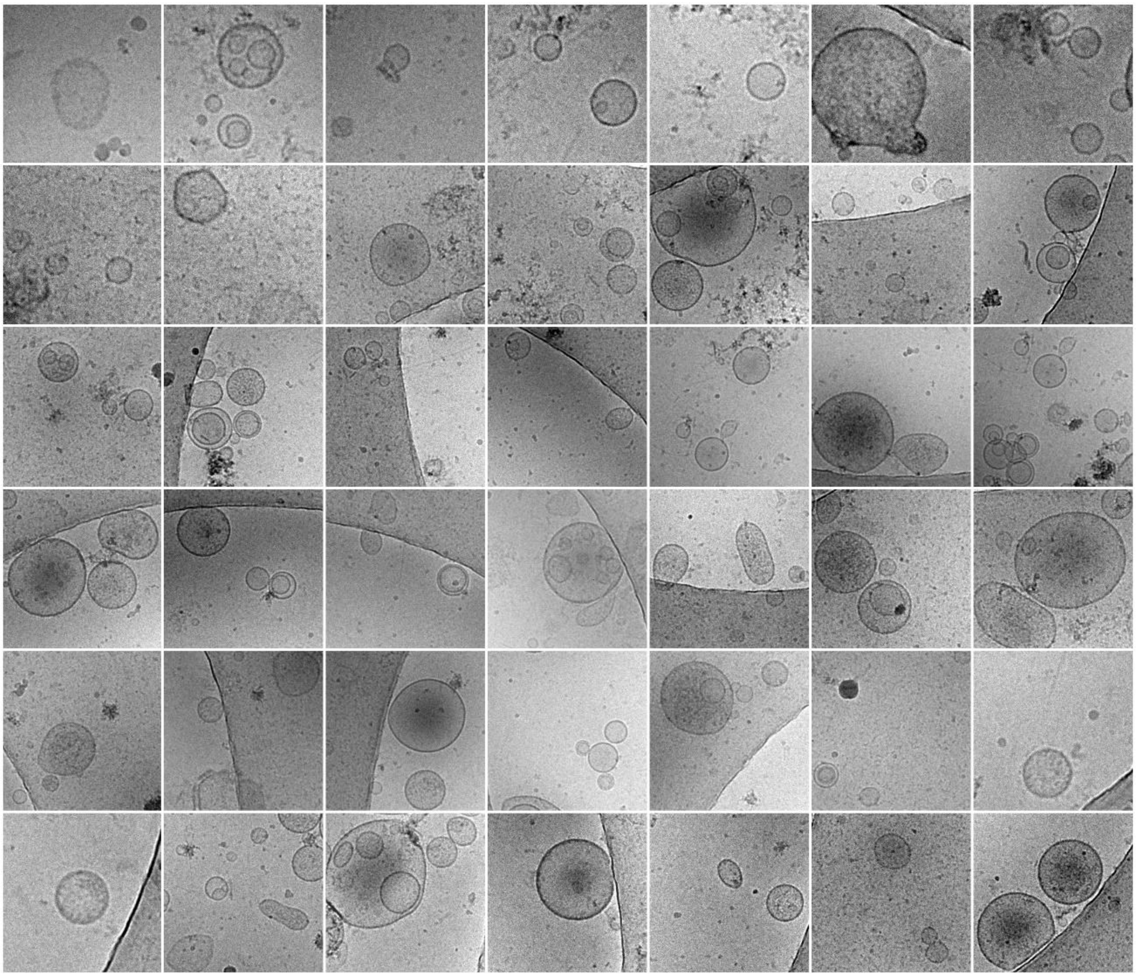

Figure S11: CryoTEM observation of EVs produced under the HydroSph condition. Each square represents an area of 300 nm x 300 nm.

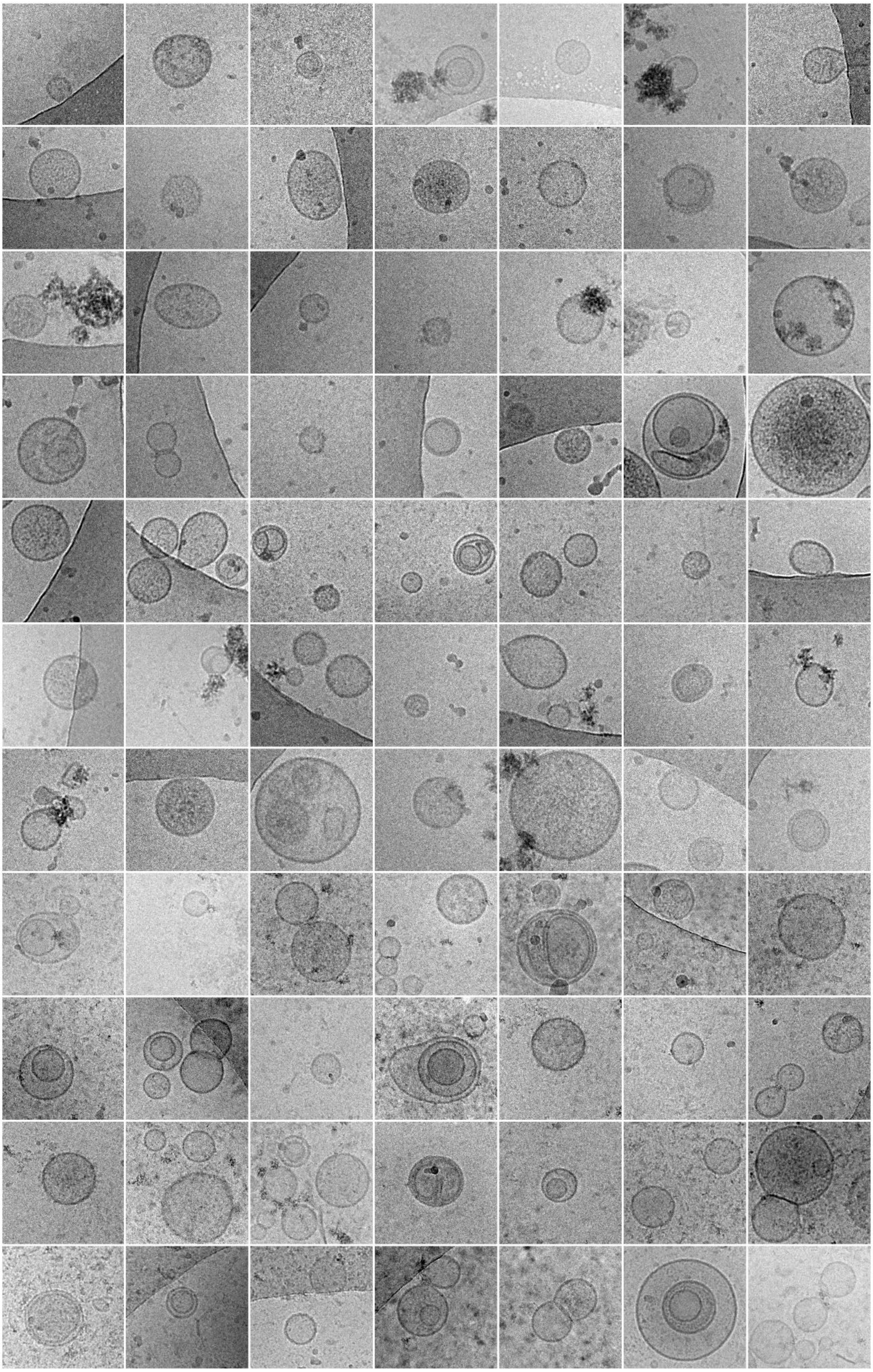

Figure S12: CryoTEM observation of EVs produced under the HydroSphIn condition. Each square represents an area of 300 nm x 300 nm.

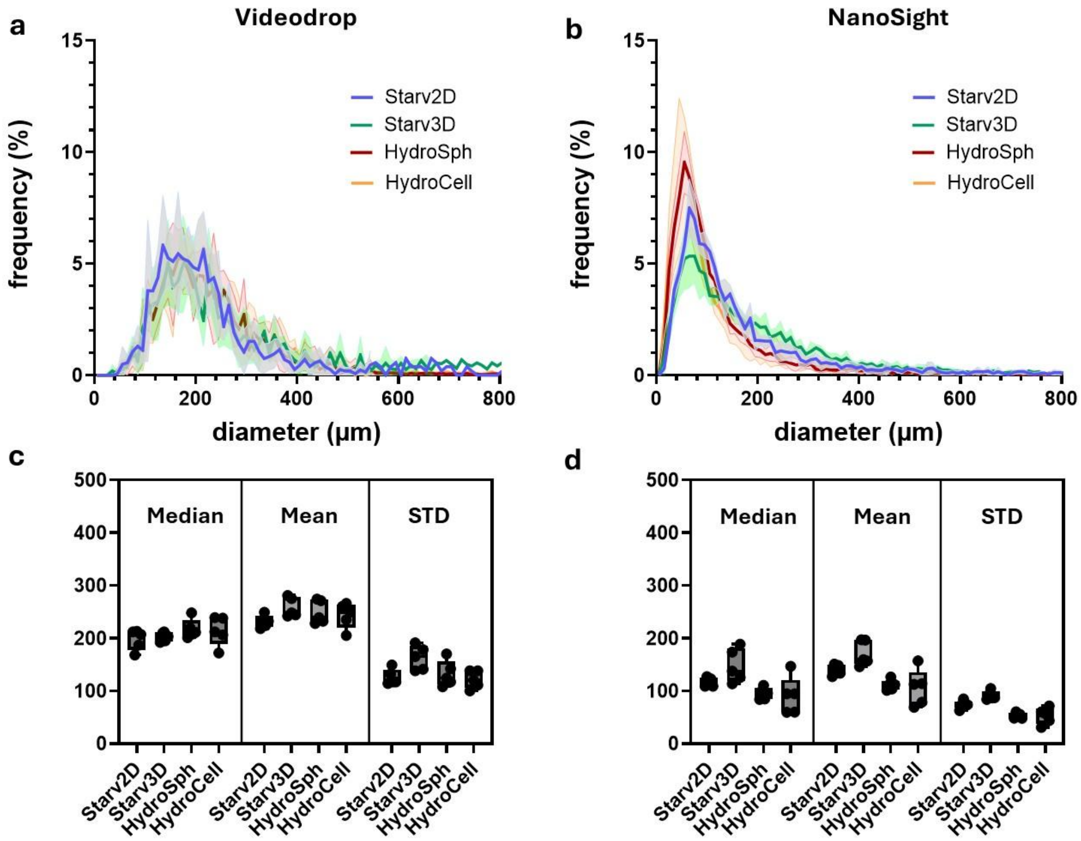

Figure S13 : Size distribution of EVs produced under starvation and hydrodynamic conditions, as measured by Videodrop (a, c) and NanoSight (b, d), across the independent production batches used for the whole proteome analysis shown in Figure 3. **(a, b)** Mean size distributions for each condition, with standard deviation represented as a shaded area and error bars plotted along the mean distribution curves. **(c, d)** Box-and-whisker plots showing all individual measurements from the independent EV production runs.

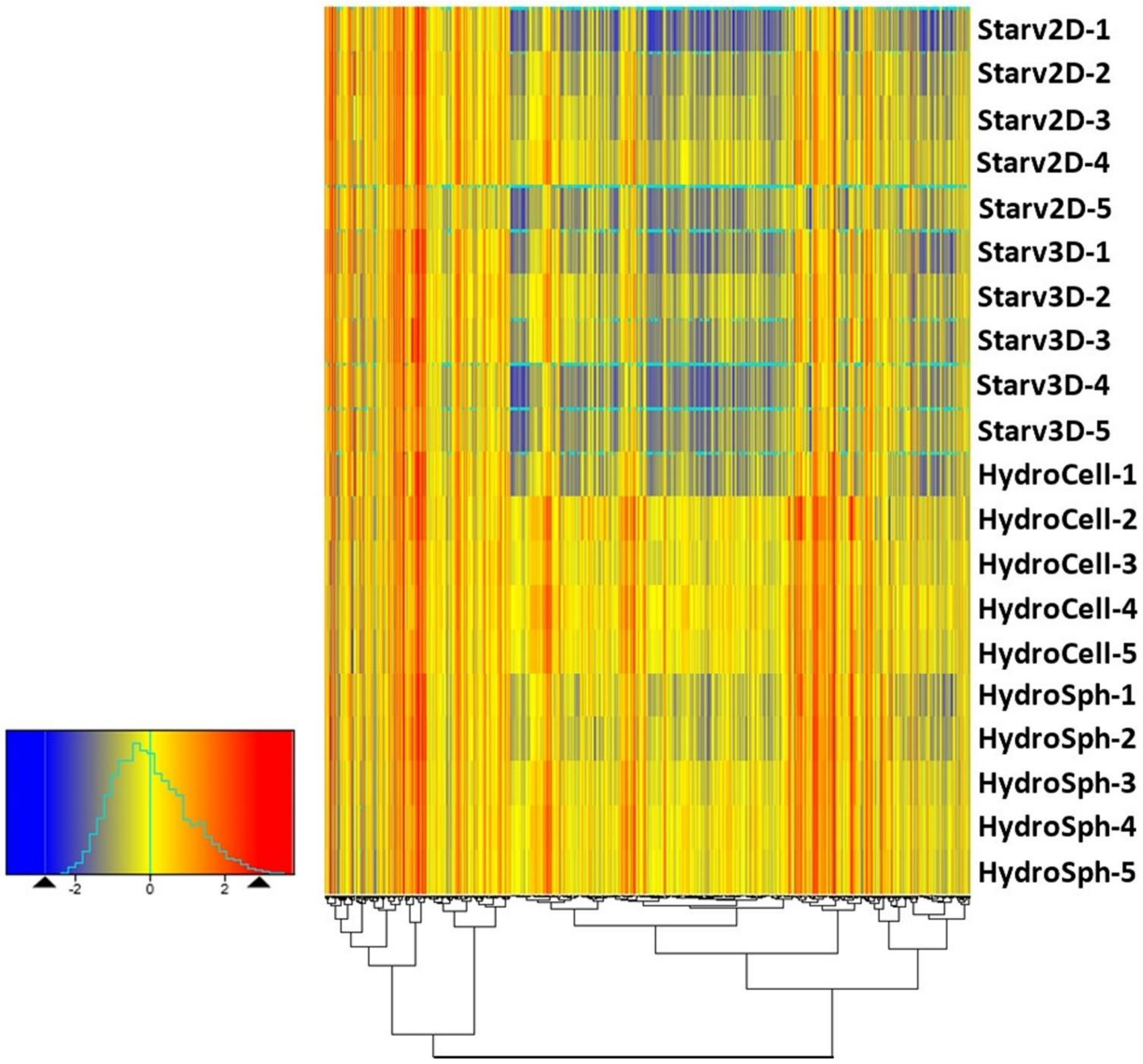

Figure S14: Clustering of all proteins for all conditions.

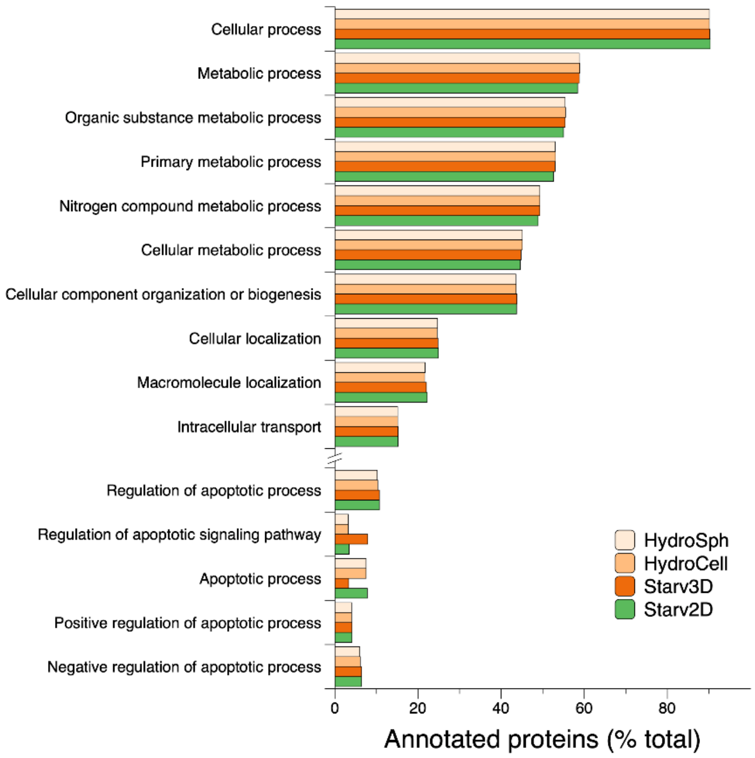

Figure S15: Gene ontology analysis showing the top 10 significantly enriched Biological Process terms (based on p-value) by gene count percentage, as well as the top 5 apoptosis-related annotations. The analysis was performed using the complete list of proteins identified in each group, restricted to proteins detected with at least three distinct peptides, with the whole human genome used as background.

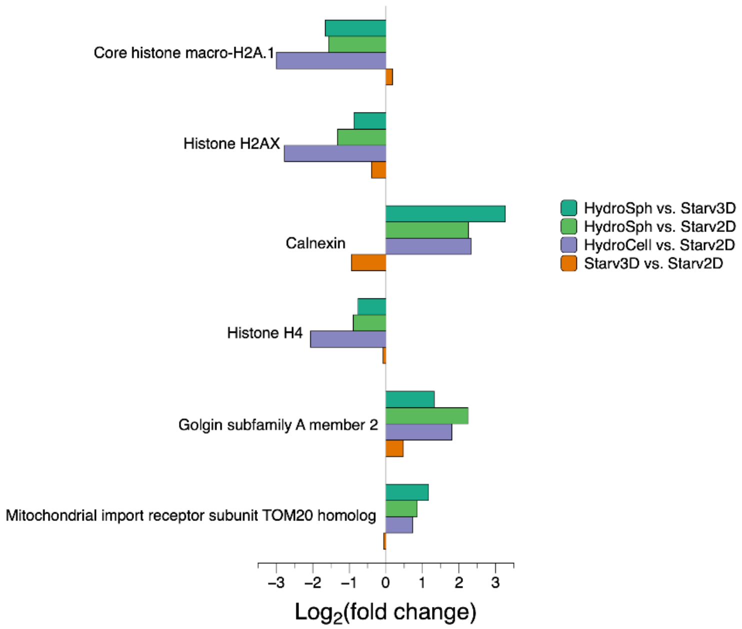

Figure S16: Enrichment analysis of non-EV marker proteins. A list of 13 proteins was generated and mapped across all paired conditions, with 6 proteins identified in the EV datasets: core histone macro-H2A.1 (H2AFY), histone H2AX (H2AX), calnexin (CANX), histone H4 (HIS1H4A), golgin subfamily A member 2 (GOLGA2), and mitochondrial import receptor subunit TOM20 homolog (TOMM20). Among these, CANX and GOLGA2 were found to be moderately enriched under hydrodynamic conditions compared with both Starv3D and Starv2D conditions.

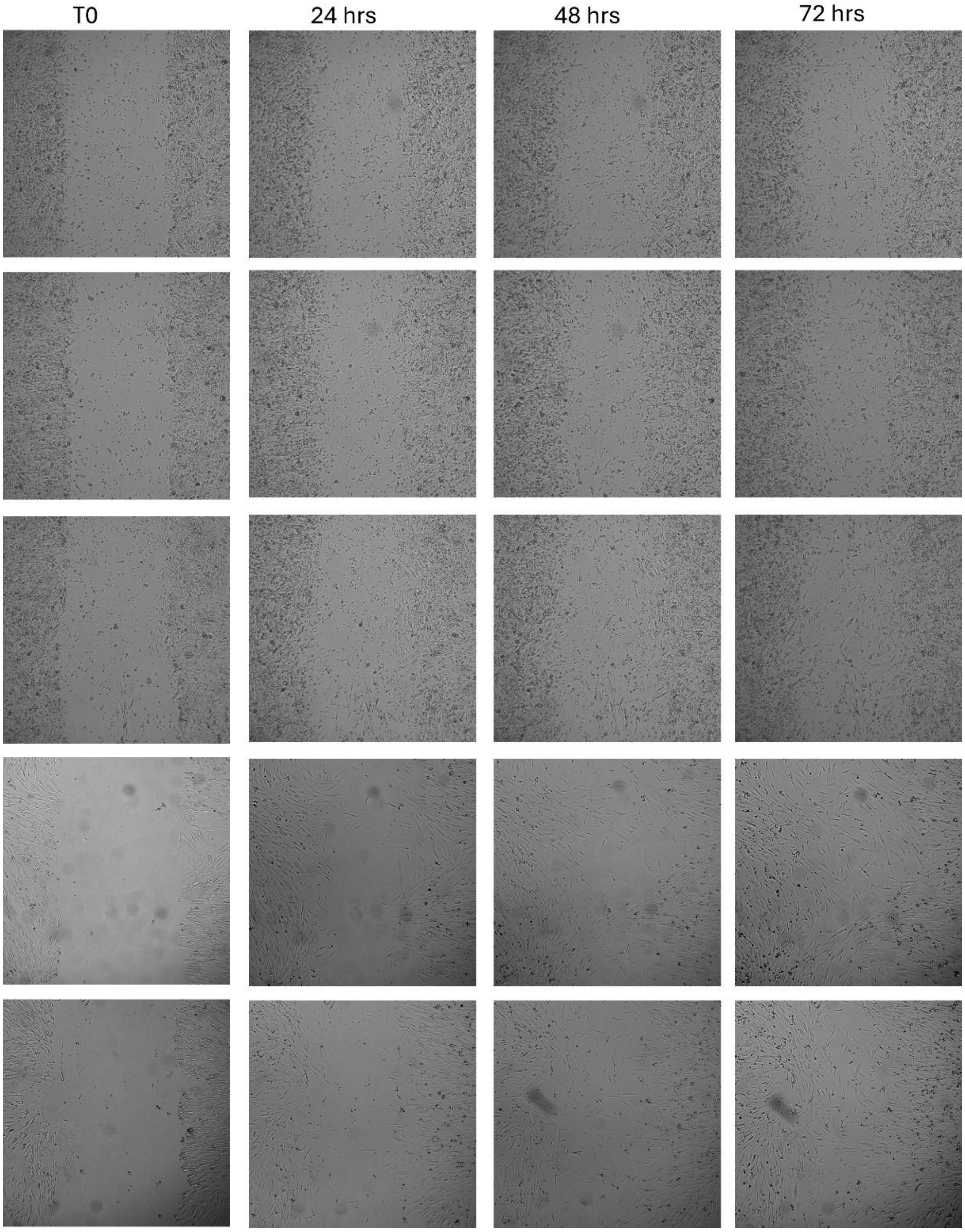

Figure S17a: Images depicting the initial wound gap (T0) and wound healing at the 24-hour, 48-hour and 72-hour time points, for the FBS 0% condition.

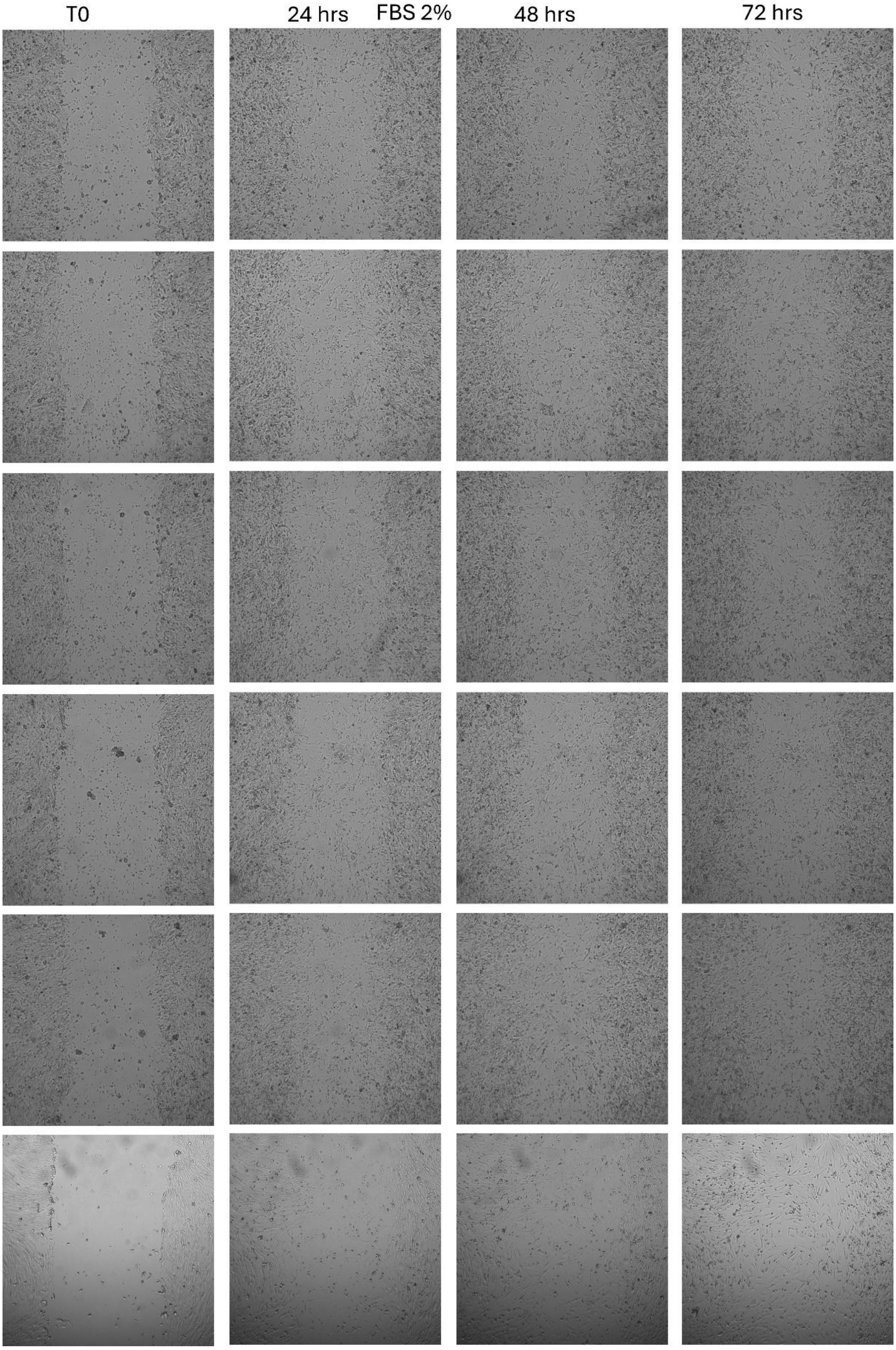

Figure S17b: Images depicting the initial wound gap (T0) and wound healing at the 24-hour, 48-hour and 72-hour time points, for the FBS 2% condition.

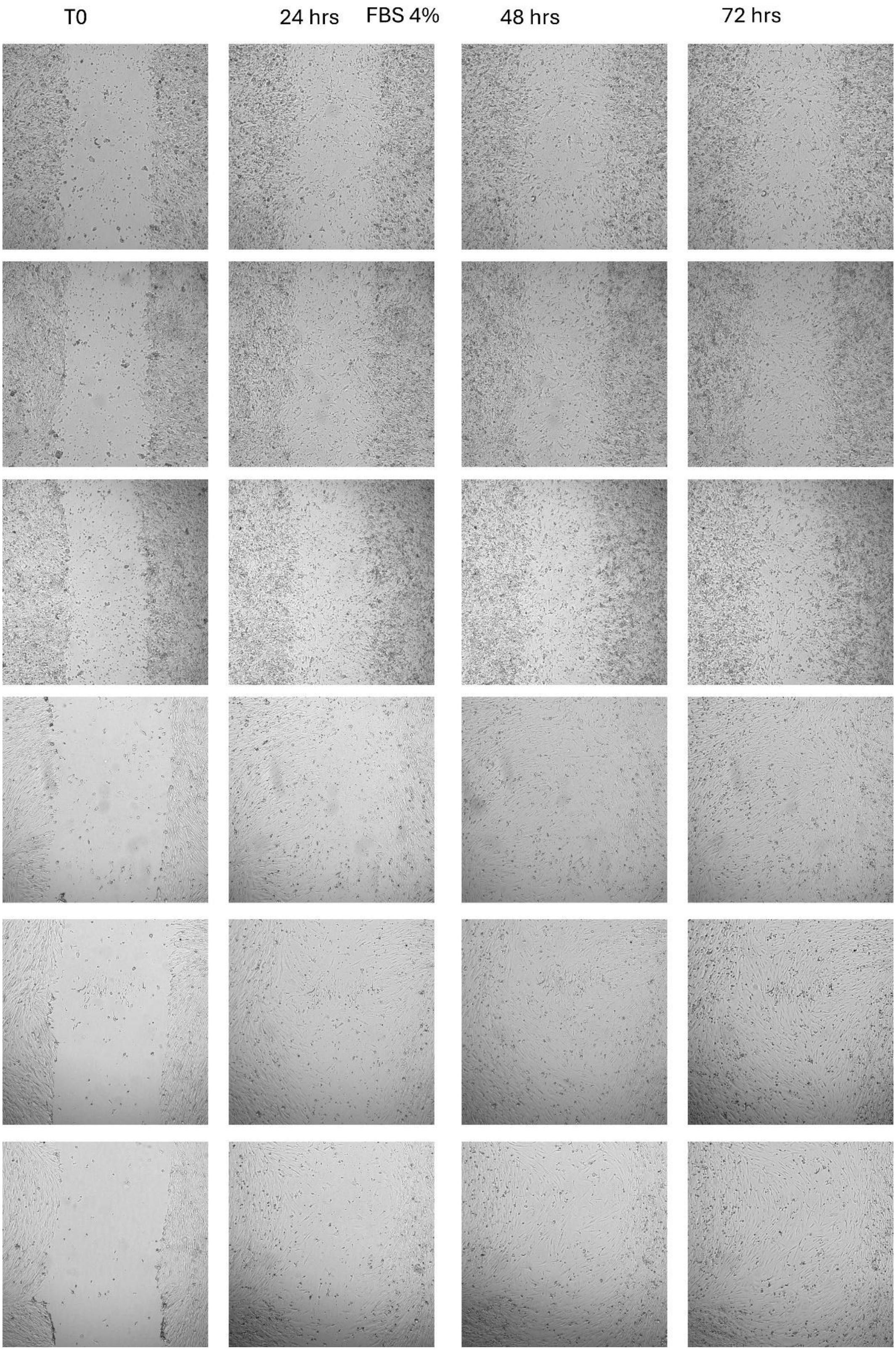

Figure S17c: Images depicting the initial wound gap (T0) and wound healing at the 24-hour, 48-hour and 72-hour time points, for the FBS 4% condition.

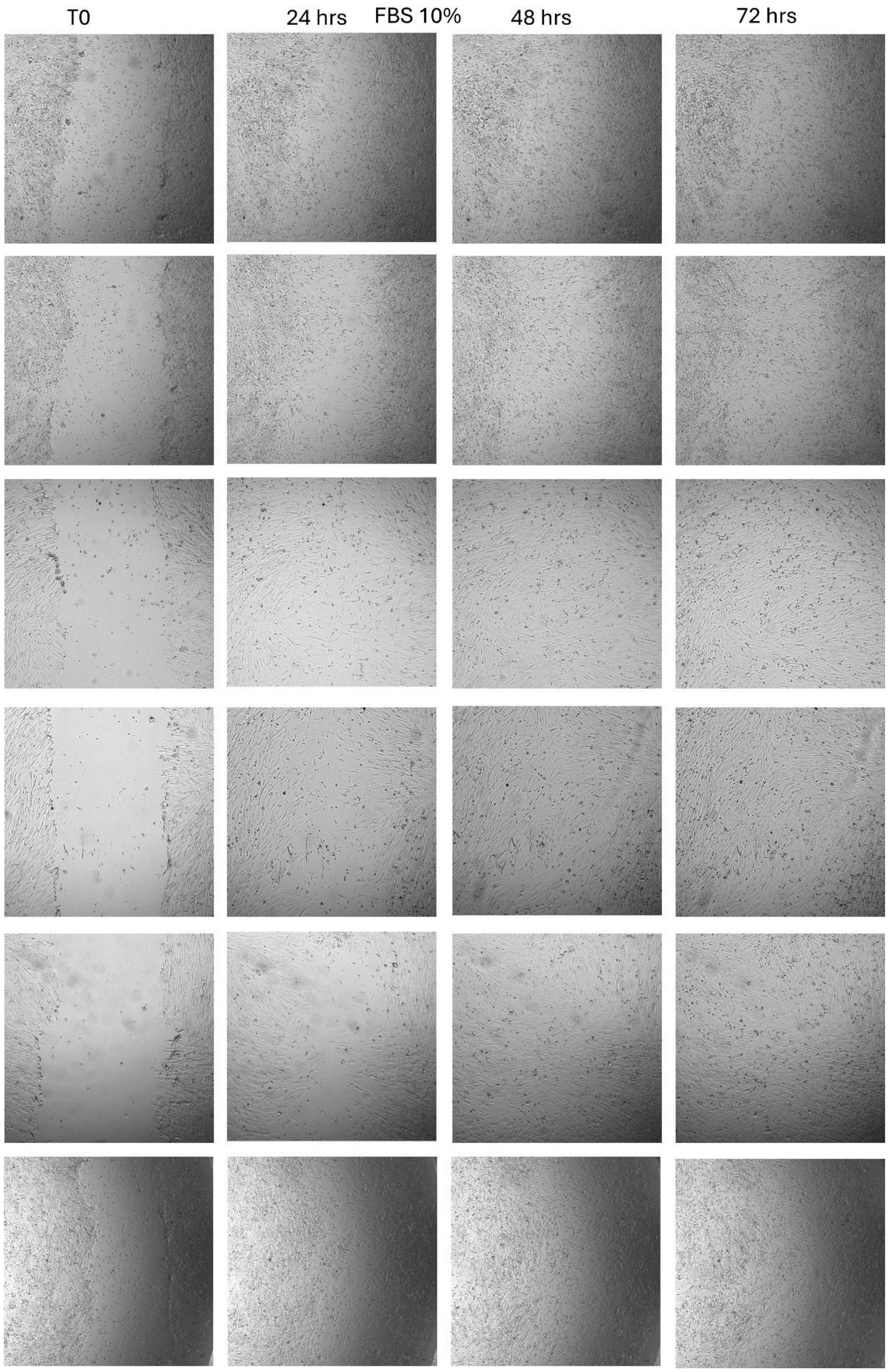

Figure S17d: Images depicting the initial wound gap (T0) and wound healing at the 24-hour, 48-hour and 72-hour time points, for the FBS 10% condition.

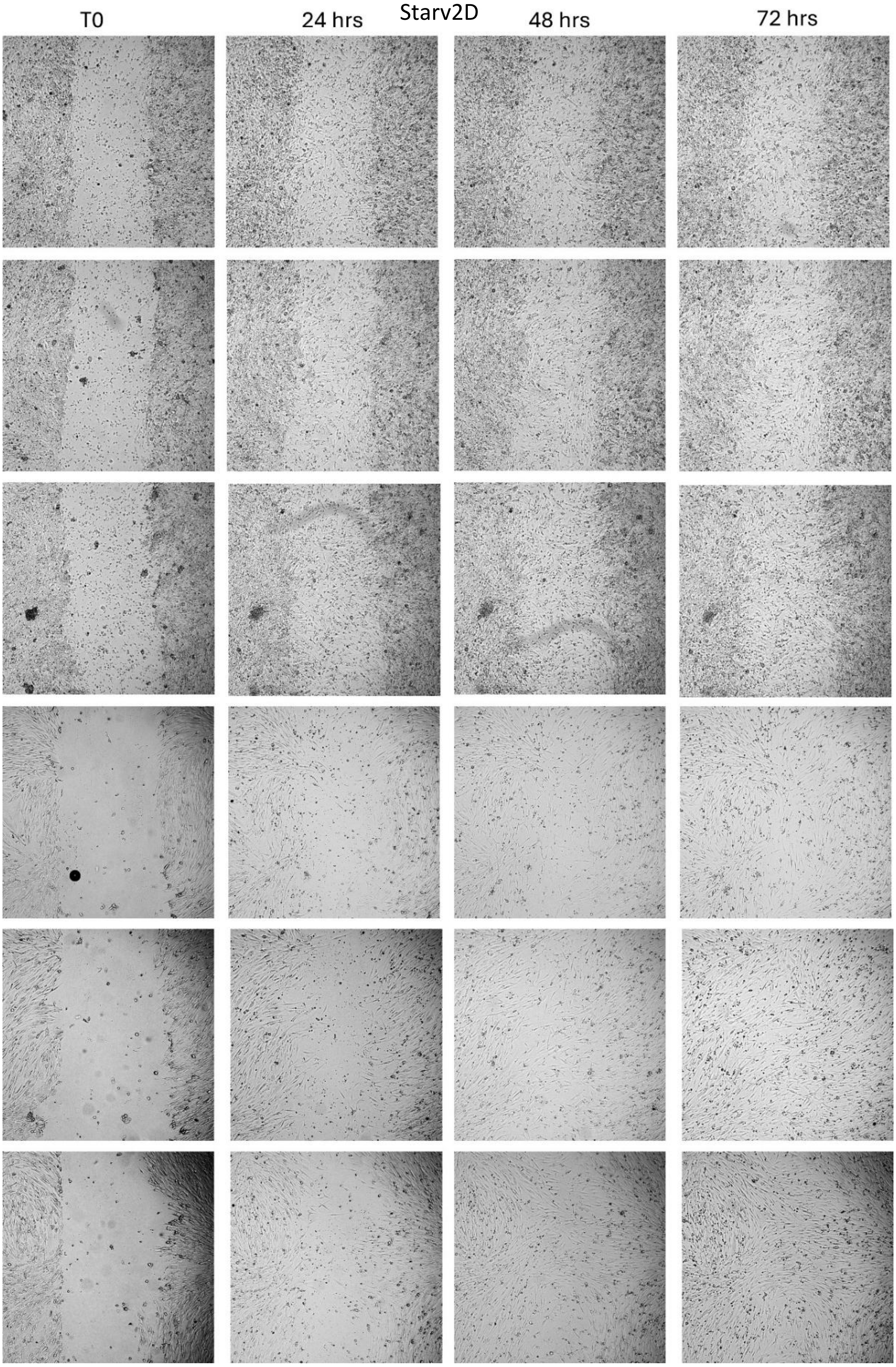

Figure S17e: Images depicting the initial wound gap (T0) and wound healing at the 24-hour, 48-hour and 72-hour time points, for the starvation 2D (Starv2D) condition.

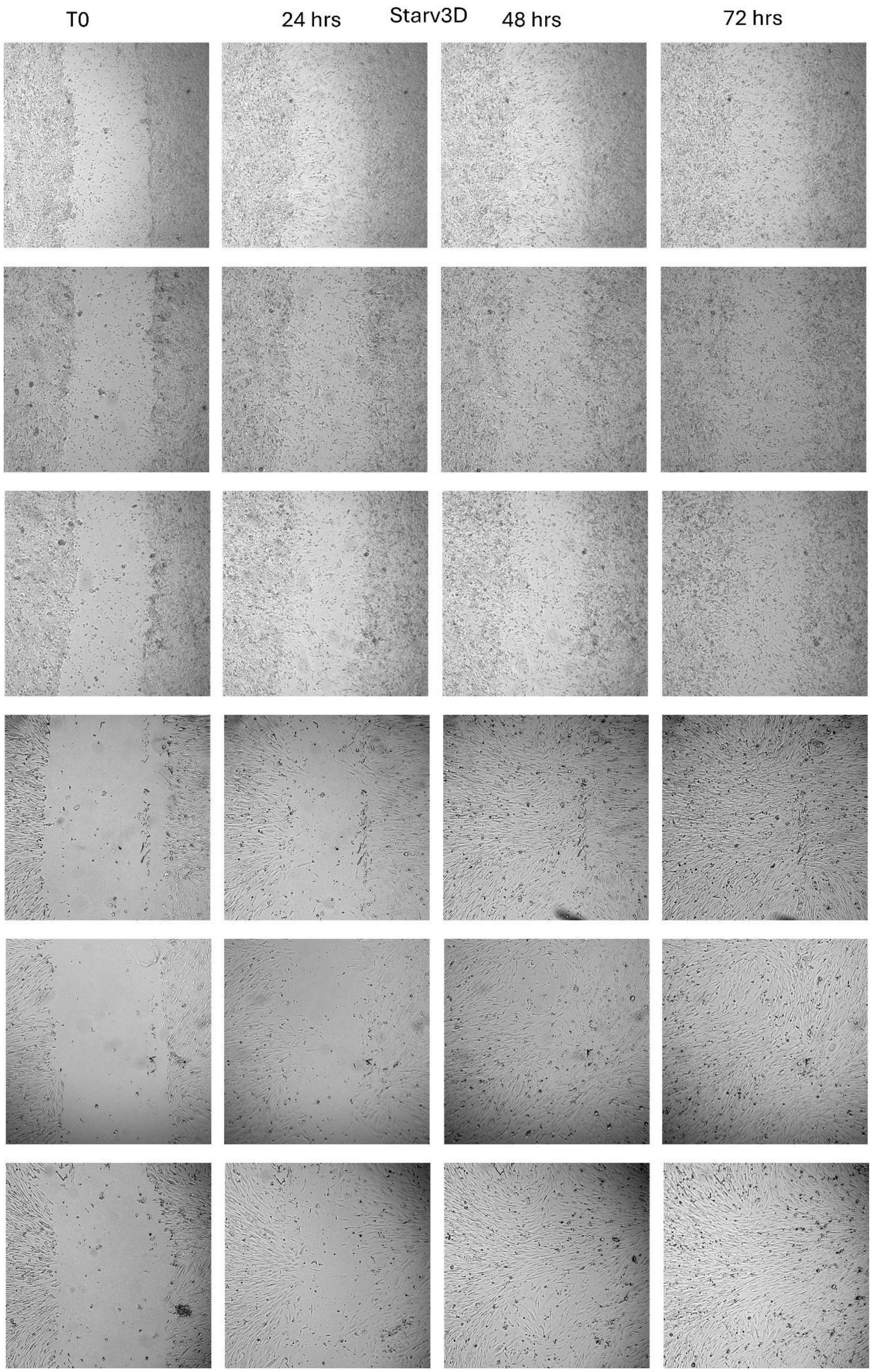

Figure S17f: Images depicting the initial wound gap (T0) and wound healing at the 24-hour, 48-hour and 72-hour time points, for the starvation 3D (Starv3D) condition.

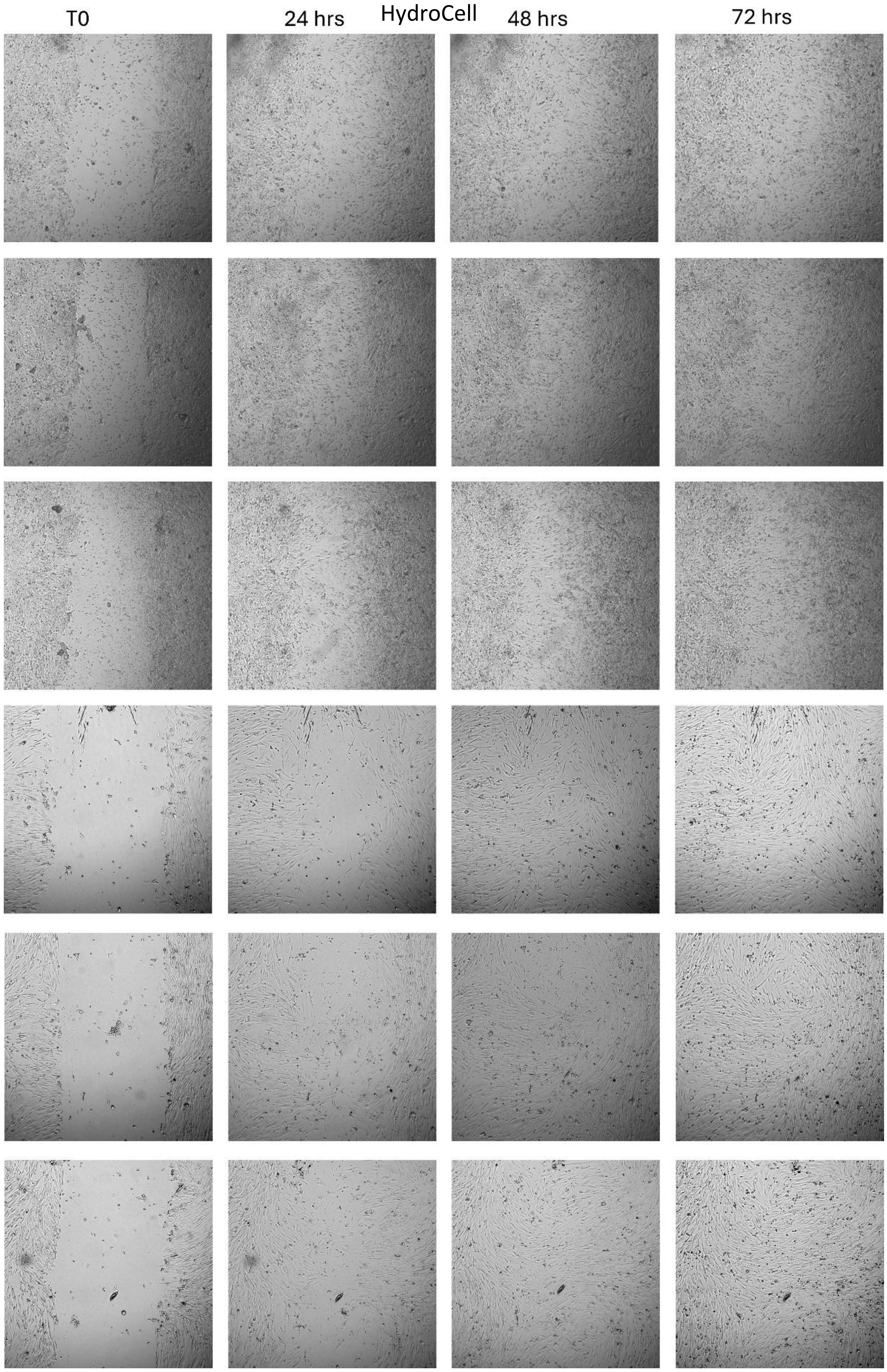

Figure S17g: Images depicting the initial wound gap (T0) and wound healing at the 24-hour, 48-hour and 72-hour time points, for the HydroCell condition.

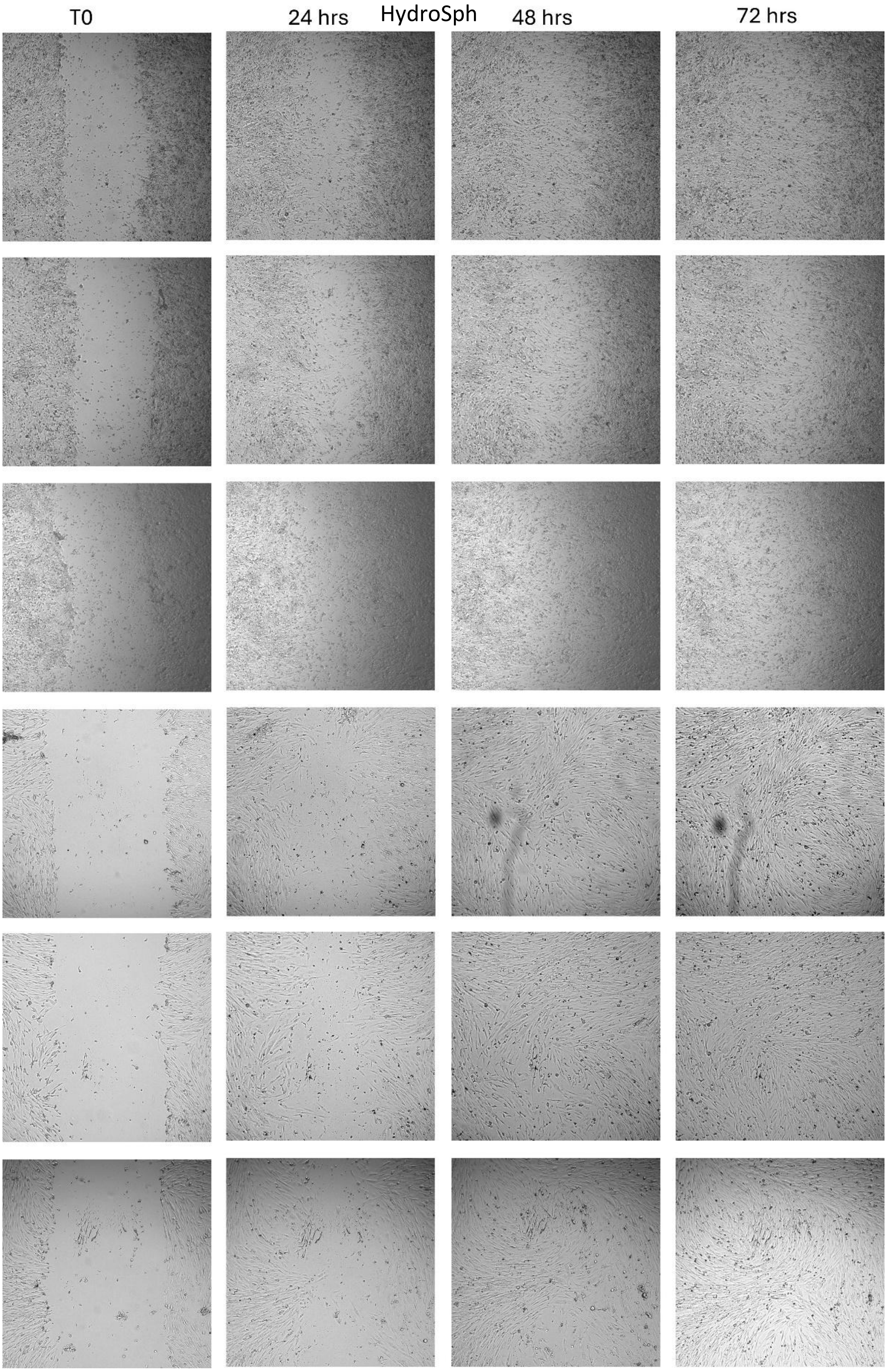

Figure S17h: Images depicting the initial wound gap (T0) and wound healing at the 24-hour, 48-hour and 72-hour time points, for the HydroSph condition.

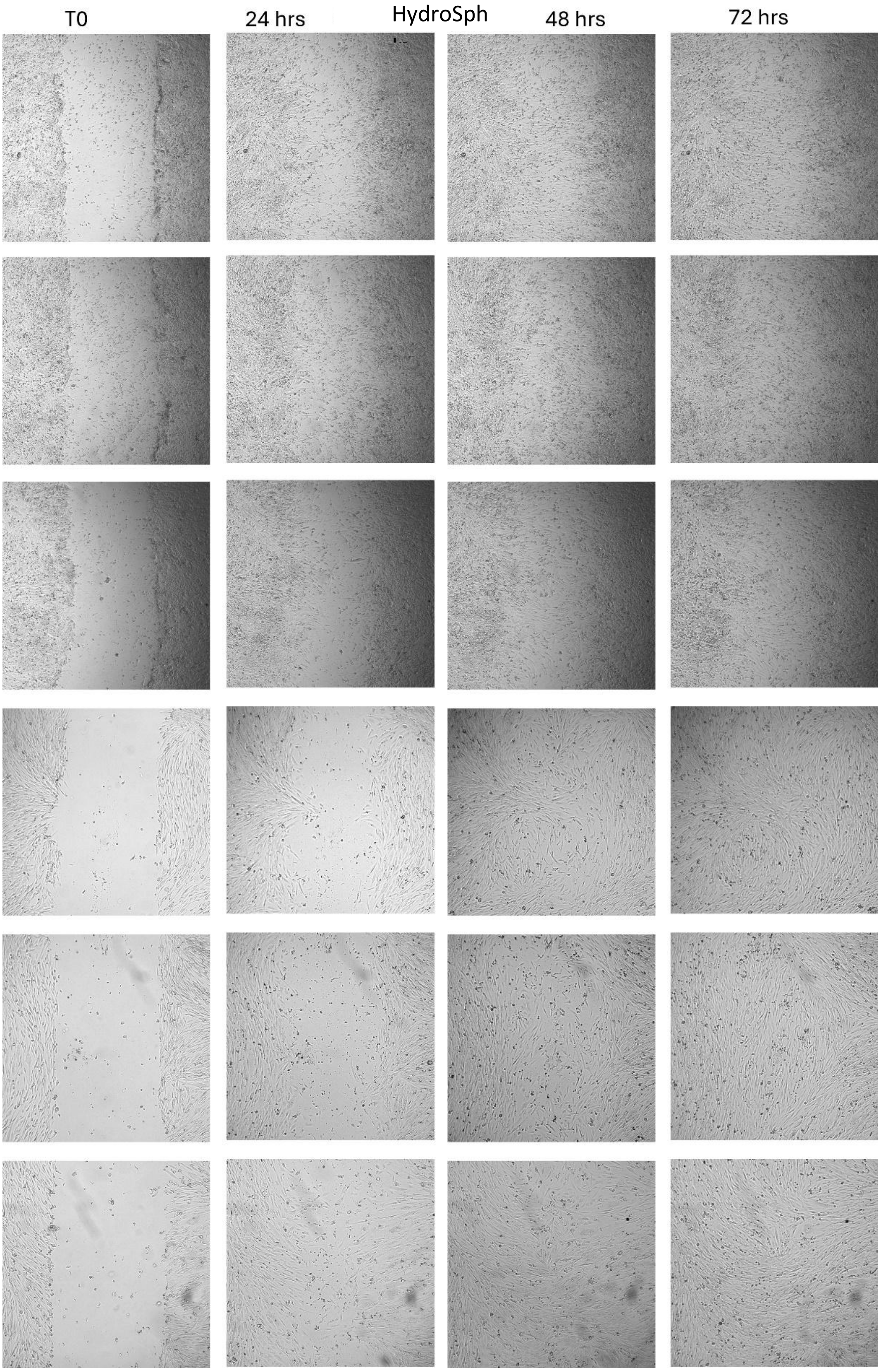

Figure S17i: Images depicting the initial wound gap (T0) and wound healing at the 24-hour, 48-hour and 72-hour time points, for the HydroSphIn condition.

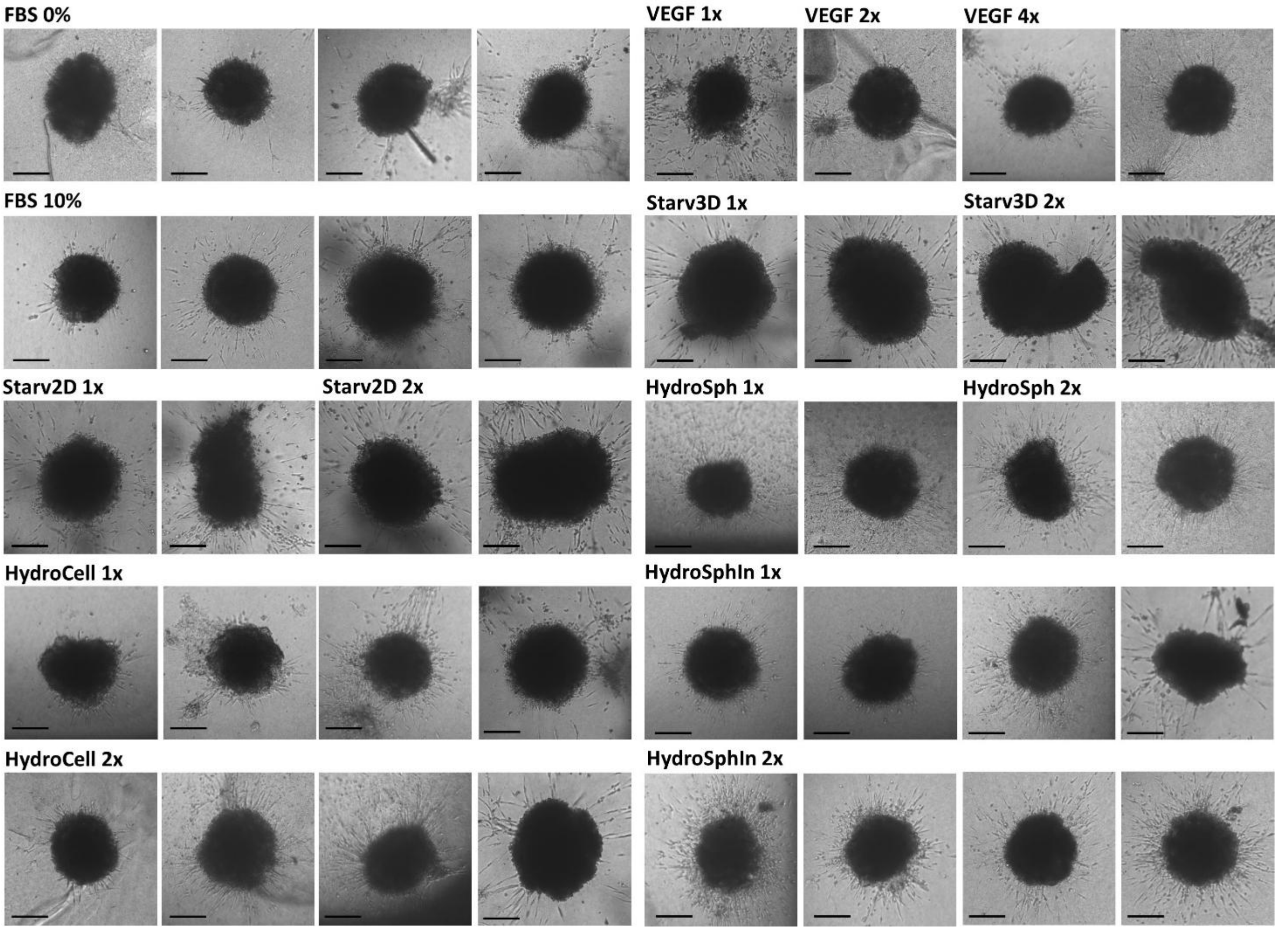

Figure S18: Images of sprout formation from endothelial spheroids, illustrating the neo-angiogenesis capacity, for all conditions depicted in Fig.3c and Fig.5d. Scale bars = 100 µm.

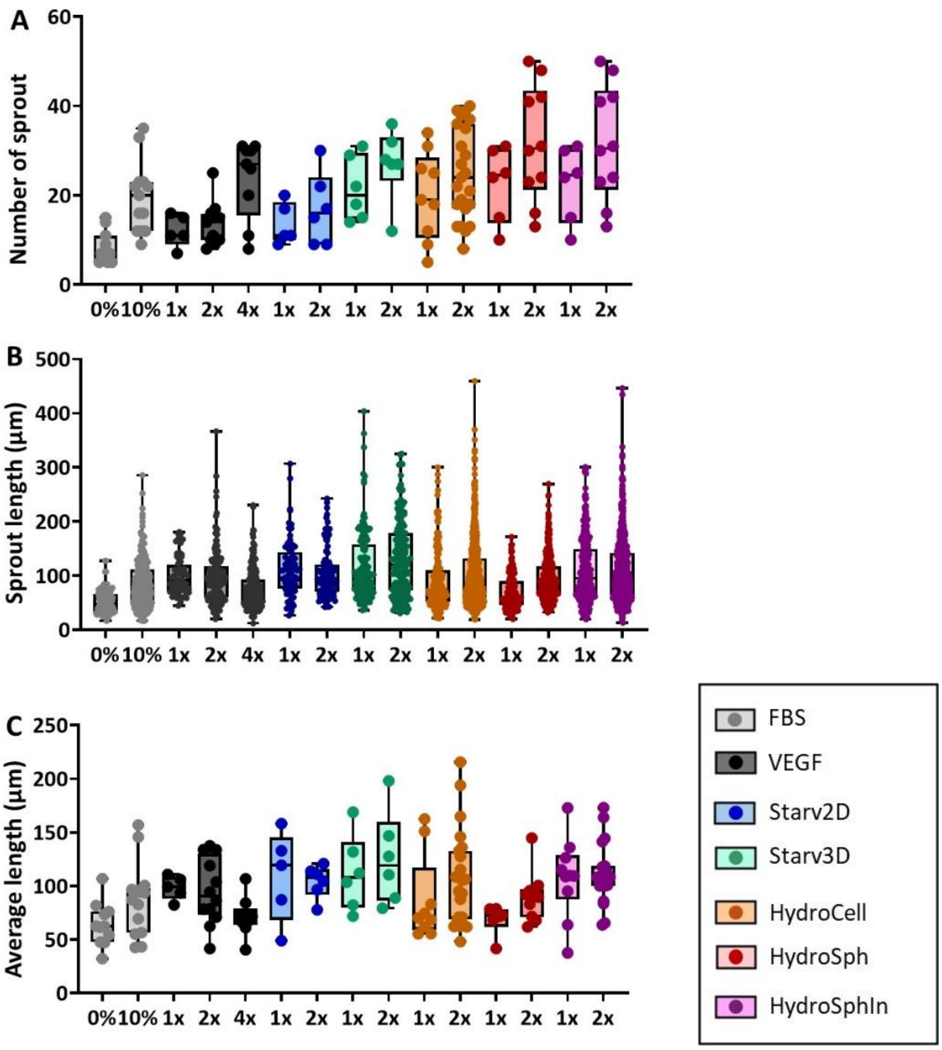

Figure S19: Sprout formation quantification in endothelial spheroids. **A**: number of sprouts. **B**: Individual measurement of the length of each sprout for all events. **C**: Average sprout length calculated for each individual spheroid. Conditions tested include FBS at 0% and 10%, VEGF at three doses 12.5 (1x), 25 (2x), 50 (4x) ng/mL, and EVs from the conditions Starv2D, Starv3D, HydroCell, and HydroSph (1x = $5x10^7$ EVs, equivalent to a concentration of 10^9 EVs per mL).

	LPS only	Dexa 8x	Dexa 4x	Dexa 2x	Dexa 1x
**Starv2D 4x**	0,000567182	0,001642196	0,24849847	0,772564721	0,001207196
**Starv2D 2x**	0,000831744	1,782E-06	0,00585099	0,336468818	0,000417837
**Starv2D 1x**	4,55943E-05	8,8716E-05	0,00209507	0,011348834	0,9799232
**Starv3D 4x**	4,55943E-05	1,27319E-05	0,34829428	0,072738532	4,00062E-06
**Starv3D 2x**	0,000283189	0,000159555	0,07616135	0,954259303	0,00027476
**Starv3D 1x**	1,94424E-06	1,75985E-05	0,00137734	0,017386703	0,114487366
**HydroCell 4x**	1,94424E-06	0,338016459	7,1995E-09	2,17147E-05	1,64481E-06
**HydroCell 2x**	1,27054E-05	0,000226663	0,07272318	0,001522335	1,07364E-06
**HydroCell 1x**	1,76787E-06	7,02416E-06	0,00882438	0,403111139	0,00038815
**HydroSph 4x**	1,27529E-06	0,290508375	1,8085E-09	5,81527E-06	3,3169E-07
**HydroSph 2x**	5,01853E-05	0,093517984	0,2169084	0,02947974	0,000328354
**HydroSph 1x**	2,27085E-06	0,002383348	0,16791715	0,989631998	0,003276217

Table S1: P-values for the comparisons between LPS, dexamethasone at all doses, and EVs produced in the different conditions, at all doses.

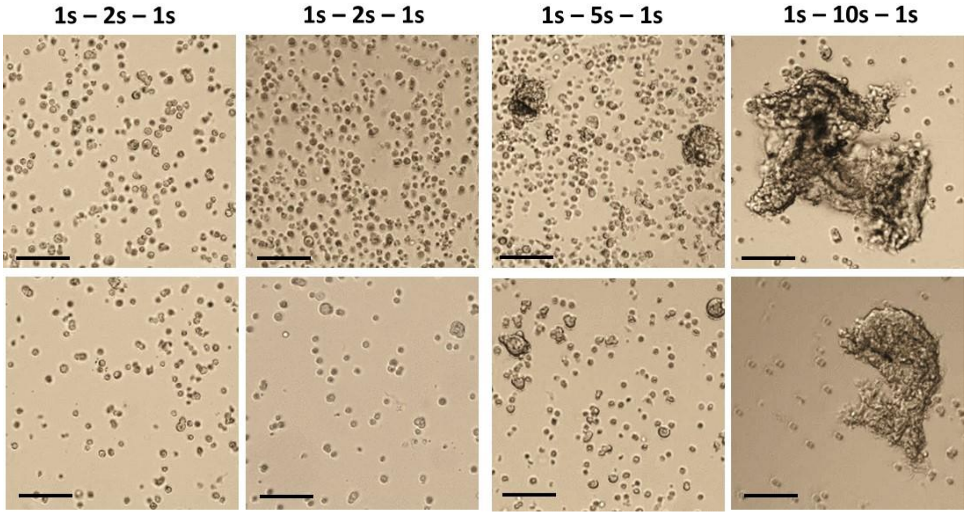

Figure S20a. Additional images of spheroids produced under each rotation condition, with the rotation speed set at 60 rpm and the acceleration at 120 rpm. The first element noted above each panel describes the period duration in one direction; the second element denotes the pause duration, and the third element the period duration in the opposite direction. Scale bars = 200 µm.

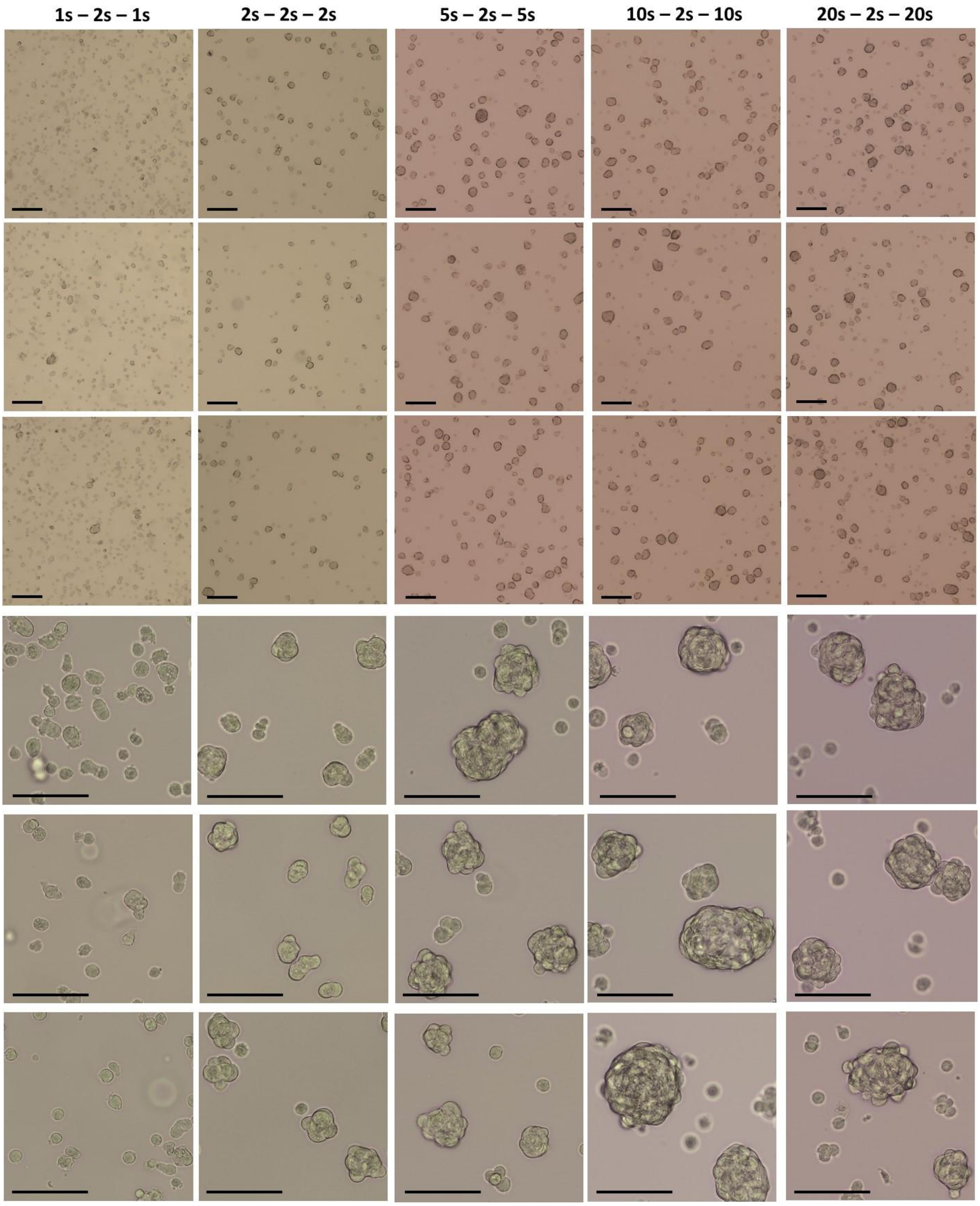

Figure S20b. Additional images of spheroids produced under each rotation condition, with the rotation speed set at 60 rpm and the acceleration at 120 rpm. The first element noted above each panel describes the period duration in one direction; the second element denotes the pause duration, and the third element the period duration in the opposite direction. Scale bars = 200 µm.

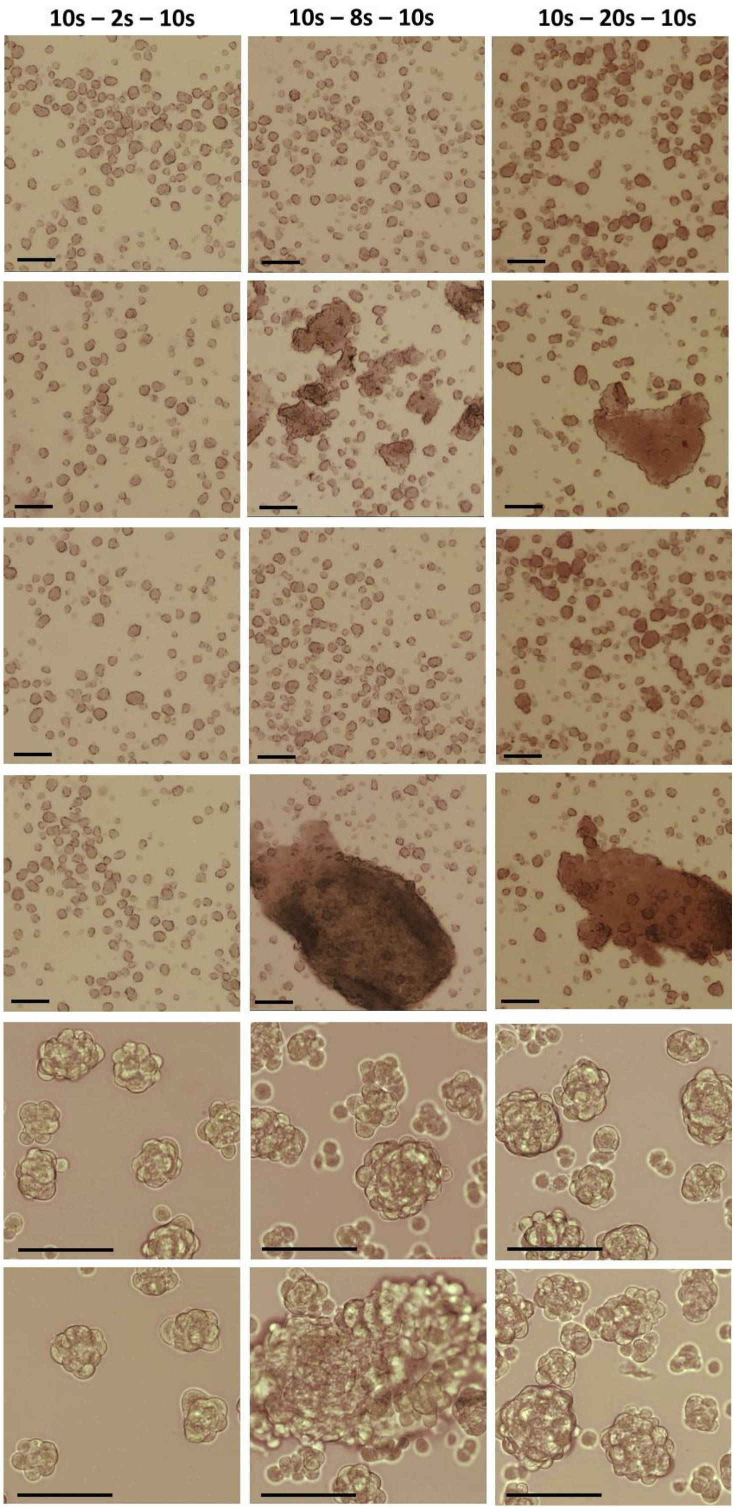

Figure S20c. Additional images of spheroids produced under each rotation condition, with the rotation speed set at 60 rpm and the acceleration at 120 rpm. The first element noted above each panel describes the period duration in one direction; the second element denotes the pause duration, and the third element the period duration in the opposite direction. Scale bars = 200 µm.

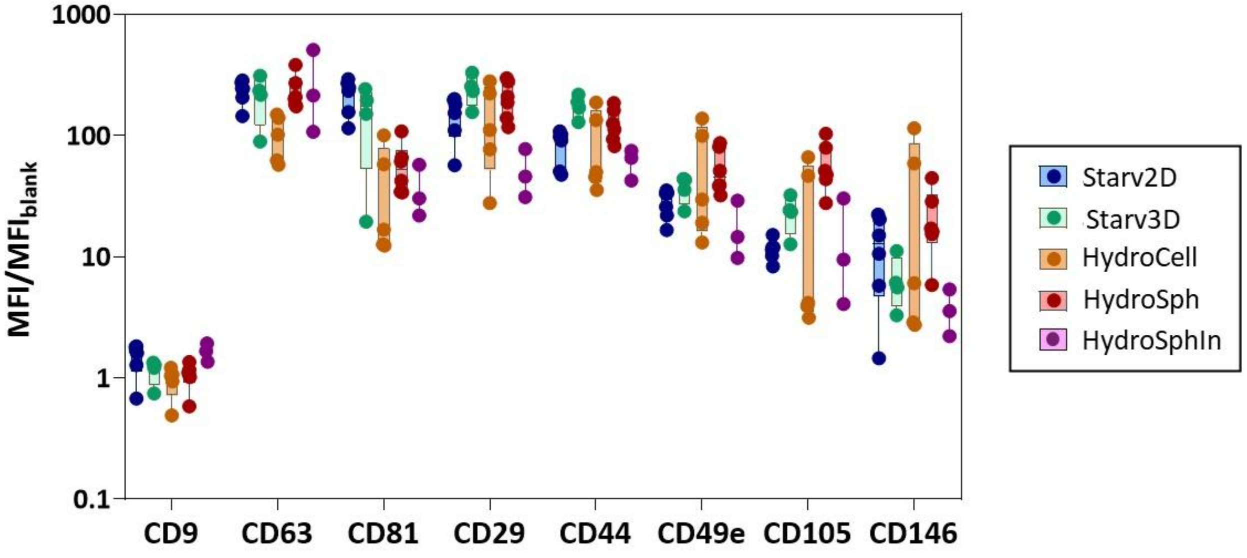

Figure S21: **Bead-Based Multiplex Flow Cytometry Assay (MACSPlex)**

Bead-based fluorescence flow cytometric analysis of EV-specific and mesenchymal-specific protein markers in extracellular vesicles produced in hydrodynamic conditions from spheroids formed in agarose microwells (HydroSph) or formed in situ within the tubes (HydroSphIn) or from individual cells (HydroCell), and for EVs produced upon serum starvation from spheroids (Starv3D) or adherent cells (Starv2D). Results are presented as ratios of mean fluorescence intensity with respect to blank. Each point corresponds to an independent experiment.

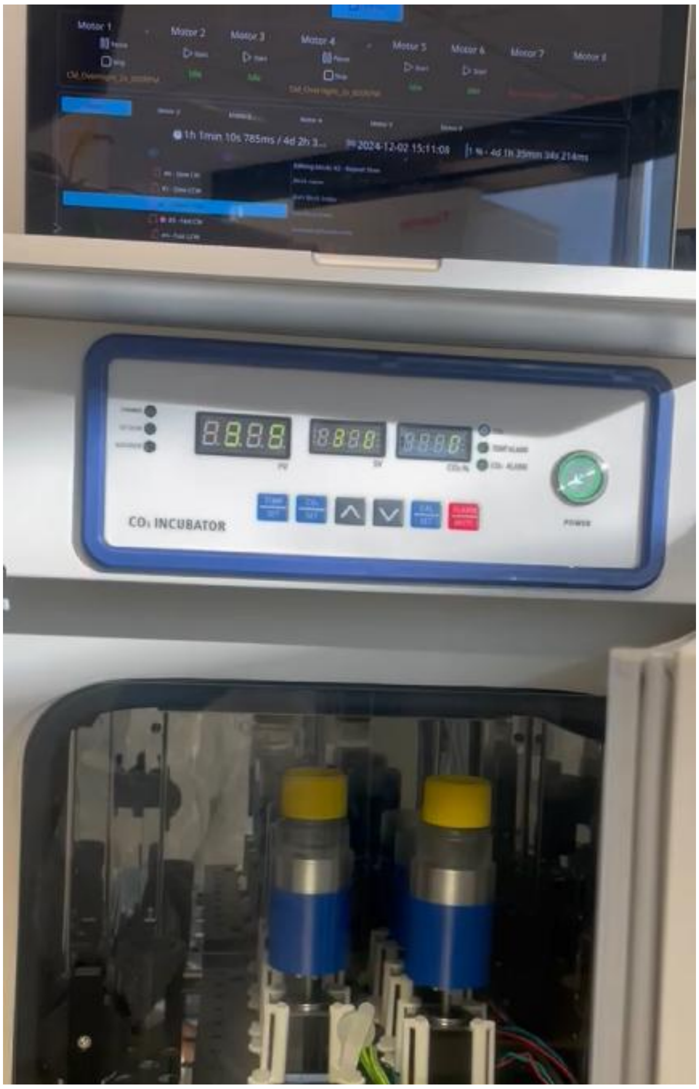

Figure S22: Built prototype of the bi-directional rotating tube technology. Photograph shows 6 rotating tubes each mounted on a motor and placed inside a humidified incubator.

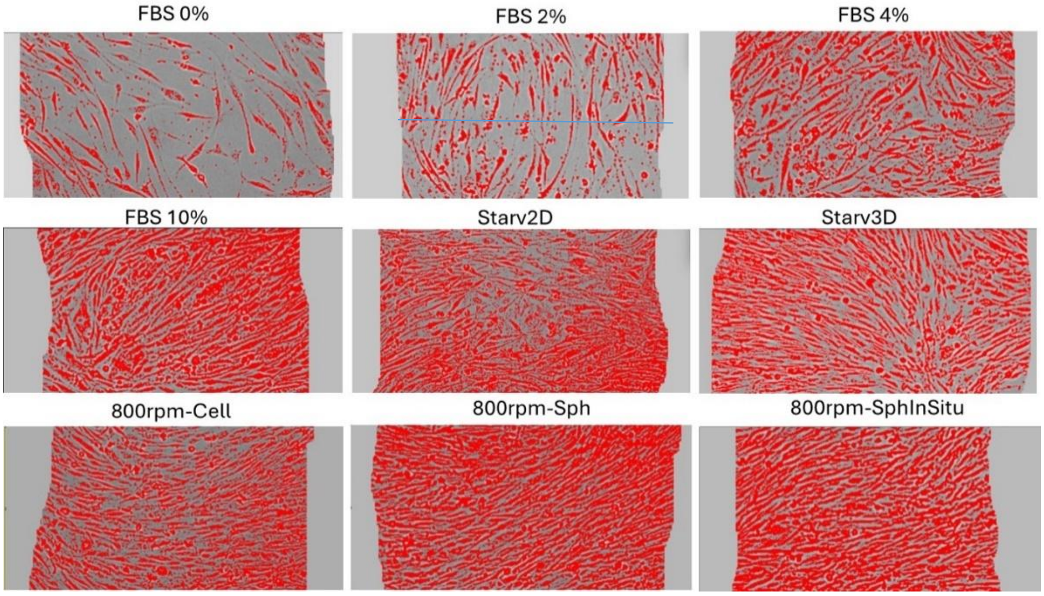

Figure S23: Example of thresholding for wound healing.
